# Hydrogen-bonding behavior of amidines in helical structure†

**DOI:** 10.1039/d4sc06108j

**Published:** 2024-10-23

**Authors:** Emily A. O'Brien, Jeffrey A. Purslow, Brendan J. Wall, Brett VanVeller

**Affiliations:** a Department of Chemistry, Iowa State University Ames IA 50011 USA bvv@iastate.edu

## Abstract

Amidines are an isostere of the amide bond and are completely unexplored in peptide secondary structure. This study marks the first investigation of the structural implications of amidines in folded helices. Amidines were found to engage in hydrogen-bonding interactions that are compatible with helical structure. The protic state of the amidine is also adaptive to local interactions, able to form stronger hydrogen bonds with proton donors or form the first example of a salt bridge along the peptide backbone to stabilize the C-terminus of the helical fold. The rationalization of this behavior was aided by our discovery that the basicity of amidines within peptide backbones can be significantly lower than previously assumed for small molecules. These findings compel investigation of amidines in peptide-drug design.

## Abstract 2

Amidines are an isostere of the amide bond and are completely unexplored in peptide secondary structure.

## Graphical abstract

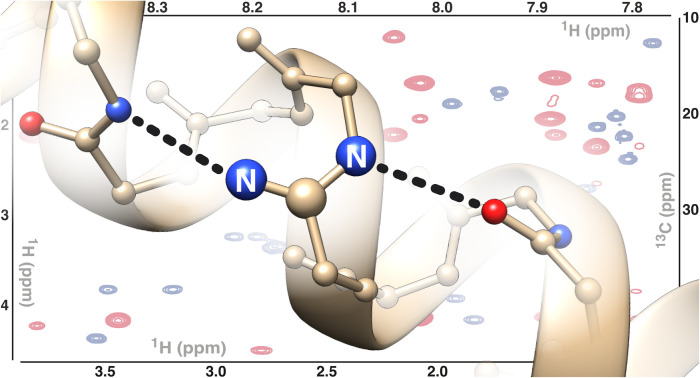

## 1. Introduction

The prevalence of post-translational modifications in Nature suggests that expanding beyond the suite of functional groups found in the 20 canonical amino acids can impart specialized utility to peptide-based biomolecules.^1^ Alteration of the peptide bond—the defining feature^2^ of the molecule—represents a far more rarefied modification. While numerous motifs have been investigated as surrogates for the amide bond in α-helices,^3–7^ these substitutions almost always have a destabilizing effect on the structure^8,9^ except in very specific contexts.^10–12^ However, one peptide-bond isostere in particular, the amidine, has received almost no attention in peptidic molecules.^13–15^

**Fig. 1:**
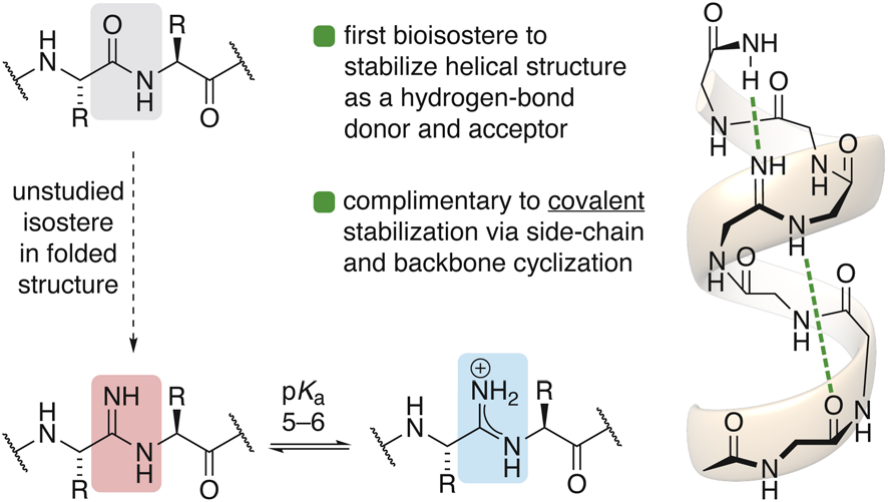
Single-atom substitution with amide-like behavior.

Amidines are distinguished from classical peptide bonds by the substitution of the carbonyl oxygen with a nitrogen atom.^16^ Amidines can be found within a family of natural products called ribosomally synthesized and post-translationally modified peptides (RiPPs), suggesting an evolutionary benefit may have driven the generation of biosynthetic machinery for their installation.^17–19^ Alternatively, amidines have also been proposed as primordial intermediates for the prebiotic synthesis of peptides.^20–22^ Despite these examples from Nature, studies describing the behavior of amidines in synthetic peptides are noticeably scarce.^15,23^ This unique, one-atom substitution, however, carries major implications for basicity and hydrogen-bonding behavior in the rational design of peptides (Fig. 1).^24–27^

The dearth of synthetic amidinopeptide examples derives from a historic lack of methods to insert amidines into peptides without side reactivity. We recently reported a synthetic approach that allowed for the first generally applicable method to site-selectively insert amidines along the peptide backbone.^23,28^

This study represents the first report elucidating the behavior of amidines in folded protein secondary structure. This work challenges long held assumptions about the basic character of amidines and provides the first spectroscopic evidence for the malleable protic states of amidines in response to their local environment, which carries major implications for the design and drug action of vancomycin derivatives against resistant bacteria.^13,14^ Amidines are the first peptide-bond isostere to stabilize helical structure as a hydrogen-bond donor and acceptor. This single-atom substitution provides a non-covalent strategy to stabilize helices in a complimentary way to existing covalent strategies that exploit side-chain and backbone macrocyclization.^29–31^

## 2. Results and discussion

### 2.1 C-terminal effects of amidines in α-helices

**Fig. 2:**
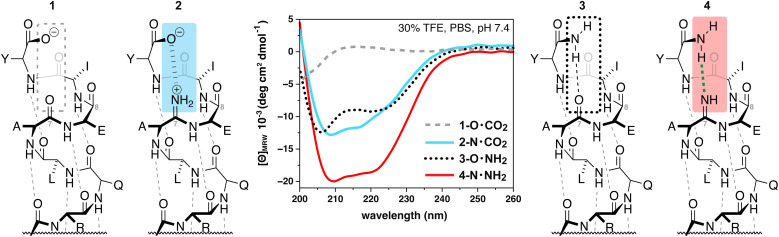
Helical propensities of model peptide Ac-QVARQLAEIY-X displaying different C-terminal chemistries and interactions with the backbone, where X = OH for 1 and 2 and X = NH_2_ for 3 and 4. The amidine in 1 and 4 was placed between Ala_7_ and Glu_8_.

To begin the investigation of how amidines might behave within canonical secondary structure, we chose to study a model α-helix first reported by Arora and coworkers (Table 1).^34^ We were particularly interested in how the dynamic protic states of amidines might affect helicity in response to different chemistries at the C-terminus. Consider peptide 1, which displays a C-terminal carboxylate. At pH 7.4 (phosphate buffer with 30% TFE)^34^1 displays no secondary structure, as evinced by the circular dichroism (CD) spectrum of 1 that shows signatures associated with a random coil (grey-dashed line, Fig. 2). This result is not surprising given the interaction between the C-terminal carboxylate and the oxygen of the amide at Ala_7_ is repulsive. We hypothesized that this interaction could be rendered attractive and stabilize the helical fold if the amide of Ala_7_ were replaced with a primary amidine that, upon protonation, could form the first example of a stabilizing, backbone-to-backbone salt bridge. Accordingly, analysis of 2, a peptide that supplies an amidine suitably placed to complement the negatively-charged carboxylate with a positively-charged amidinium, revealed a more structured peptide than 1, with helical character indicated by negative absorbance at 222 nm in the CD spectrum (blue line, Fig. 2).

**Table 1:** Sequence and circular dichroism data for controls and amidinopeptides

Peptide	Sequence	Solvent^a^	[*θ*]_222_	[*θ*]_208_	% Helicity
1 b	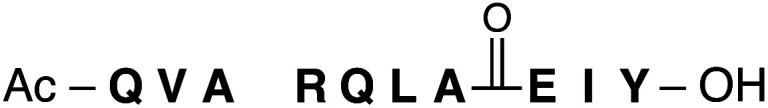	PBS	−1765	−3016	na
		30% TFE	−610	−1145	na
2	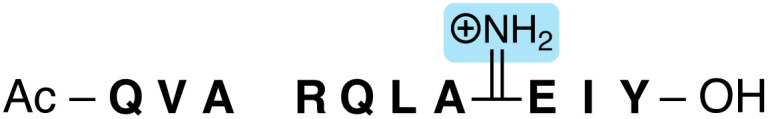	PBS	−1944	−5686	8
		10% TFE	−2153	−6960	9
		20% TFE	−7364	−10174	31
		30% TFE	−9735	−12773	42
3	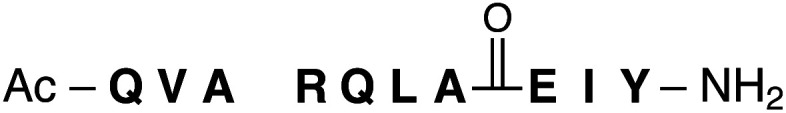	PBS	−4261	−7765	18
		10% TFE	−1544	−4998	17
		20% TFE	−7415	−10082	31
		30% TFE	−9113	−11738	38
4	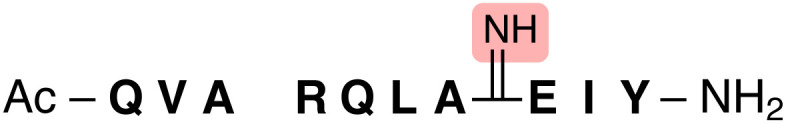	PBS	−2166	−5687	9
		10% TFE	−4740	−8972	20
		20% TFE	−8435	−11136	36
		30% TFE	−15844	−18075	67
5	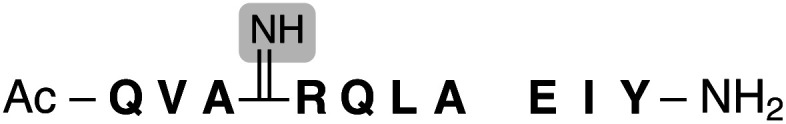	PBS	−1302	−2644	5
		10% TFE	−3338	−4016	14
		20% TFE	−7559	−8646	32
		30% TFE	−12624	−14677	54

a 7.4 pH PBS buffer with increasing %v/v of TFE.

b 1 remained unstructured even with the addition of TFE. See ESI, Fig. S55, for traces of all peptides at all concentrations of TFE. All peptide concentrations were standardized to the absorbance of the tyrosine residue.^32,33^ Error in measurement was 5%. The percent helicity was determined by the ratio of [*θ*]_222_/[*θ*]_max_ at 20 °C. The [*θ*]_max_ was determined using −23400 at a temperature of 20 °C, see eqn (2) in the ESI.

Alternatively, when the C-terminal chemistry of the initial model peptide was changed to a primary amide, 3, the peptide maintained some helical character, presumably because an intra-strand hydrogen bond stabilizes the helical fold at the C-terminus (dotted line, Fig. 2). Remarkably, we observed that the helicity of the peptide was even further stabilized by the presence of an amidine at Ala_7_ in 4 (red line, Fig. 2). We hypothesize that this increased helical propensity was due to the stronger basicity of the amidine relative to the native amide, resulting in a stronger hydrogen bond as a result. Finally, the amidines under consideration were stable during this and all subsequent analyses (Fig. S49†) and do not significantly impact CD absorbance (Fig. S50†).

### 2.2 The pH-dependent behavior of amidines in peptides

**Fig. 3:**
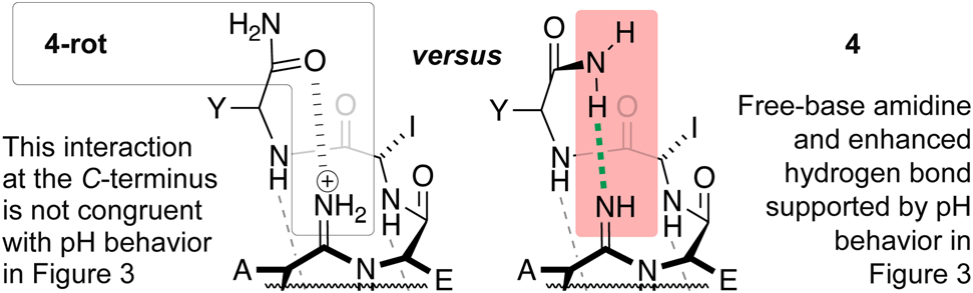
Potential C-terminal interactions leading to helix stabilization. The interaction described in 4 is more consistent with the pH behavior in Fig. 4.

An alternative explanation for the increased helicity of 4 could be rationalized in the context of structure 4-rot (Fig. 3), in which an amidinium donates a charge-enhanced hydrogen bond to the oxygen of the C-terminal primary amide—a simple bond rotation.

**Fig. 4:**
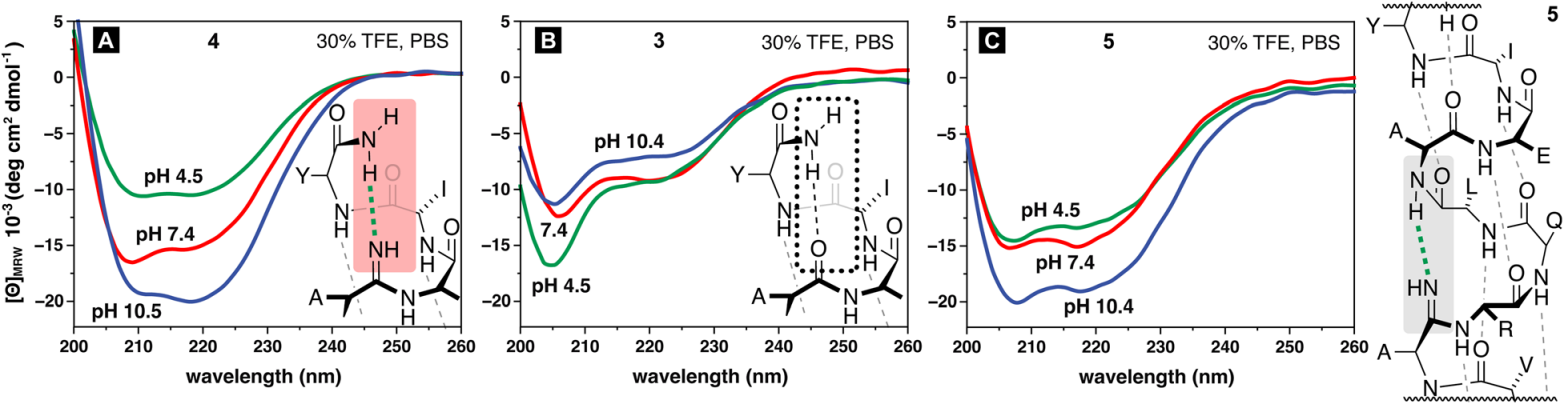
pH-dependent behavior and helical propensities of model peptides based on Ac-QVARQLAEIY-NH_2_. CD curves measured at 20 °C for (A) peptide 4 (B) peptide 3, and (C) peptide 5 in PBS buffer at pH 4.5, 7.4, and 10.5 with the addition of 30% TFE as a co-solvent. The amidine in 4 and 3 was placed between Ala_7_ and Glu_8_. The amidine in 5 was placed between Ala_3_ and Arg_4_.

To probe for this possibility, the pH of the solution was varied and the helicity of 4 was found to decrease under acidic pH and increase at more basic pH (Fig. 4A). No substantive changes in the helicity of the native all-oxoamide control peptide 3 were observed upon varying the pH over the same range (Fig. 4B), confirming that changes in helicity were likely due to the influence of the amidine moiety in 4 and not due to side-chain interactions. This behavior supports the notion of a free-base amidine, whereupon protonation of the amidine at acidic pH would disrupt the stabilizing C-terminal interaction in 4.^15^ Alternatively, basic pH would promote the free-base form of the amidine, stabilizing the helical fold in 4. These results, however, do not align with the interaction described in 4-rot (Fig. 3).

The results above, and the proposed hydrogen-bonding interaction, led us to hypothesize that the amidine could be moved to a more internal residue within the helix without significant compromise to the helical fold. Accordingly, when the amidine was placed in between Ala_3_ and Arg_4_ in the sequence, we observed a prominent helical signature in the CD spectrum for peptide 5 (Fig. 4C). Notably, the internal amidine in peptide 5 led to greater helicity than the native sequence in 3 (Fig. 4C*versus*4B). The behavior of 5 in response to changing pH mirrors that of 4. Lower pH led to a modest decrease in helicity as determined by CD, while increased pH presumably favored the free-base of the amidine, promoting a stronger hydrogen-bonding interaction and greater observed helicity (Fig. 4C). Differences in the pH-dependent behavior between 4 and 5 likely arise due to the amidine in 4 being placed in the more disordered and solvent-exposed C-terminus compared to the more internal position in the helix for 5.

The combined results of both peptides 2 and 4 reveal that amidines are an amide-bond isostere that is tolerated in folded α-helical secondary structure and, moreover, can confer greater helical character due to a stronger hydrogen bonding interaction conferred by the more basic amidine.

### 2.3 Basicity and protic state of amidines in peptides

#### 2.3.1 Basicity of amidines in peptides

**Fig. 5:**
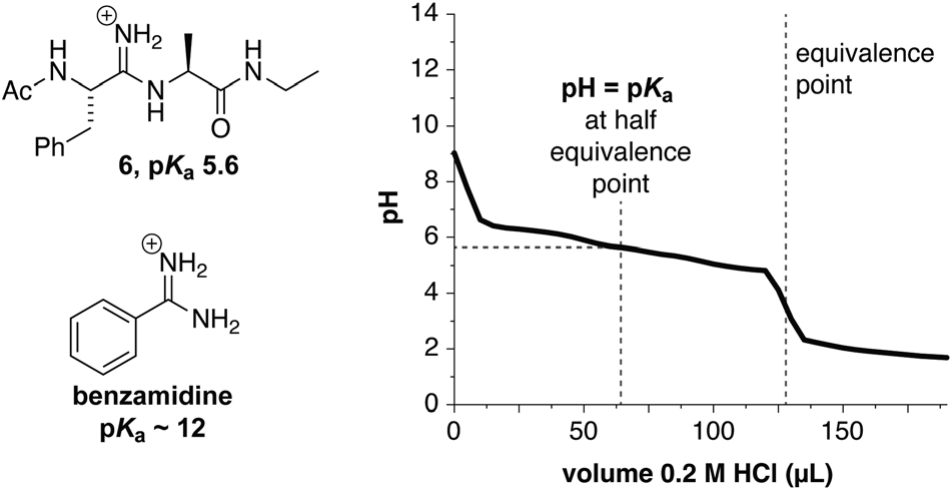
Titration experiment to estimate the p*K*_a_ of an amidine in peptide structure and comparison to a more solvent-exposed benzamide.

The pH-dependent behavior of amidines described above and corresponding proposal of the ‘free-base’ protic state of 4 led us to reconsider some broad assumptions about amidine basicity. The amidine is generally considered to be highly basic (p*K*_a_ ≈ 12), where these charged groups are associated with poor pharmacokinetic properties in small molecules.^26,35,36^ These assertions appear to be supported by the reported p*K*_a_ values for benzamidine and drug molecules in which the amidine decorates the periphery of the core.^26,37^ Thus, amidines have traditionally been studied in settings in which solvent exposure promotes a protonated state. In contrast, an amidine within a peptide backbone would be significantly more buried and solvated to a lesser degree. Accordingly, we measured a p*K*_a_ between 5 and 6 when an amidine was placed within an Ac-Phe-Ala-NHEt dipeptide (6, Fig. 5). It should be noted that opportunities for intramolecular hydrogen bonding of the amidine in 6 with the amides that bracket it can lead to lower measured p*K*_a_ values.^38^ Thus, dipeptide 6 should be regarded as a lower limit of the p*K*_a_ in folded helical structure.

The potential for more variable basic properties for amidines is in stark contrast to the related guanidine functional group in the side-chain of arginine, which has been spectroscopically demonstrated to be invariably protonated and charged (p*K*_a_ > 12) in proteins, even when buried within hydrophobic microenvironments.^39^ This new understanding of amidine p*K*_a_ behavior, however, lends additional support to the work of Boger and coworkers concerning the adaptive protic states of amidines in the function of so-called maxamycins against antibiotic resistant Gram-positive bacteria.^40,41^

#### 2.3.2 NMR characterization of amidine protic state

To further characterize the protic state of the amidine by NMR, we next exploited our synthetic approach^28^ for facile installation of ^15^N (from relatively inexpensive ^15^NH_4_OAc) at the ^2^*N*-imino position of the amidine. This is a notable advantage of amidines compared to the amide or traditional ester and thioamide isosteres. Facile installation of ^15^N at the ^2^*N*-imino position of the amidine negates the need for costly ^15^N enriched amino acids to introduce this probe nucleus into amides and thioamides. Further, the amidine furnishes a spin 1/2 ^15^N nucleus to probe local structure and coordination in solution-phase experiments, in contrast to oxygen and sulfur nuclei which are both quadrupolar and only amenable to solid-state NMR experiments.^42,43^ Moreover, the prominent signal from the ^15^N spin label confirms the installation of the amidine along the peptide backbone and provides further confidence in the success of the synthesis.

**Fig. 6:**
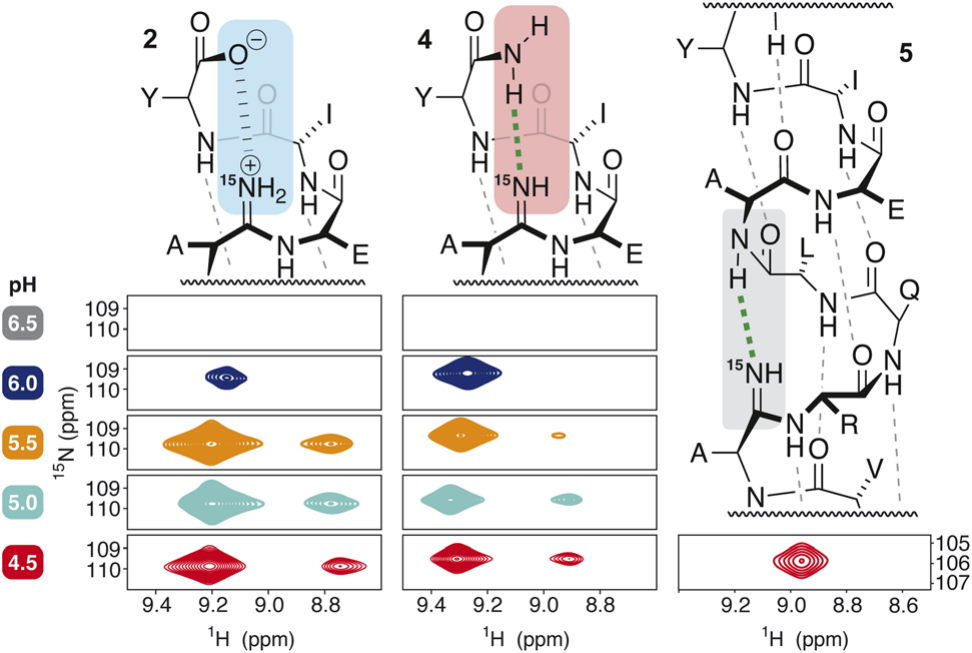
NMR pH titration of model peptides at 20 °C in PBS buffer with the addition of 30% TFE as a co-solvent.

Spectroscopic evidence of the protic states of the amidine in all three peptides—2, 4, and 5—was gathered using ^15^N–^1^H HSQC correlation spectroscopy (Fig. 6). The chemical shift of the enriched ^15^N in the amidine was easily identifiable as a result of its signal intensity relative to the other amides at natural abundance.

**Fig. 7:**
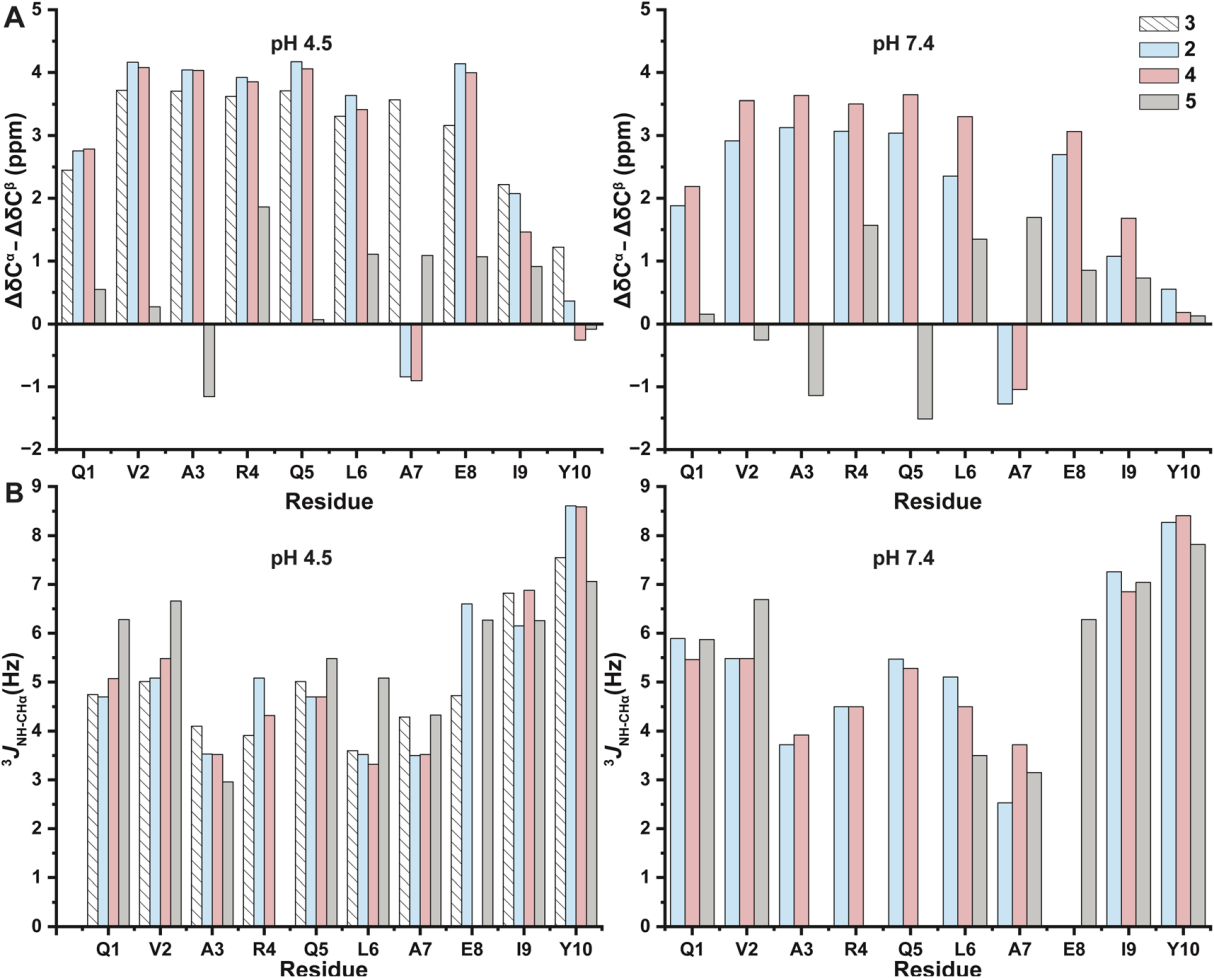
Secondary structure analysis for 2, 3, 4, and 5 using (A) the difference between experimentally observed ^13^C^α^ and ^13^C^β^ chemical shifts and reference shifts for a random coil at pH 4.5 (left) and pH 7.4 (right) and (B) the ^3^*J*_HN-CH^α^_ coupling constants at pH 4.5 (left) and pH 7.4 (right).^44,45^

To limit effects due to fast exchange of protons, initial experiments were conducted at pH 4.5, where all peptides were shown to have helical character (by CD (Fig. 4) and by NMR (Fig. 7*vide infra*)). For the C-terminal amidines in 2 and 4, we observed two cross peaks for the ^15^N label at 109 ppm, indicating that two ^1^H were bound to the ^2^*N*-imino nitrogen of the amidine at pH 4.5. This NH_2_^+^ protonation state supports the notion of an intra-strand salt bridge proposed for 2. Given the p*K*_a_ value of 5–6 we estimated for an amidine in a peptide backbone (Fig. 5), the observation of a doubly protonated amidine in 4 at pH 4.5 was not unexpected. These observation, however, are in marked contrast to the results for 5 (Fig. 6), which displayed only one crosspeak, indicating the unprotonated, free-base form. We hypothesize that the double protonation of the amidine in 4 arises from its position in the more disordered C-terminus of the peptide (Fig. 7), leading to greater solvent exposure, providing more opportunity for hydrogen-bonding interactions with protons from solvent. The amidine in 5, however, is in the middle of the helix and appears to be protected from solvation.

We next attempted to raise the pH to see if we could observe changes in the protic state for 2 and 4 (Fig. 6). Unfortunately, fast-exchange processes lead to loss of the ^15^N–^1^H cross peaks within only 2 pH units, which limited the scope of the analysis. This observation is not uncommon in protein NMR, and lowering the temperature of the analysis to 5 °C did not restore the signal. Several interesting observations still provided insight into the structure however. In the case of 2, the ratio of the two cross peaks remains constant as the pH is increased, until the signals are lost to fast exchange at pH 6.0. We propose that this observation supports the presence of an NH_2_^+^ moiety that persists as pH increases, consistent with our proposed intra-strand salt bridge 2. Conversely, for 4 the cross peak for one of the protons on the ^15^N loses intensity as the pH increases, accompanied by a shift in the remaining proton, until complete loss of signal at pH 6.5. We propose that this observation supports the notion of a mono-protonated NH moiety that becomes protonated to a greater extent at lower pH, consistent with our proposed free-base amidine in 4.

### 2.4 NMR characterization of helical structure

This study is the first to characterize the behavior of amidines in folded peptide structure. Thus, to both further interrogate the results of the CD experiments and identify the effects of amidine incorporation on NMR secondary-structure analysis, ^13^C–^1^H and ^15^N–^1^H HQSCs, NOESY, and TOCSY experiments were used to fully assign the chemical shifts of 2, 4, and 5 (see Tables S1 and S2 in ESI† for residue assignments).

Analysis of the high-field (800 MHz) 2D-NOESY NMR data was used to confirm peptide helicity. NOE correlations along the peptide backbone are key signatures of helical structure (Table 2). Specifically, sequential (NN) and _α_N (*i*, *i* + 1) and non-sequential _α_N (i, *i* + 3) cross-peaks are indisputable evidence for helical structure. Although spectral overlap inhibits the observation of all cross-peaks, a significant number of signature cross-peaks were observed for the peptides under investigation, further validating helical structure. NOE correlation maps and an NOE correlation table are provided in ESI (Fig. S36 and S39†).

**Table 2:** Observable NOE correlation cross-peaks along the backbone for sequential NN (*i*,*i* + 1) and d_α_N (*i*,*i* + 1) crosspeaks. In addition to the non-sequential medium range NOEs, d_α_N (*i*,*i* + 3) and d_α_N (*i*,*i* + 4) to support α-helical conformation for amidinopeptides at pH 4.5 and 7.4

Peptide	pH 4.5				pH 7.4			
	NN	d_α_N (*i*,*i* + 1)	d_α_N (*i*,*i* + 3)	d_α_N (*i*,*i* + 4)	NN	d_α_N (*i*,*i* + 1)	d_α_N (*i*,*i* + 3)	d_α_N (*i*,*i* + 4)
2	Q1 → V2	Q1 → V2	V2 → Q5	V2 → L6	Q1 → V2	Q1 → V2	V2 → Q5	
	V2 → A3	V2 → A3	A3 → L6		V2 → A3	V2 → A3	A3 → L6	
	R4 → Q5	Q5 → L6	R4 → A7		R4 → Q5	Q5 → L6	R4 → A7	
	Q5 → L6	L6 → A7	L6 → I9		L6 → A7	L6 → A7		
	L6 → A7	E8 → I9	A7 → Y10		I9 → Y10	E8 → I9		
	A7 → E8	I9 → Y10				I9 → Y10		
	E8 → I9							
	I9 → Y10							
4	Q1 → V2	Q1 → V2	V2 → Q5	V2 → L6	Q1 → V2	Q1 → V2	Q1 → R4	V2 → L6
	V2 → A3	R4 → Q5	A3 → L6		V2 → A3	V2 → A3	V2 → Q5	
	R4 → Q5	Q5 → L6	R4 → A7		R4 → Q5	L6 → A7	R4 → A7	
	Q5 → L6	L6 → A7	Q5 → E8		Q5 → L6	I9 → Y10	L6 → I9	
	L6 → A7	E8 → I9	L6 → I9		L6 → A7			
	A7 → E8	I9 → Y10			I9 → Y10			
	E8 → I9							
	I9 → Y10							
5	Q1 → V2	Q1 → V2			L6 → A7	Q1 → V2	A7 → Y10	
	V2 → A3	V2 → A3			A7 → E8	E8 → I9		
	Q5 → L6	Q5 → L6			E8 → I9	I9 → Y10		
	L6 → A7	L6 → A7			I9 → Y10			
	E8 → I9	E8 → I9						
		I9 → Y10						

Following the chemical shift assignments, we characterized the residue-specific helicity within each peptide by two independent ways. The first method used the differences between the experimentally observed ^13^C^α^ and ^13^C^β^ chemical shifts and the corresponding chemical shifts for a random coil.^46,47^^13^C^α^ and ^13^C^β^ chemical shifts have been shown to be extremely sensitive and accurate indicators of secondary structure, even more accurate than H^α^ chemical shifts when distinguishing an α-helix from a random coil.^46–48^ When comparing experimental chemical shifts against corresponding random coil chemical shifts, positive Δ*δ*C^α^–Δ*δ*C^β^ values are indicative of α-helical structure (and negative Δ*δ*C^α^–Δ*δ*C^β^ values indicate β-sheet structure).^49–51^ Thus, based on this method of analysis, both 2 and 4 display helical structure along the sequence with a notable exception for Ala_7_, which is the residue that bears the amidine for both peptides (Fig. 7A). The distinctly negative Δ*δ*C^α^–Δ*δ*C^β^ value for Ala_7_ in these peptides is not reliable for secondary structure analysis, however, because the corresponding random-coil chemical shifts are referenced for native oxoamide amino acids. Thus, the chemical shift of an Ala_7_ amidine-containing amino acid is therefore not suitable for the analysis *via* these methods.^52,53^

The aberrant Δ*δ*C^α^–Δ*δ*C^β^ chemical shift values imposed by the amidine functional group have substantially greater impact on the analysis of 5, which bears the internal amidine at Ala_3_ (Fig. 7). The magnitude of the Δ*δ*C^α^–Δ*δ*C^β^ values are generally lower relative to 2 and 4 across the sequence and highly variable around the amidine itself. The negative Δ*δ*C^α^–Δ*δ*C^β^ for Gln_7_ is particularly noteworthy and not congruent with the CD data described above. Because the Δ*δ*C^α^–Δ*δ*C^β^ analysis is based on empirically derived chemical shifts for α-helices, it is unsurprising that these results are not appropriate to a novel and non-standard backbone modification such as the amidine. We therefore sought to interrogate the structure using a different spectroscopic signature that was not dependent on chemical shift.

The ambiguities with respect to differences in the chemical shift displayed by the amidine compared to the native amide drove us to pursue a second method to characterize the secondary structure. Specifically, the dihedral angle between the amide NH and the CH^α^ (the *ϕ* angle) is another signature of helical structure and is independent of chemical shift. In a freely rotating, random coil, the ^3^*J*_HN-CH^α^_ coupling for the *ϕ*-angle is >6 Hz. The *ϕ*-angle in an α-helix, however, is an acute −57°, which corresponds to a ^3^*J*_HN-CH^α^_ coupling value of 3–6 Hz (the ^3^*J*_HN-CH^α^_ coupling value in a β-sheet conformation is >8 Hz).^54^

Thus, the residue-specific ^3^*J*_HN-CH^α^_ values for each peptide 2, 4, and 5 indicated helical structure along the sequence (Fig. 7B and Table S3†). Residues near the N- and C-termini are likely to be the most disordered, as indicated by ^3^*J*_HN-CH^α^_ values that are >6 Hz. Importantly, Ala_7_ in both 2 and 4 displayed helical character, in agreement with the CD experiments (Fig. 2), and confirming our suspicions that the negative Δ*δ*C^α^–Δ*δ*C^β^ values with respect to Ala_7_ in Fig. 7A were an artifact of the unique chemical shifts of the amidine in that particular difference analysis.

The ^3^*J*_HN-CH^α^_ coupling value for Ala_3_ in 5 similarly indicates a helical conformation. Residues on the N-terminal side of Ala_3_ (Gln_1_ and Val_2_) are more disordered than the analogous residues in 2 and 4. Residues on the C-terminal side of Ala_3_ in 5 (Arg_4_ and Gln_5_) are subject to fast-exchange process that broaden these signals below the limit of detection and prevent analysis. These exchange processes are known to increase in severity as the pH of the sample approaches 7. Accordingly, we observe loss of signal for Ala_3_, Arg_4_ and Gln_5_ at pH 7.4 (Fig. 7B). These exchange processes may indicate that the peptide is sampling different conformations, perhaps exploring an *i* + 4 → *i* hydrogen bond between Leu_6_ and Ala_3_ (α-helical) and an *i* + 3 → *i* hydrogen bond between Gln_5_ and Ala_3_ (3_10_ helix). This conformational change would impact the intervening Arg_4_ and Gln_5_ most significantly.

## 3. Conclusion

This work is the first study to investigate the structural implications of amidines in helical peptide structure. Amidines are an historically ignored amide-bond isostere, but recently reported methods for installation into peptide backbones^23,28,55^ now make it possible to investigate these groups. Collectively, the results of this work indicate that amidine isosteres are not only tolerated in folded helical structure, but make interactions that are intuitive and amenable to design. These findings compel investigation of amidines in peptide-drug design, particularly in light of the fact that the p*K*_a_ of amidines in peptides may be lower than previously assumed, thereby improving the potential pharmacokinetic profile.

More specifically, helicity is most tolerant of amidines at the C-terminus, likely due to the greater flexibility associated with the fraying ends of the helix. Depending on the C-terminal chemistry, the amidine can adapt its protic state to its local environment. In the case of a C-terminal carboxylate, an amidinium forms the first example of an intrachain salt bridge. Alternatively, in the case of a C-terminal hydrogen-bond donor, a free-base amidine can form a stronger hydrogen bond than a traditional amide to stabilize the helical fold. The amidine can also be inserted in the middle of the peptide without detrimental loss of overall helical character, although the residues around the amidine may experience greater disorder.

Evidence for the nature of the protic state of the amidine was greatly aided by the adventitious spin 1/2 ^15^N nucleus that could be readily inserted during peptide synthesis. The ability to probe local interactions around a particular backbone site using solution-phase NMR techniques makes amidines a powerful structural tool.

## 4. Experimental

All experimental details and characterization data are provided free of charge in the electronic ESI.†

## Author contributions

B. V., E. A. O. and B. J. W. conceived of the work. E. A. O collected and analyzed CD data. J. A. P. collected and analyzed NMR data with E. A. O. All authors contributed to the writing and proofing of the final manuscript.

## Conflicts of interest

There are no conflicts to declare.

## Supplementary Material

SC-015-D4SC06108J-s001

## Data Availability

Data for this article, including synthetic and experimental procedures, CD and NMR spectroscopic data for characterization of conformation and structure, and other supporting experiments has been included in the ESI† and available free of charge on the publisher's website for this article.
