# Supplementary material for: Hydrogen-bonding behavior of amidines in helical structure
Source: Chem Sci. 2024 Oct 23;15(45):18992–9. doi: 10.1039/d4sc06108j (PMC11514129; doi:10.1039/d4sc06108j)
Supplement: SC-015-D4SC06108J-s001 [file SC-015-D4SC06108J-s001.pdf]

Supplementary Information (SI) for Chemical Science.
This journal is © The Royal Society of Chemistry 2024

Electronic Supporting Information

# Hydrogen-Bonding Behavior of Amidines in Helical Structure

Emily A. O'Brien, Jeffrey A. Purslow, Brendan J. Wall, and Brett VanVeller*
Department of Chemistry, Iowa State University, Ames, IA 50011, USA
email: bvv@iastate.edu

## Contents

**1 General Information** **S4**
1.1 Materials . . . S4
1.2 Experimental . . . S4

**2 Synthetic Methods** **S5**
2.1 Synthesis Synthesis of Thioacylating Reagents (Fmoc-$X_{aa}$$^{(S)}$-Nbt) . . . S5
2.2 Synthesis of Amidine Dipeptide (**6**). . . . S5
2.2.1 Synthesis of Boc-Phe$^{(S)}$Ala-OMe. . . . S5
2.2.2 Synthesis of Ac-Phe$^{(SMe)}$-Ala-NHEt. . . . S5
2.2.3 Synthesis of Ac-Phe$^{(NH)}$-Ala-NHEt (**6**). . . . S6
2.3 General Wang Resin Loading . . . S6
2.4 General Peptide Synthesis . . . S6
2.4.1 Thioamide Incorporation . . . S6
2.4.2 Thioamide Protection . . . S7
2.4.3 Amidine Formation . . . S7
2.4.4 Isolation and Purification . . . S7

**3 Peptide NMR assignment** **S8**

**4 NMR Spectra** **S10**
4.1 Dipeptide NMR spectra . . . S10
4.2 Peptide NMR data . . . S12
4.2.1 Peptide NMR parameters . . . S12
4.2.2 NMR spectra of Amidinopeptides at pH 4.5 . . . S12
4.2.3 NMR spectra of Amidinopeptides at pH 7.4 . . . S21
4.2.4 Example Spectra of additional NMR experiments . . . S27

**5 Structure Determination *via* NMR.** **S28**
5.1 chemical shift in difference of $\alpha$-protons . . . S28
5.2 J-coupling experiments of amidinopeptides . . . S29
5.3 NOE cross peak correlations for Amidinopeptides . . . S30

**6 HPLC Traces of Purified Peptides** **S34**
6.1 purity check traces of peptides . . . S34
6.2 Stability of Amidinopeptide in Water. . . . S37
6.3 Absorbance Difference of Acetamide and Acetamidine . . . S38

**7 Procedure for p$K_a$ determination of 6** **S39**

**8 CD Experiments** **S40**

**9 References** **S41**

## List of Figures


S1 600 MHz $^1$H NMR spectra Ac-Phe$^{(SMe)}$-Ala-NHEt in $CDCl_3$ . . . S10
S2 150 MHz $^{13}$C NMR spectra Ac-Phe$^{(SMe)}$-Ala-NHEt in $CDCl_3$ . . . S10
S3 600 MHz $^1$H NMR spectra Ac-Phe$^{(NH)}$-Ala-NHEt (**6**) in $D_2O$ . . . S11
S4 150 MHz $^{13}$C NMR spectra Ac-Phe$^{(NH)}$-Ala-NHEt **(6)** in $CDCl_3$ for referencing . . . S11
S5 700 MHz $^{15}$N-H HSQC spectrum (293K) of AcQVARQLA$^{NH}$EIY-$CO_2$, Peptide **2** in $D_2O$/TFE-$d_2$/PBS (5/30/65%v/v) at pH 4.5 S12
S6 700 MHz $^{13}$C-H HSQC spectrum (293K) of AcQVARQLA$^{NH}$EIY-$CO_2$, Peptide **2** in $D_2O$/TFE-$d_2$/PBS (5/30/65%v/v) at pH 4.5 S13
S7 700 MHz finger print region of NOESY spectrum (293K) of AcQVARQLA$^{NH}$EIY-$CO_2$, Peptide **2** in $D_2O$/TFE-$d_2$/PBS (5/30/65%v/v) at pH 4.5 . . . S13
S8 700 MHz finger print region of TOCSY spectrum (293K) of AcQVARQLA$^{NH}$EIY-$CO_2$, Peptide **2** in $D_2O$/TFE-$d_2$/PBS (5/30/65%v/v) at pH 4.5 . . . S14
S9 800 MHz finger print region of NOESY spectrum (293K) of AcQVARQLAEIY-$NH_2$, Peptide **3** in $D_2O$/TFE-$d_2$/PBS (5/30/65%v/v) at pH 4.5 . . . S14
S10 800 MHz finger print region of TOCSY spectrum (293K) of AcQVARQLAEIY-$NH_2$, Peptide **3** in $D_2O$/TFE-$d_2$/PBS (5/30/65%v/v) at pH 4.5 . . . S15
S11 800 MHz $^{15}$N-H HSQC spectrum (293K) of AcQVARQLAEIY-$NH_2$, Peptide **3** in $D_2O$/TFE-$d_2$/PBS (5/30/65%v/v) at pH 4.5 . S15
S12 800 MHz $^{13}$C-H HSQC spectrum (293K) of AcQVARQLAEIY-$NH_2$, Peptide **3** in $D_2O$/TFE-$d_2$/PBS (5/30/65%v/v) at pH 4.5 . S16
S13 700 MHz $^{15}$N-H HSQC spectrum (293K) of AcQVARQLA$^{NH}$EIY-$NH_2$, Peptide **4** in $D_2O$/TFE-$d_2$/PBS (5/30/65%v/v) at pH 4.5 S16
S14 700 MHz $^{13}$C-H HSQC spectrum (293K) of AcQVARQLA$^{NH}$EIY-$NH_2$, Peptide **4** in $D_2O$/TFE-$d_2$/PBS (5/30/65%v/v) at pH 4.5 S17
S15 700 MHz finger print region of NOESY spectrum (293K) of AcQVARQLA$^{NH}$EIY-$NH_2$, Peptide **4** in $D_2O$/TFE-$d_2$/PBS (5/30/65%v/v) at pH 4.5 . . . S17
S16 700 MHz finger print region of TOCSY spectrum (293K) of AcQVARQLA$^{NH}$EIY-$NH_2$, Peptide **4** in $D_2O$/TFE-$d_2$/PBS (5/30/65%v/v) at pH 4.5 . . . S18
S17 700 MHz $^{15}$N-H HSQC spectrum (293K) of AcQVA$^{NH}$RQLAEIY-$NH_2$, Peptide **5** in $D_2O$/TFE-$d_2$/PBS (5/30/65%v/v) at pH 4.5 S18
S18 700 MHz $^{13}$C-H HSQC spectrum (293K) of AcQVA$^{NH}$RQLAEIY-$NH_2$, Peptide **5** in $D_2O$/TFE-$d_2$/PBS (5/30/65%v/v) at pH 4.5 S19
S19 700 MHz finger print region of NOESY spectrum (293K) of AcQVA$^{NH}$RQLAEIY-$NH_2$, Peptide **5** in $D_2O$/TFE-$d_2$/PBS (5/30/65%v/v) at pH 4.5 . . . S19
S20 700 MHz finger print region of TOCSY spectrum (293K) of AcQVA$^{NH}$RQLAEIY-$NH_2$, Peptide **5** in $D_2O$/TFE-$d_2$/PBS (5/30/65%v/v) at pH 4.5 . . . S20
S21 700 MHz $^{15}$N-H HSQC spectrum (293K) of AcQVARQLA$^{NH}$EIY-$CO_2$ Peptide **2** in $D_2O$/TFE-$d_2$/PBS (5/30/65%v/v) at pH 7.4 S21
S22 700 MHz $^{13}$C-H HSQC spectrum (293K) of AcQVARQLA$^{NH}$EIY-$CO_2$, Peptide **2** in $D_2O$/TFE-$d_2$/PBS (5/30/65%v/v) at pH 7.4 S21
S23 700 MHz finger print region of NOESY spectrum (293K) of AcQVARQLA$^{NH}$EIY-$CO_2$, Peptide **2** in $D_2O$/TFE-$d_2$/PBS (5/30/65%v/v) at pH 7.4 . . . S22
S24 700 MHz finger print region of TOCSY spectrum (293K) of AcQVARQLA$^{NH}$EIY-$CO_2$ Peptide **2** in $D_2O$/TFE-$d_2$/PBS (5/30/65%v/v) at pH 7.4 . . . S22
S25 700 MHz $^{15}$N-H HSQC spectrum (293K) of AcQVARQLA$^{NH}$EIY-$NH_2$, Peptide **4** in $D_2O$/TFE-$d_2$/PBS (5/30/65%v/v) at pH 7.4 S23
S26 700 MHz $^{13}$C-H HSQC spectrum (293K) of AcQVARQLA$^{NH}$EIY-$NH_2$, Peptide **4** in $D_2O$/TFE-$d_2$/PBS (5/30/65%v/v) at pH 7.4 S23
S27 700 MHz finger print region of NOESY spectrum (293K) of AcQVARQLA$^{NH}$EIY-$NH_2$, Peptide **4** in $D_2O$/TFE-$d_2$/PBS (5/30/65%v/v) at pH 7.4 . . . S24
S28 700 MHz finger print region of TOCSY spectrum (293K) of AcQVARQLA$^{NH}$EIY-$NH_2$, Peptide **4** in $D_2O$/TFE-$d_2$/PBS (5/30/65%v/v) at pH 7.4 . . . S24
S29 700 MHz finger print region of NOESY spectrum (293K) of AcQVA$^{NH}$RQLAEIY-$NH_2$, Peptide **5** in $D_2O$/TFE-$d_2$/PBS (5/30/65%v/v) at pH 7.4 . . . S25
S30 700 MHz finger print region of TOCSY spectrum (293K) of AcQVA$^{NH}$RQLAEIY-$NH_2$, Peptide **5** in $D_2O$/TFE-$d_2$/PBS (5/30/65%v/v) at pH 7.4 . . . S25
S31 700 MHz $^{15}$N-H HSQC spectrum (293K) of AcQVA$^{NH}$RQLAEIY-$NH_2$, Peptide **5** in $D_2O$/TFE-$d_2$/PBS (5/30/65%v/v) at pH 7.4 S26
S32 700 MHz $^{13}$C-H HSQC spectrum (293K) of AcQVA$^{NH}$RQLAEIY-$NH_2$, Peptide **5** in $D_2O$/TFE-$d_2$/PBS (5/30/65%v/v) at pH 7.4 S26
S33 Overlay of pH titration NMR spectra at 20 °C for Peptide **2** and **4** in $D_2O$/TFE-$d_2$/PBS (5/30/65%v/v). . . . S27
S34 800 MHz J-coupling experiment example spectra (293K) of AcQVARQLA$^{NH}$EIY-$CO_2$ Peptide **2** in $D_2O$/TFE-$d_2$/PBS (5/30/65%v/v) at pH 7.4.[6] . . . S27
S35 A) Comparison of peptides **2**, **3**, **4**, and **5** at pH 4.5. B) Comparison of peptides **2**, **4**, and **5** at pH 7.4. All using the $\Delta\delta H_\alpha$ chemical shifts (equation 2). . . . S28
S36 observable NOE correlation cross-peaks along the backbone for non-sequential medium range NOEs, $d\alpha$N (*i*,*i*+3) and $d\alpha$N (*i*,*i*+4) cross peaks to support $\alpha$-helix for amidinopeptide at pH 4.5. . . . S30
S37 observable NOE correlation cross-peaks along the backbone for sequential NN (*i*,*i*+1) and $d\alpha$N (*i*,*i*+1) cross peaks to support $\alpha$-helix for amidinopeptide at pH 4.5. . . . S31

S38 observable NOE correlation cross-peaks along the backbone for sequential NN ($i$,$i$+1) and $d\alpha$N ($i$,$i$+1) cross peaks to support $\alpha$-helix for amidinopeptide at pH 7.4. . . . . S32
S39 observable NOE correlation cross-peaks along the backbone for non-sequential medium range NOEs, $d\alpha$N ($i$,$i$+3) and $d\alpha$N ($i$,$i$+4) cross peaks to support $\alpha$-helix for amidinopeptide at pH 7.4. . . . . S33
S40 HPLC Trace of Peptide **1** AcQVARQLAEIY-OH monitored at 254 nm. . . . . S34
S41 HPLC Trace of Peptide **2** AcQVARQLA$^{NH}$EIY-OH monitored at 254 nm. . . . . S34
S42 HPLC Trace of Peptide **3** AcQVARQLAEIY-$NH_2$ monitored at 254 nm. . . . . S35
S43 HPLC Trace of Peptide **4** AcQVARQLA$^{NH}$EIY-$NH_2$ monitored at 254 nm. . . . . S35
S44 HPLC Trace of Peptide **2** isotopically labeled AcQVARQLA$^{15NH}$EIY-OH monitored at 254 nm. . . . . S35
S45 HPLC Trace of Peptide **4** isotopically labeled AcQVARQLA$^{15NH}$EIY-$NH_2$ monitored at 254 nm. . . . . S36
S46 HPLC Trace of Peptide **5** AcQVA$^{NH}$RQLAEIY-$NH_2$ monitored at 254 nm. . . . . S36
S47 HPLC Trace of Peptide **5** isotopically labeled AcQVA$^{15NH}$RQLAEIY-$NH_2$ monitored at 254 nm. . . . . S36
S48 pH of Peptide 4 to be between 6-7 according to the pH strips shown. Left spot is of Peptide 4 in solution, and the right spot is a solution of sodium bicarbonate. . . . . S37
S49 UPLC trace using gradient from Table S5 of **A)** peptide **4** initial (black) and after sitting in a water solution with a pH from 6-7 for 9 days (red). Showing little to no hydrolysis of the amidinopeptide and formation of native oxoamide peptide (**3**, dotted black trace). **B)** peptide **2-N·OH** initial (black) and after sitting in a water solution with a pH from 6-7 for at temperatures of 50°C and 80°C. Showing little hydrolysis of the amidinopeptide and formation of native oxoamide peptide (**1-O·OH**, dotted black trace) at these condition, specifically at the higher temperature of 80°C. These observation at slightly milder conditions than what has been reported in the literature from conversion of amidines to amide.[8–10] . . . . . S37
S50 UV-VIS absorption spectra of Acetamide (Black) vs Acetamidine (Red). . . . . S38
S51 pH titration curve for imidazole giving a p$K_a$H of 6.9. To standardize the method to determine the p$K_{aH}$ of **6**. . . . . S39
S52 CD Spectra of **A)** peptide 1 vs **B)** peptide 2, **C)** peptide 3-O·$NH_2$ vs **D)** peptide 4-N·$NH_2$. and **E**) peptide 5. **F)** thermal decomposition of amidinopeptides (4-N·$NH_2$ and 5). . . . . S41

## List of Tables

S1 NMR $^1$H, $^{13}$C, and $^{15}$N assignments and chemical shifts ($\delta$ ppm) for peptides **2**, **3** **4** and **5** at 293K, pH 4.5 in $D_2O$/TFE-$d_2$/PBS (5/30/65) . . . . . S8
S2 NMR $^1$H, $^{13}$C, and $^{15}$N assignments and chemical shifts ($\delta$ ppm) for peptides **2**, **4** and **5** at 293K, pH 7.4 in $D_2O$/TFE-$d_2$/PBS (5/30/65) . . . . . S9
S3 $J$-Coupling (Hz) of backbone amide protons for peptides and calculated $\varphi$ angle for amidinopeptides in main manuscript at 293K, pH 4.5 and 7.4 in $D_2O$/TFE-$d_2$/PBS (5/30/65 %v/v). Utilizing the $^1$H-$^{15}$N TROSY-HSQC experiment adjusting the WATERGATE element,pulse sequence reported by Roche and coworkers.[6] . . . . . S29
S4 Eluent Gradient Conditions for Below HPLC Traces . . . . . S34
S5 Eluent Gradient Conditions for UPLC Trace of **4** and **2-N·OH** to test stability in water. . . . . S37

# 1 General Information

## 1.1 Materials

Unless otherwise specified, all commercial products and reagents were used as purchased, without further purification. Resins for peptide synthesis were purchased through Chem-Impex International,and all solvents were reagent grade. Solvents including, THF, DCM, and DMF were dried *via* a Glass Contours Inc. solvent purification system. Analytical thin-layer chromatography (TLC) and flash chromatography of all reactions was performed on silica gel (200 μm). Flash chromatography of all reactions was performed on silica gel (40-63 $\mu$m, 230-400 mesh) purchased from SiliCycle Inc. Automated flash chromatography of all reactions were performed using columns detailed in the text on a Combi-Flash NextGen 300+ from Teledyne ISCO.

## 1.2 Experimental

*NMR Spectroscopy:* $^{1}$H and $^{13}$C NMR spectra for all small compounds were acquired in deuterated solvents (as indicated) on a Bruker Spectrometer at the field strengths reported in the text. The chemical shift data are reported in units of $\delta$ (ppm) relative to residual solvent. Peptide NMR studies were conducted on a Bruker 700 and 800-MHz spectrometers equipped with Z-shielded gradient triple resonance cryoprobes.

*Liquid Chromatography:* Peptide analysis was accomplished using an Xbridge Prep OBD C18 (5 μm, 4.6x250mm) column on a Waters HPLC system equipped with detection using a Waters 2998 Photodiode Array Detector. All analysis occurred with water and acetonitrile as eluents, solvents contained 0.1% TFA or $NH_4OH$ where specified. The purification of peptides were performed using an Xbridge Prep OBD C18 (5 μm, 19x250mm) column, both on a Waters HPLC Preparative LC system equipped with detection using a Waters 2998 Photodiode Array Detector. All purification of occurred with water and acetonitrile as eluents, solvents contained 0.1% TFA or $NH_4OH$ where specified.

*Mass Spectrometry:* HRMS data was collected on either an Agilent QTOF 6540 MSMS or Agilent LCTOF 6230, both with ESI ionization and TOF detection. Chemical Formulas found from HRMS data were obtained *via* Agilent MassHunter.

*Circular Dichroism (CD):* Circular dichroism traces were acquired using a Jasco J-715 spectrophotometer (Jasco Analytical Instruments, Easton MD). Concentration was determined by the absorbance at 280 nm utilizing a molar extinction coefficient ($\varepsilon_{280}$) of 1490 $cm^{-1}M^{-1}$ with the use of NanoDrop$^{1000}$.[1]

## 2 Synthetic Methods

### 2.1 Synthesis Synthesis of Thioacylating Reagents (Fmoc-$X_{aa}^{(S)}$-Nbt)

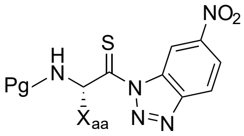

Synthesis of Thioacylating reagents were carried out according to previous procedures with matching spectra.[2,3]

### 2.2 Synthesis of Amidine Dipeptide (6).

#### 2.2.1 Synthesis of Boc-Phe$^{(S)}$Ala-OMe.

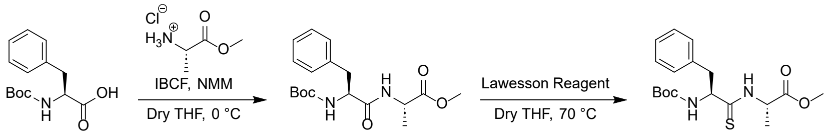

Into an oven dried flask, 1.33 g (5.00 mmol) of Boc-Phe-OH was dissolved in dry THF and placed in an ice bath. To the flask, 1.65 mL (3 eq., 15.0 mmol) of *N*-methyl morphinoline (NMM) was added followed by 0.8 mL of isobutyl chloroformate (1.1 eq, 5.50 mmol). This mixture stirred on ice for ca. 10 min. before 753 mg (1.05 eq, 5.25 mmol) of Ala-OMe hydrochloride salt was added. The resulting was allowed to stir at rt for ca. 4 hours before the precipitate was filtered off. The filtrate was concentrated under reduced pressure, yielding a crude slurry that was re-suspended in ethyl acetate, and washed with 0.1 *M* HCl, sat. $NaH_2CO_3$, and brine (all 2 x 30 mL). The organic layers were dried over sodium sulfate, filtered and concentrated under reduced pressure to yield crude product. The crude dipeptide was re-suspended in minimal THF. While in a separate flask 3.033 g (1.5 eq, 7.50 mmol) Lawesson's reagent was dissolved in 30 mL of THF, and the Boc-Phe-Ala-OMe solution was added. The resulting mixture was allowed to stir at reflux while monitored *via* TLC. Upon completion, the reaction was concentrated under reduced pressure, and re-suspended in ethyl acetate. The crude product was washed with sat. $NaH_2CO_3$ until the water layer became clear (ca. 5 washes). The organic layer was dried over sodium sulfate, filtered, and concentrated under reduced pressure to yield crude thioamide. The crude product was then purified *via* silica gel chromatography to yield 1.503 g (85%, over 2 steps) of an off-white solid. Spectral data matched reported literature.[4]

#### 2.2.2 Synthesis of Ac-Phe$^{(SMe)}$-Ala-NHEt.

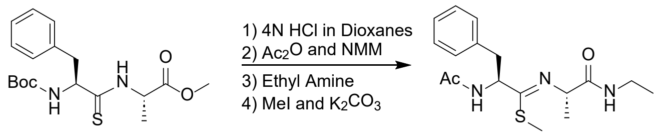

In an oven dried flask 750 mg (2.04 mmol) of Boc-Phe$^{(S)}$-Ala-OMe thioamide dipeptide was dissolved in a minimum amount of 1,4-dioxane. The flask was placed in ice bath before ca. 8.0 mL of 4N HCl was added. The reaction was allowed to stir at room temperature for 1 hour while monitored *via* TLC. Upon completion the reaction was concentrated under reduced pressure to yield a crude free amine, as an oil. The crude oil was re-suspended in THF and excess (ca. 3 mL) NMM was added, resulting in a NMM-HCl salt precipitate. The salt precipitate was removed *via* filtration and filtrate was placed in a round bottom flask and concentrated under reduced pressure. The crude product was re-suspended in THF, and 250 $\mu$L (1 eq, 2.04 mmol) NMM and 400 $\mu$L (2 eq, 4.08 mmol) of acetic anhydride were added. The resulting mixture stirred at room temperature for ca. 1 hour before being quenched with sat.$NH_4Cl$ and product was back extracted with ethyl acetate 3 times. The organic layers were dried over sodium sulfate, filtered and concentrated under reduced pressure. The crude oil was redissolved in ca. 6.0 mL of methanol, and to the flask 6.0 mL of 70% ethyl amine in water was added. After stirring overnight at room temperature the solvent was removed *in vacuo*. The crude oil was dissolved in acetone and the to the flask 850.0 mg (3 eq., 6.13 mmol) of $K_2CO_3$ was added followed by 250$\mu$L (2 eq., 4.05 mmo) of methyl iodide. The reaction stirred overnight at 35°C before the $K_2CO_3$ was removed. The filtrate was concentrated *in vacuo* to yield crude product. The crude product was re-suspended in DCM, loaded on silica to be purified *via* silica gel chromatography to yield 180 mg (26%, over 4 steps) of a yellow oil.

$^1$H NMR ($CDCl_3$, 600 MHz) inseparable rotamers $\delta$ 7.3-7.2 (m, 3 Hs), 7.17 (d, 1H) 7.11 (d, 1H), 6.77 (s, 1H), 6.70 (s, 1H( 5.20 (m, 1H), 4.20 (m, 1H), 3.26-3.13 (m, 4Hs, overlapped), 2.97-2.92 (m, 1H), 2.45 (m, 3H)s, 1.96 (dt, 5Hs, overlapped), 1.21 (d, 3H), 1.08 (m, 3H).

$^{13}$C NMR ($CDCl_3$, 150 MHz) inseparable rotamers $\delta$ 172.98, 169.35, 166.60, 135.94, 129.65, 129.04, 128.35, 128.299, 127.01, 60.53,

60.20, 51.05, 39.79, 34.19,33.97, 23.1, 17.99, 12.84, 14.81, 14.76, 14.19, 14.07 ppm.
HRMS (ESI-TOF) m/z: [M+H]+ Calcd. for $C_{32}H_{34}NO_2S^+$ 496.2305, found 496.2278.

#### 2.2.3 Synthesis of Ac-Phe$^{(NH)}$-Ala-NHEt (6).

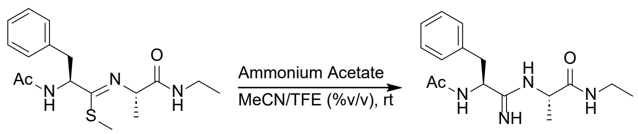


To an oven dried flask, 350 mg (1.05 mmol) Ac-Phe$^{(SMe)}$-Ala-NHEt dipeptide was dissolved in a solvent mixture of 1:1 %v/v TFE/MeCN to yield ca. a 0.1*M* reaction solution. To the mixture 2 eq. of ammonium acetate was added, and the resulting reaction was allowed to stir at room temperature while monitored *via* TLC. Upon completion of the reaction, the reaction concentrated under reduced pressure to yield crude amidine. The crude product was purified *via* reverse phase HPLC with water/MeCN mixture with 0.1% $NH_4OH$ as the eluent. The fractions containing amidine were frozen and lyophilized to yield 310 mg (78%) of an off-white solid.
$^1$H NMR ($D_2O$, 600 MHz) $\delta$ 7.41-7.34 (m, 5H), 4.69 (t, 1H, *J*=12Hz), 4.15 (q, 1H, *J*= 6 Hz), 3.28-3.24 (m, 1H), 3.17-3.14 (m, 2H), 3.12-3.08 (m,1H), 2.02 (s, 3H), 1.39 (d, 3H, *J*= 6Hz), 1.09-1.07 (m, 3H).
$^{13}$C NMR ($D_2O$, 150 MHz) 174.9, 170.2, 166.79, 134.3, 129.0, 128.9, 127.8, 54.2, 51.7, 37.1, 34.7, 21.3, 16.6, 13.3 ppm. HRMS (ESI-TOF) m/z: [M+H]+ Calcd. for $C_{16}H_{25}N_4O_2^+$ 305.1972, found 305.1945.

## 2.3 General Wang Resin Loading

To ensure proper and complete loading, the least amount of solvent was used to ensure concentrated resin loading reactions. To a Chemglass peptide synthesis vessel, Wang resin (1 gram, 1.2 meq/g) was placed and swelled in dry DCM. While in a separate flask the amino acid mixed anhydride was prepared by dissolving Fmoc-Tyr-OH (10 eq, relative to resin) in dry DCM. The flask was placed in an ice bath and *N,N*-Diisopropylcarbodiimide (DIC, 5 eq, relative to resin) was added. The mixture stirred for ca. 20 min before the DCM was removed under reduced pressure to yield crude anhydride, and then re-suspended in dry DMF. After ca. 30 min. the DCM was drained from the peptide vessel, and the resin was washed with dry DMF before the amino acid mixture was added to the vessel. In a small flask 4-(dimethylamino)piperidine (DMAP, 0.1 eq, relative to the resin) was dissolved in a minimal amount of dry DMF and added to the vessel. The resulting mixture was agitated on the rotary mixer, and after 1 hr. the DMF was drained and the resin was washed with DCM. Then resin was then dried overnight to yield Fmoc-*L*-Tyr loaded Wang Resin. The resulting loading of the resin was determined by the absorbance at 278 nm. The dried resin (10 mg) was placed in a 2 mL centrifuge tube with 1 mL of 20 % piperidine solution. The mixture was quickly mixed and then agitated on the rotary mixer for 1 hour. The resin was then allow to settle and 50 $\mu$L of the supernatant was diluted in 5 mL of DMF. The absorbance of the dilution was obtained at 278 nm subtracted from the blank of 20% piperidine. The loading to yield a loading of 1.1 eq/g, was determined from equation:

$$Sj = (1000 * A)/(M * 7800 * D) \tag{1}$$

**S**= substitution of the resin (loading)
**A**= absorbance of the sample
**M**= mass of the resin used (in mg)
**D**= Dilution factor (.01)

## 2.4 General Peptide Synthesis

A Chemglass Peptide Synthesis Vessel was supplied with resin, (Wang resin or Tentagel SRAM Rink Amide) that was swelled in dry DMF for 15 min. with $N_2$ agitation. After swelling, the Fmoc protecting group (and all others going forward) was deprotected with 20% piperidine in dry DMF twice (2 min., then 8 min.). After deprotection of Fmoc, the resin was washed with dry DMF (5 x 1 min.). Coupling of all oxo-amide residues was achieved by dissolving Fmoc-$X_{aa}$-OH (5 eq.) (and all amide residues going forward), HATU (4.9 eq.), and NMM (10 eq.) in dry DMF to a concentration of 100 mM, with respect to the amino acid. This solution was added to the resin and agitated with bubbling $N_2$ for 30 min. After each coupling, the resin was washed with dry DMF (3 x 1 min.).

#### 2.4.1 Thioamide Incorporation

The thioamide residue was introduced by dissolving 2 eq. of thioacylating reagent (Fmoc-$X_{aa}^{(S)}$-Nbt) in dry DCM with DIEA (2 eq.). This solution was agitated with the resin using $N_2$ for 30 min. and repeated two additional times. Following the completion of the third coupling, the resin was washed with dry DCM (3 x 1 min.). The resin was capped with $Ac_2O$ (5 eq.) and NMM (5 eq.) in dry DMF to cap any non-reacted peptide after the addition of the thioamide. The Fmoc group is not removed at this stage.

### 2.4.2 Thioamide Protection

The resin was subjected to a mixture of 0.5 *M* methyl iodide (MeI) and 0.05 *M* DIEA in dry DMF and agitated for 6 hr. on a rotary mixer. Then solvent was drained and a fresh mixture of MeI and DIEA was added.[4] The resin was agitated for an additional 6 hr. on the rotary mixer. Test cleavage was performed to confirm methylation was complete, and upon the presence of thioamide detected *via* MS, additional methylation was required. Upon complete methylation of the thioamide, the Fmoc was deprotected as described above, and subsequent peptide elongation was performed. The final residue was acetyl-capped using $Ac_2O$ (10 eq.) and NMM (5 eq.) in dry DMF.

### 2.4.3 Amidine Formation

After completion of the peptide, the resin was subjected to a a saturated solution of ammonium acetate in DMF/TFE (1:1 %v/v).[5] For isotopic ($^{15}N$) labeling of amidinopeptide, resins were subjected to a saturated $^{15}N$-ammonium acetate in a 1:1 v/v% DMF/TFE. The resin was agitated on a rotary mixer for 24 hours, with fresh reagent being introduced ca. every 8 hours. Test cleavages was performed to confirm full amidine formation *via* HRMS detection. If any presence of thioimidate remained after 24 hr., the resin was further agitated on the rotary mixer, until all thioimidate peptide was reacted.

### 2.4.4 Isolation and Purification

The peptide was cleaved from resin with the cleavage cocktail, 95/2.5/2.5%v/v (TFA:$H_2O$:TIPS). The resin was subjected to the cocktail for 60 minutes. The cocktail was removed into a falcon tube (Tube A), where it was evaporated with a gentle stream of air until a thin film was achieved. To Tube A, cold ether was added, and was centrifuged at 10,000 rpm for 5 min. This was repeated with new cold ether added to Tube A to yield crude peptide as a small pellet. The peptide was dissolved in a solution of MeCN (60%), $H_2O$ (40%), and 0.1% TFA. The peptide was then purified *via* HPLC using solvent gradients indicated below.

# 3 Peptide NMR assignment

Table S1: NMR $^{1}$H, $^{13}$C, and $^{15}$N assignments and chemical shifts ($\delta$ ppm) for peptides **2**, **3** **4** and **5** at 293K, pH 4.5 in $D_2O$/TFE-$d_2$/PBS (5/30/65)

| Residue | $H_\alpha$ | $C_\alpha$ | $C_\beta$ | $^{15}$N[A] | NH | $H_\beta$ | $H_\gamma$ | $H_\delta$ | $C_\gamma$ | $C_\delta$ |
|---|---|---|---|---|---|---|---|---|---|---|
| **2** | | | | | | | | | | |
| Y10 | 4.68 | 57.1 | 38.4 | 120.7 | 7.8 | 3.22, 2.92 | - | - | - | - |
| I9 | 3.96 | 63.2 | 39.2 | 119.8 | 7.9 | 1.67 | 1.57, 1.09, 0.56 | 0.81 | 27.6, 16.3 | 12.6 |
| E8 | 4.30 | 59.3 | 28.6 | - | - | 2.21 | 2.65, 2.55 | - | 32.6 | - |
| A7 | 4.56 | 51.5 | 18.9 | 108.5[B] | 8.02 | 1.68 | - | - | - | - |
| L6 | 4.18 | 57.6 | 41.3 | 119.3 | 8.3 | 1.95, 1.58 | 1.88 | 0.92, 0.93 | 26.6 | 24.6, 22.2 |
| Q5 | 4.11 | 58.8 | 28.2 | 119.0 | 8.03 | 2.27, 2.17 | 2.46, 2.40 | - | 33.9 | - |
| R4 | 4.05 | 58.8 | 29.8 | 117.4 | 7.8 | 1.94, 1.92 | 1.76, 1.65 | 3.23, 3.22 | 27.2 | 43.2 |
| A3 | 4.09 | 54.9 | 17.6 | 122.2 | 7.7 | 1.47 | - | - | - | - |
| V2 | 3.88 | 65.3 | 31.7 | 119.2 | 7.9 | 2.10 | 1.05, 0.96 | - | 21.3, 20.2 | - |
| Q1 | 4.19 | 57.9 | 28.7 | 126.1 | 8.3 | 2.45, 2.14 | 2.46, 2.44 | - | 33.6 | - |
| **3** | $H_\alpha$ | $C_\alpha$ | $C_\beta$ | $^{15}$N[A] | NH | $H_\beta$ | $H_\gamma$ | $H_\delta$ | $C_\gamma$ | $C_\delta$ |
| Y10 | 4.49 | 58.8 | 38.7 | 119.6 | 8.17 | 3.21, 2.87 | - | - | - | - |
| I9 | 3.84 | 63.7 | 39.1 | 119.3 | 7.80 | 1.70 | 1.60, 1.08, 0.47 | 0.81 | 27.8, 16.2 | 12.6 |
| E8 | 4.10 | 58.4 | 28.4 | 117.4 | 7.84 | 2.25, 2.19 | 2.66, 2.50 | - | 33.1 | - |
| A7 | 4.12 | 54.9 | 17.7 | 121.5 | 8.02 | 1.54 | - | - | - | - |
| L6 | 4.18 | 57.8 | 41.6 | 120.5 | 8.32 | 1.94, 1.58 | 1.88, 0.92, 0.94 | - | 26.7 | 24.7, 22.4 |
| Q5 | 4.14 | 57.9 | 28.4 | 119.8 | 8.09 | 2.26, 2.19 | 2.46, 2.44 | - | 33.7 | - |
| R4 | 4.10 | 58.7 | 29.8 | 118.3 | 7.89 | 1.94, 1.93 | 1.77, 1.68 | 3.25, 3.23 | 27.2 | 43.2 |
| A3 | 4.11 | 54.7 | 17.8 | 123.6 | 7.88 | 1.48 | - | - | - | - |
| V2 | 3.91 | 65.1 | 31.9 | 120.5 | 8.04 | 2.11 | 1.05, 0.98 | - | 21.2, 20.3 | - |
| Q1 | 4.22 | 57.8 | 28.8 | 127.2 | 8.40 | 2.14, 2.09 | 2.46, 2.41 | - | 34.05 | - |
| **4** | $H_\alpha$ | $C_\alpha$ | $C_\beta$ | $^{15}$N[A] | NH | $H_\beta$ | $H_\gamma$ | $H_\delta$ | $C_\gamma$ | $C_\delta$ |
| Y10 | 4.66 | 57.48 | 38.14 | 120.3 | 7.9 | 2.18, 2.89 | - | - | - | - |
| I9 | 3.92 | 63.67 | 39.06 | 120.4 | 7.9 | 1.67 | 1.57, 1.10, 0.53 | 0.81 | 27.7, 16.3 | 12.6 |
| E8 | 4.32 | 59.44 | 28.68 | - | - | 2.10, 2.34 | 2.63, 2.53 | - | 32.7 | - |
| A7 | 4.57 | 51.48 | 18.88 | 107.9[B] | 8.05 | 1.69 | - | - | - | - |
| L6 | 4.17 | 57.85 | 41.25 | 119.6 | 8.3 | 1.96, 1.59 | 1.89 | 0.92, 0.93 | 26.6 | 24.6, 22.2 |
| Q5 | 4.11 | 58.88 | 28.13 | 119.5 | 8.03 | 2.18, 2.29 | 2.47, 2.40 | - | 33.9 | |
| R4 | 4.05 | 58.91 | 29.78 | 117.9 | 7.8 | 1.95, 1.92 | 1.76, 1.65 | 3.24, 3.22 | 27.2 | 43.2 |
| A3 | 4.09 | 54.96 | 17.60 | 122.6 | 7.9 | 1.48 | - | - | - | - |
| V2 | 3.88 | 65.30 | 31.65 | 119.7 | 8.9 | 2.11 | 1.06, 0.97 | - | 21.3, 20.2 | - |
| Q1 | 4.22 | 57.90 | 28.68 | 126.1 | 8.3 | 2.14, 2.45 | 2.46, 2.44 | - | 33.6 | - |
| **5** | $H_\alpha$ | $C_\alpha$ | $C_\beta$ | $^{15}$N[A] | NH | $H_\beta$ | $H_\gamma$ | $H_\delta$ | $C_\gamma$ | $C_\delta$ |
| Y10 | 4.57 | 58.0 | 39.3 | 120.7 | 7.84 | 3.16, 2.89 | - | - | - | - |
| I9 | 4.04 | 62.4 | 38.6 | 118.7 | 7.75 | 1.78 | 1.34, 1.08, 0.70 | 0.81 | 27.8, 17.5 | 13.4 |
| E8 | 4.28 | 56.9 | 29.2 | 116.8 | 8.02 | 2.08 | 2.44, 2.40 | - | 34.2 | - |
| A7 | 4.25 | 53.6 | 19.1 | 121.5 | 8.19 | 1.44 | - | - | - | - |
| L6 | 4.30 | 56.6 | 42.8 | 123.1 | 8.38 | 1.73, 1.64 | 1.73 | 0.98, 0.93 | 27.6 | 25.2, 23.9 |
| Q5 | 4.42 | 56.6 | 30.1 | 121.6 | 8.59 | 2.19, 2.04 | 2.42, 2.41 | - | 34.4 | - |
| R4 | 4.49 | 58.5 | 31.5 | 119.7 | 7.74 | 2.03, 2.04 | 2.42, 2.41 | 3.24, 3.21 | 27.4 | 43.9 |
| A3 | 4.61 | 51.9 | 19.7 | 104.5[B] | 8.55 | 1.59 | - | - | - | - |
| V2 | 4.16 | 62.6 | 32.9 | 118.7 | 8.09 | 2.17 | 0.97, 0.96 | - | 34.5 | - |
| Q1 | 4.34 | 56.7 | 29.8 | 125.6 | 8.30 | 2.10, 2.01 | 2.41, 2.39 | - | 21.6, 20.7 | - |

A) chemical shifts of backbone nitrogen B) The shift of $^{15}$N isotopically labeled nitrogen, that was installed from the amidine formation reaction.

Table S2: NMR $^{1}H$, $^{13}C$, and $^{15}N$ assignments and chemical shifts ($\delta$ ppm) for peptides **2**, **4** and **5** at 293K, pH 7.4 in $D_2O$/TFE-$d_2$/PBS (5/30/65)

| Residue | $H_\alpha$ | $C_\alpha$ | $C_\beta$ | $^{15}N^A$ | NH | $H_\beta$ | $H_\gamma$ | $H_\delta$ | $C_\gamma$ | $C_\delta$ |
|---|---|---|---|---|---|---|---|---|---|---|
| **2** | | | | | | | | | | |
| Y10 | 4.51 | 59.2 | 39.7 | 127.2 | 7.64 | 3.15, 2.87 | - | - | - | - |
| I9 | 4.08 | 62.6 | 39.0 | 121.5 | 7.90 | 1.77 | 1.51, 1.12, 0.68 | 0.84 | 27.31, 16.7 | 12.6 |
| E8 | 4.30 | 59.4 | 30.3 | 120.6 | 8.01 | 2.26, 2.22 | 2.43, 2.36 | - | 35.6 | - |
| A7 | 4.60 | 51.4 | 19.6 | 117.9 | 8.15 | 1.66 | - | - | - | - |
| L6 | 4.25 | 58.8 | 41.5 | 120.5 | 8.28 | 1.88, 1.61 | 1.82 | 0.94, 0.93 | 26.7 | 24.6, 22.4 |
| Q5 | 4.18 | 58.1 | 28.5 | 120.5 | 8.22 | 2.20, 2.17 | 2.45, 2.41 | - | 33.9 | - |
| R4 | 4.12 | 58.2 | 29.9 | 119.1 | 7.96 | 1.92, 1.89 | 2.45, 2.41 | 3.24, 3.21 | 27.0 | 43.0 |
| A3 | 4.16 | 54.3 | 17.9 | | 7.70 | 1.46 | - | - | - | - |
| V2 | 3.95 | 64.4 | 32.0 | 120.6 | 8.04 | 2.10 | 1.03, 0.97 | - | 20.9, 20.3 | - |
| Q1 | 4.25 | 57.23 | 28.9 | 127.1 | 8.34 | 2.13, 2.06 | 2.45, 2.41 | - | 33.6 | - |
| **4** | $H_\alpha$ | $C_\alpha$ | $C_\beta$ | $^{15}N^A$ | NH | $H_\beta$ | $H_\gamma$ | $H_\delta$ | $C_\gamma$ | $C_\delta$ |
| Y10 | 4.68 | 57.4 | 38.2 | 123.7 | 7.54 | 3.19, 2.92 | - | - | - | - |
| I9 | 3.99 | 63.2 | 38.9 | 120.6 | 8.05 | 1.71 | 1.55, 1.12, 0.59 | 0.83 | 27.6, 16.4 | 12.6 |
| E8 | 4.32 | 59.6 | 30.0 | | | 2.27, 2.29 | 2.41, 2.37 | - | 35.5 | |
| A7 | 4.58 | 51.5 | 19.1 | | | 1.69 | - | - | - | - |
| L6 | 4.25 | 57.6 | 41.4 | 127.2 | 8.42 | 1.94, 1.61 | 1.87 | 0.93, 0.93 | 26.7 | 24.6, 22.3 |
| Q5 | 4.17 | 58.5 | 28.3 | 117.2 | 8.26 | 2.26, 2.19 | 2.48, 2.42 | - | 33.9 | - |
| R4 | 4.11 | 58.5 | 29.8 | 121.6 | 8.19 | 1.96, 1.93 | 1.75, 1.69 | 3.26, 3.23 | 27.1 | 43.1 |
| A3 | 4.14 | 54.7 | 17.8 | 120.5 | 8.23 | 1.48 | - | - | - | - |
| V2 | 3.92 | 64.8 | 31.8 | 121.6 | 8.06 | 2.12 | 2.46, 2.43 | - | 21.1, 20.3 | - |
| Q1 | 4.22 | 57.4 | 28.8 | 127.2 | 8.44 | 2.15, 2.09 | 2.46, 2.43 | - | 33.6 | - |
| **5** | $H_\alpha$ | $C_\alpha$ | $C_\beta$ | $^{15}N^A$ | NH | $H_\beta$ | $H_\gamma$ | $H_\delta$ | $C_\gamma$ | $C_\delta$ |
| Y10 | 4.54 | 57.9 | 38.8 | 121.3 | 7.83 | 3.18, 2.84 | - | - | - | - |
| I9 | 3.96 | 61.9 | 38.7 | 119.7 | 7.69 | 1.77 | 1.34, 1.05, 0.63 | 0.78 | 27.2, 16.7 | - |
| E8 | 4.17 | 57.4 | 30.2 | 117.6 | 7.96 | 2.29, 2.15 | 2.31, 2.29 | - | 36.9 | - |
| A7 | 4.18 | 53.7 | 18.6 | 121.1 | 7.64 | - | - | - | - | - |
| L6 | 4.26 | 56.2 | 41.9 | 124.8 | 8.58 | 1.74, 1.65 | 1.74 | 0.98, 0.94 | 26.9 | 24.4, 23.4 |
| Q5 | 4.51 | 55.3 | 30.3 | - | - | 2.11, 1.98 | - | - | - | - |
| R4 | 4.98 | 58.0 | 31.3 | - | - | 1.97, 1.98 | 1.67, 1.62 | 3.19, 3.18 | 26.6 | 43.3 |
| A3 | 4.58 | 51.6 | 19.7 | - | - | 1.56 | - | - | - | - |
| V2 | 4.18 | 61.9 | 32.7 | 126.8 | 8.10 | 2.18 | 0.95, 0.94 | - | 20.9, 19.9 | - |
| Q1 | 4.34 | 56.0 | 29.4 | 120.2 | 8.30 | 2.05, 1.98 | 2.40, 2.38 | - | 33.7 | - |

A) chemical shifts of backbone nitrogen B) The shift of $^{15}N$ isotopically labeled nitrogen, that was installed from the amidine formation reaction.

# 4 NMR Spectra

## 4.1 Dipeptide NMR spectra

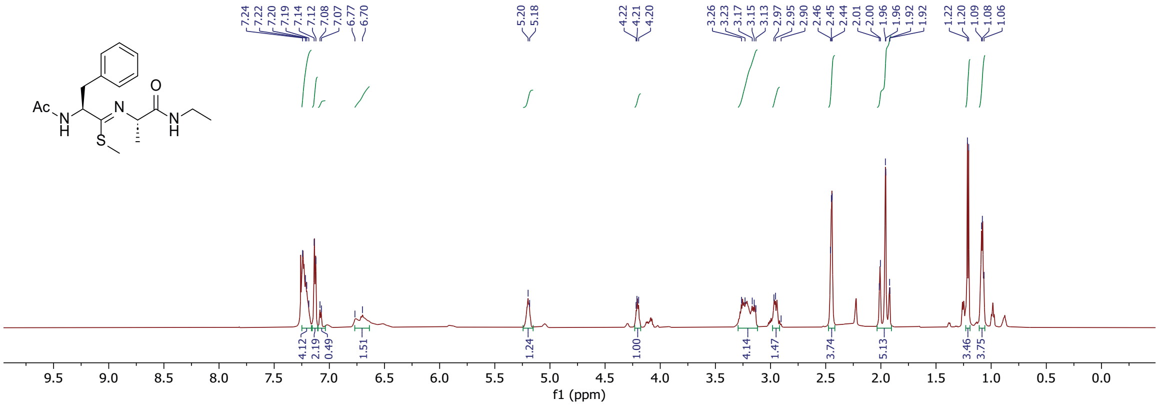


Figure S1: 600 MHz $^{1}H$ NMR spectra Ac-Phe$^{(SMe)}$-Ala-NHEt in $CDCl_3$

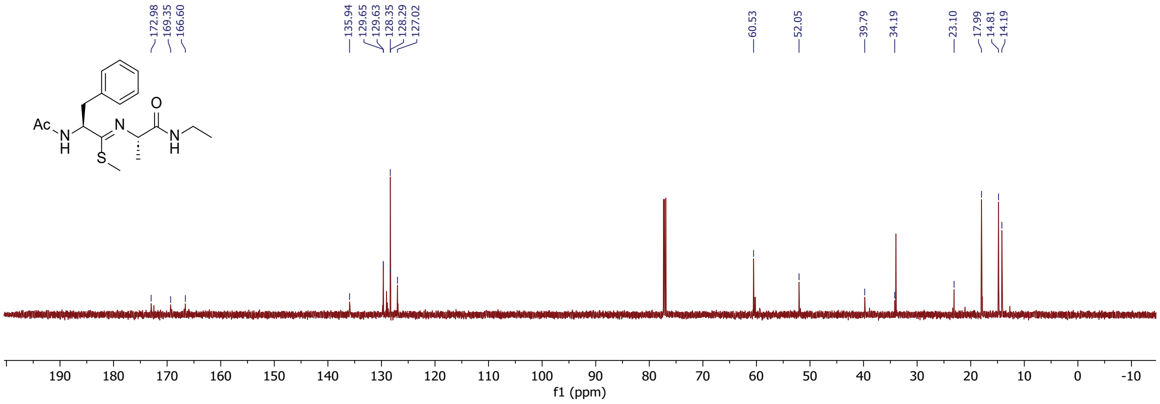


Figure S2: 150 MHz $^{13}C$ NMR spectra Ac-Phe$^{(SMe)}$-Ala-NHEt in $CDCl_3$

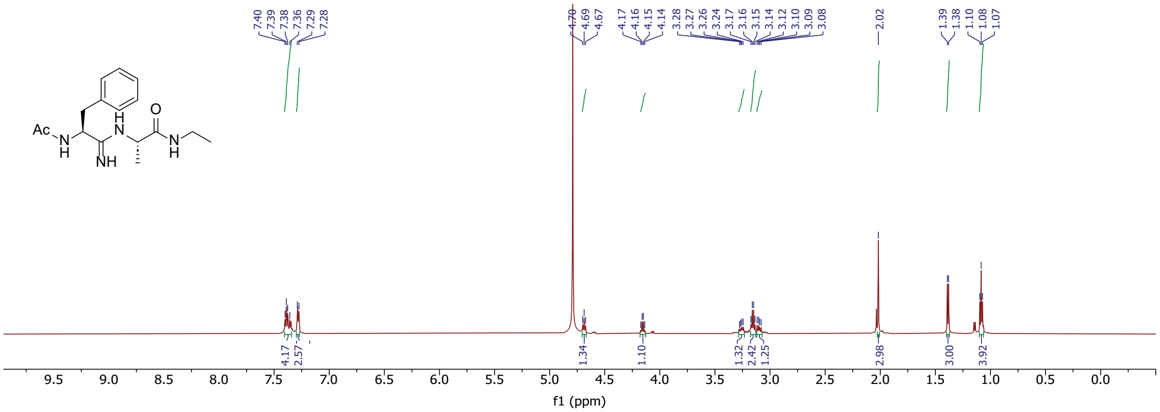

Figure S3: 600 MHz $^{1}$H NMR spectra Ac-Phe$^{(NH)}$-Ala-NHEt (**6**) in $D_2O$

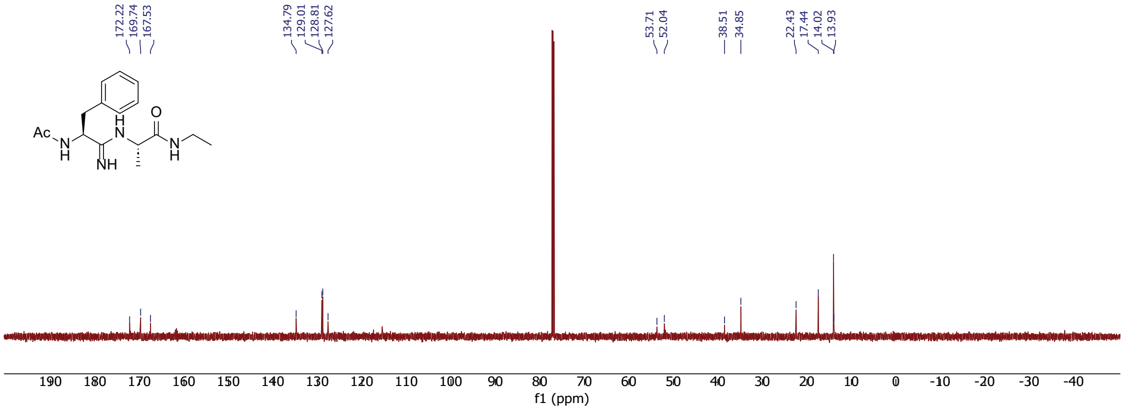

Figure S4: 150 MHz $^{13}$C NMR spectra Ac-Phe$^{(NH)}$-Ala-NHEt **(6)** in $CDCl_3$ for referencing

## 4.2 Peptide NMR data

### 4.2.1 Peptide NMR parameters

Amidinopeptides were assigned utilizing NMR experiments: TOCSY, NOESY, $^{13}$C-H HSQC, $^{15}$N-H HSQC. The lyophilized peptides were dissolved in 450 $\mu$L of a $D_2O$/TFE/PBS Buffer mixture (5/30/65 %v/v) at the desired pH (4.5/7.4), adjusted with the use of 0.2M HCl. Each sample's concentration was determined by the UV absorption of Tyr residue at 280 ($\varepsilon$=1490) to yield NMR sample concentrations around 1 to 1.5 mM. NMR spectra were recorded on a Bruker Avance II 700 and 800 MHz spectrometers both equipped with Z-shielded gradient triple resonance cryoprobes.. The NMR spectra were obtained at a temperature of 20°C, unless noted otherwise. All samples were ran before the inclusion of Sodium trimethylsilylpropanesulfonate (DSS), the DSS was used to reference the chemical shifts for characterization of peptides.

**NOESY** ns (32), TD (320), and mixing time 150-400 ms
**TOCSY** ns (64), TD (256), and mixing time of 80 ms
**$^{13}$C-HSQC** ns (32), TD (256)
**$^{15}$N-HSQC** ns (32), TD (120)

### 4.2.2 NMR spectra of Amidinopeptides at pH 4.5

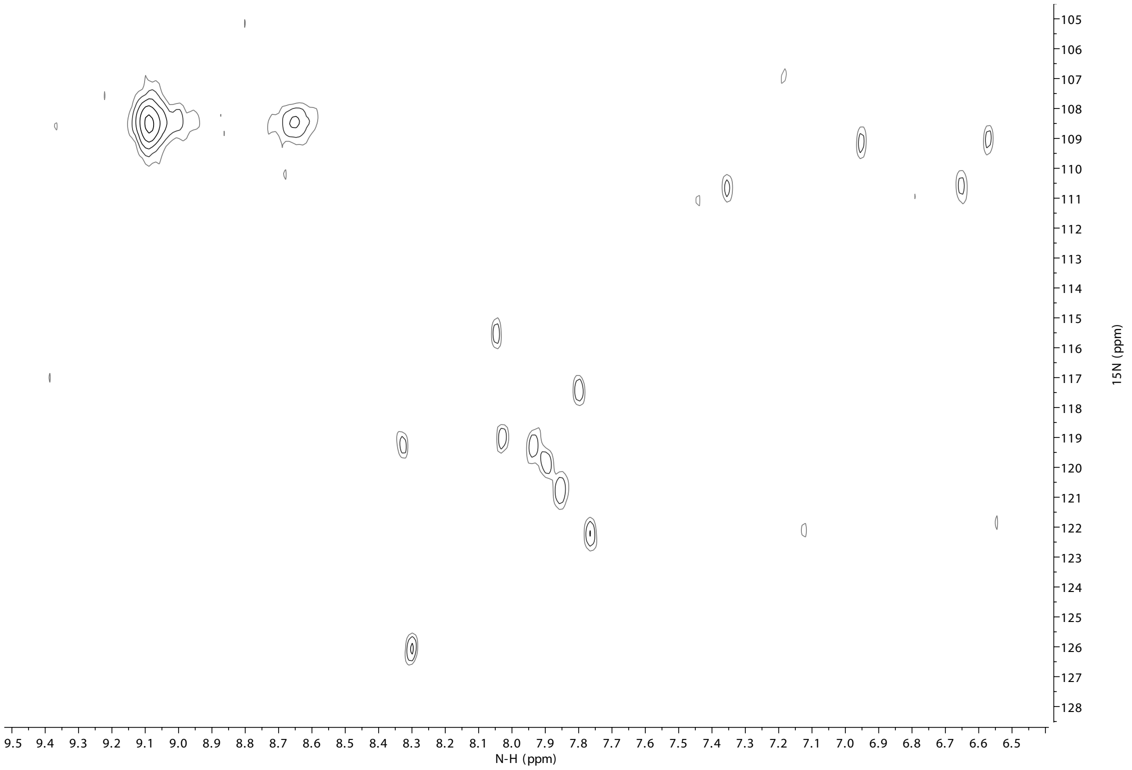


Figure S5: 700 MHz $^{15}$N-H HSQC spectrum (293K) of AcQVARQLA$^{NH}$EIY-$CO_2$, Peptide **2** in $D_2O$/TFE-$d_2$/PBS (5/30/65%v/v) at pH 4.5

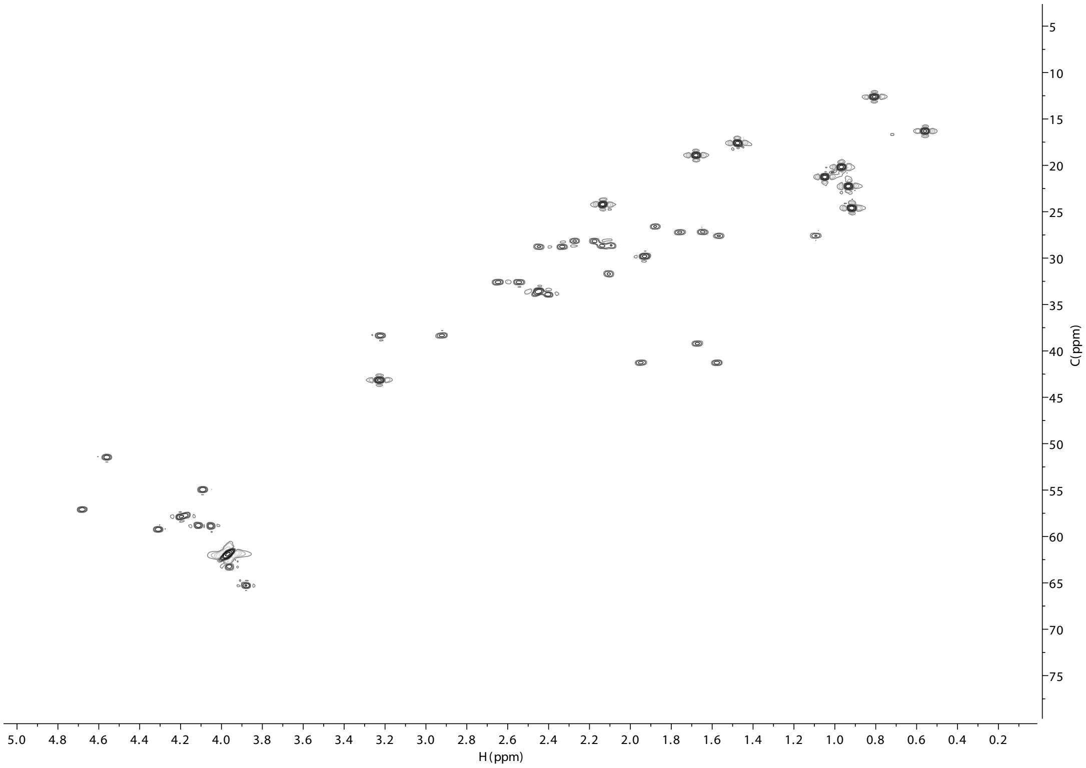


Figure S6: 700 MHz $^{13}$C-H HSQC spectrum (293K) of AcQVARQLA$^{NH}$EIY-$CO_2$, Peptide **2** in $D_2O$/TFE-$d_2$/PBS (5/30/65%v/v) at pH 4.5

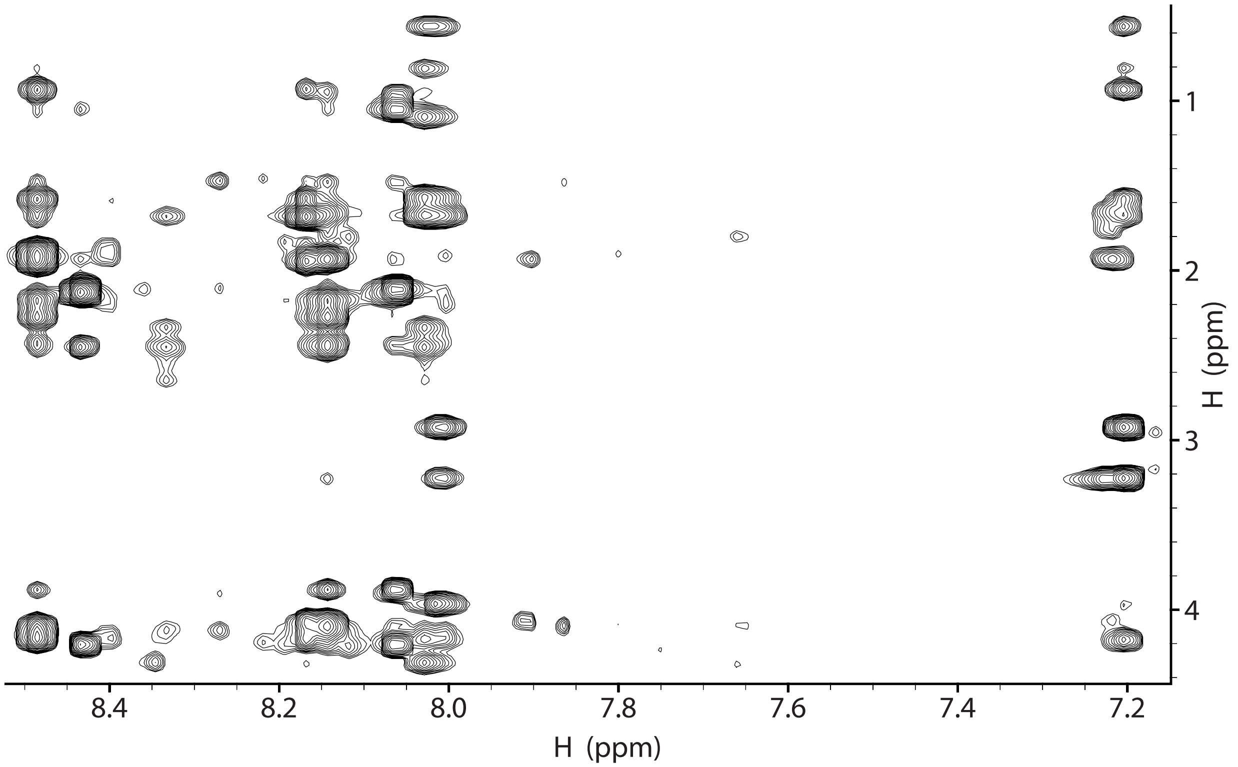


Figure S7: 700 MHz finger print region of NOESY spectrum (293K) of AcQVARQLA$^{NH}$EIY-$CO_2$, Peptide **2** in $D_2O$/TFE-$d_2$/PBS (5/30/65%v/v) at pH 4.5

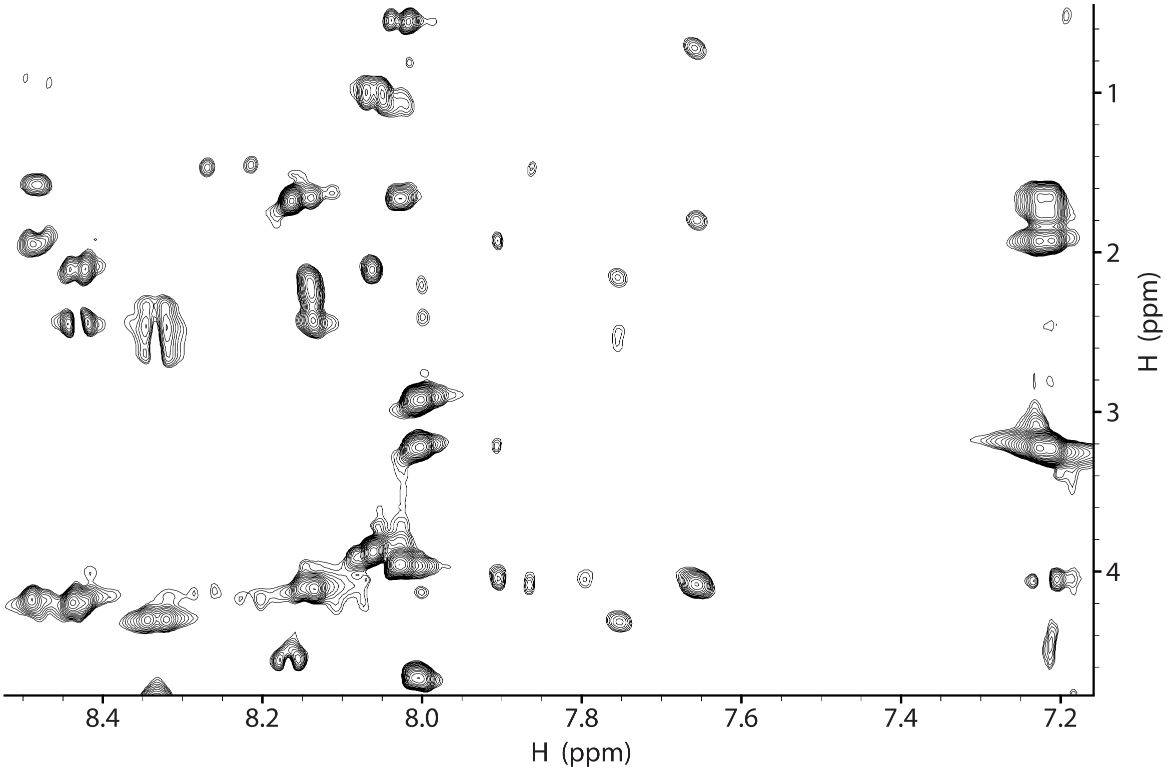


Figure S8: 700 MHz finger print region of TOCSY spectrum (293K) of AcQVARQLA$^{NH}$EIY-$CO_2$, Peptide **2** in $D_2O$/TFE-$d_2$/PBS (5/30/65%v/v) at pH 4.5

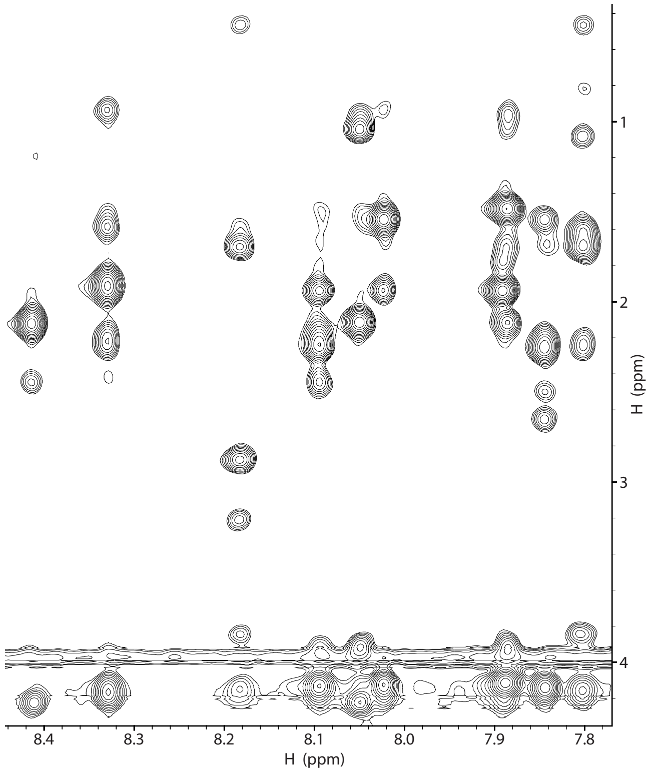


Figure S9: 800 MHz finger print region of NOESY spectrum (293K) of AcQVARQLAEIY-$NH_2$, Peptide **3** in $D_2O$/TFE-$d_2$/PBS (5/30/65%v/v) at pH 4.5

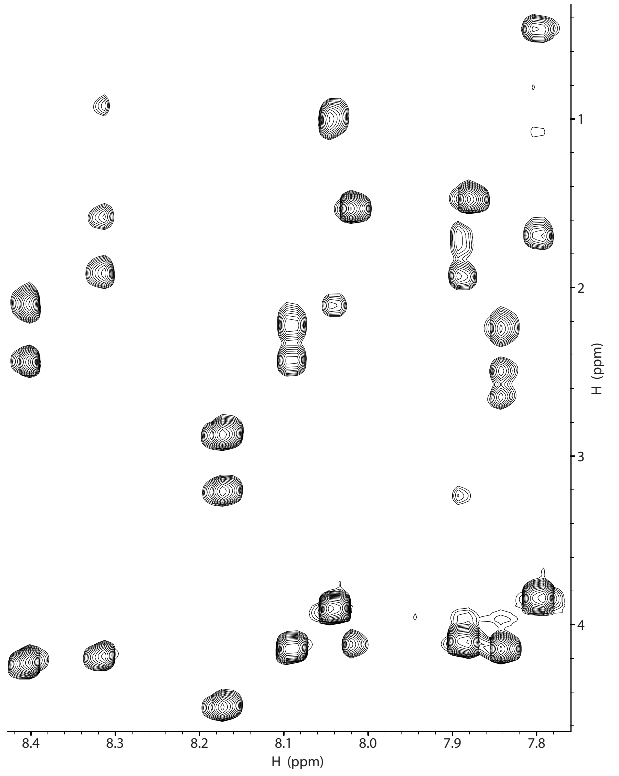


Figure S10: 800 MHz finger print region of TOCSY spectrum (293K) of AcQVARQLAEIY-$NH_2$, Peptide **3** in $D_2O$/TFE-$d_2$/PBS (5/30/65%v/v) at pH 4.5

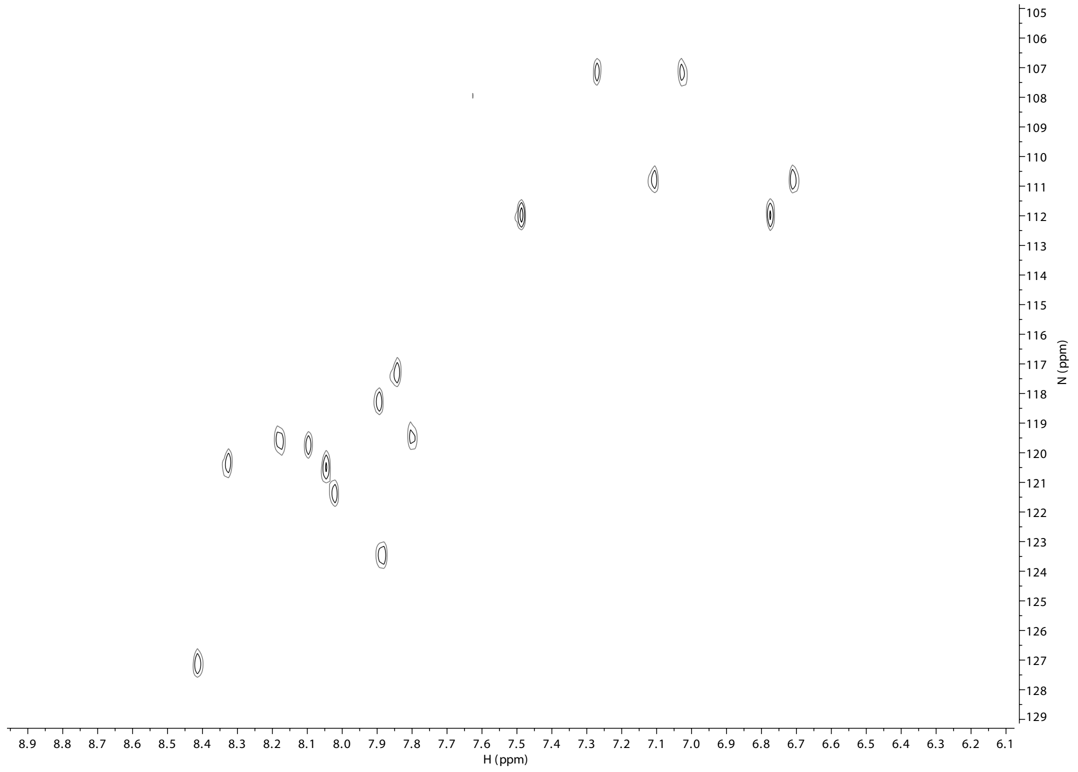


Figure S11: 800 MHz $^{15}$N-H HSQC spectrum (293K) of AcQVARQLAEIY-$NH_2$, Peptide **3** in $D_2O$/TFE-$d_2$/PBS (5/30/65%v/v) at pH 4.5

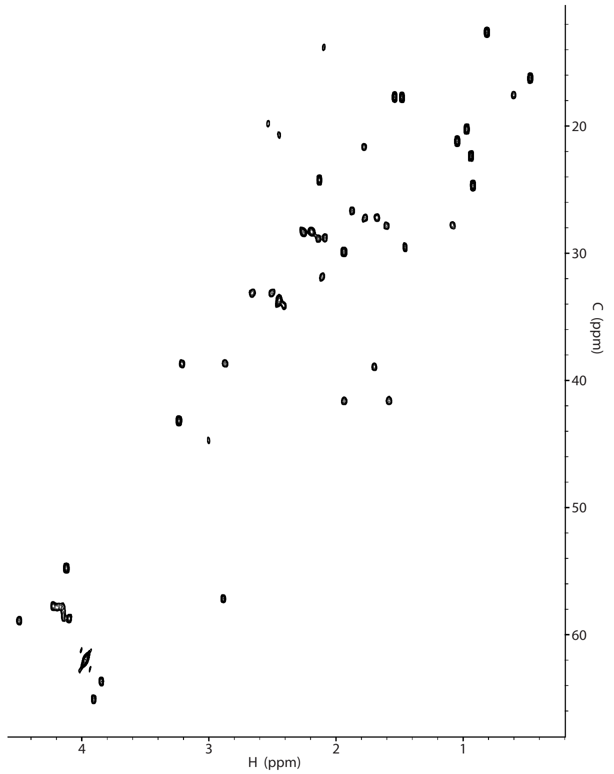


Figure S12: 800 MHz $^{13}$C-H HSQC spectrum (293K) of AcQVARQLAEIY-$NH_2$, Peptide **3** in $D_2O$/TFE-$d_2$/PBS (5/30/65%v/v) at pH 4.5

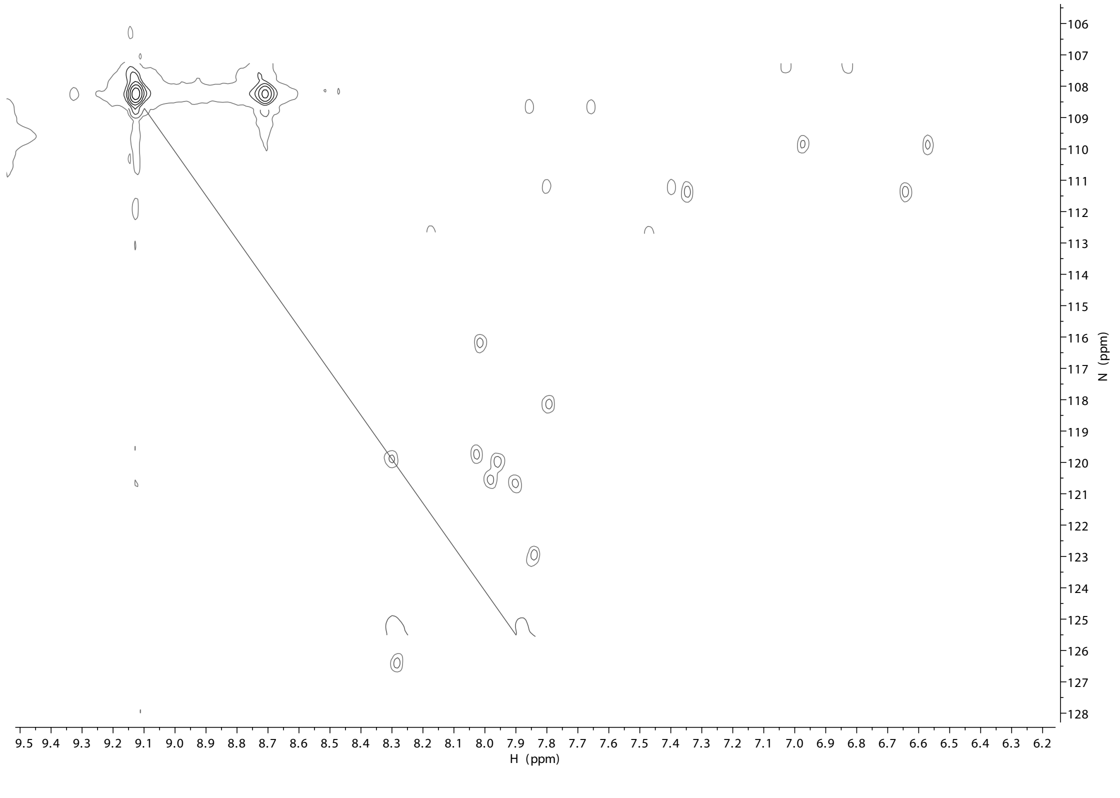


Figure S13: 700 MHz $^{15}$N-H HSQC spectrum (293K) of AcQVARQLA$^{NH}$EIY-$NH_2$, Peptide **4** in $D_2O$/TFE-$d_2$/PBS (5/30/65%v/v) at pH 4.5

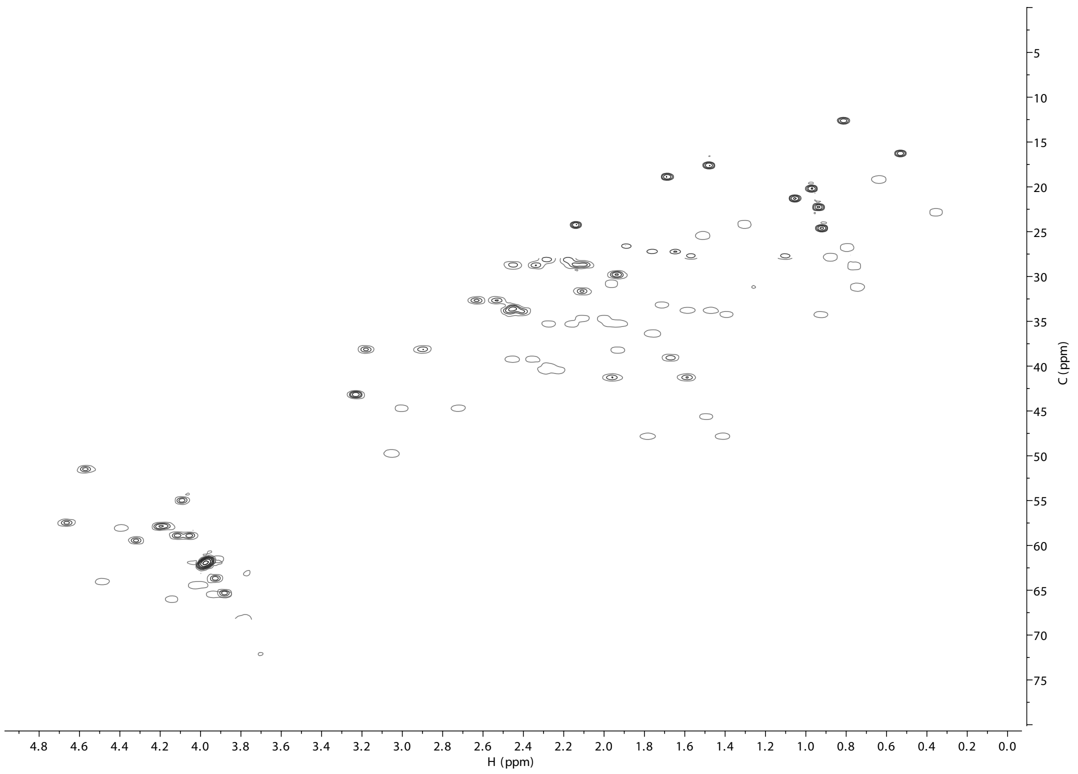


Figure S14: 700 MHz $^{13}$C-H HSQC spectrum (293K) of AcQVARQLA$^{NH}$EIY-NH$_2$, Peptide **4** in D$_2$O/TFE-d$_2$/PBS (5/30/65%v/v) at pH 4.5

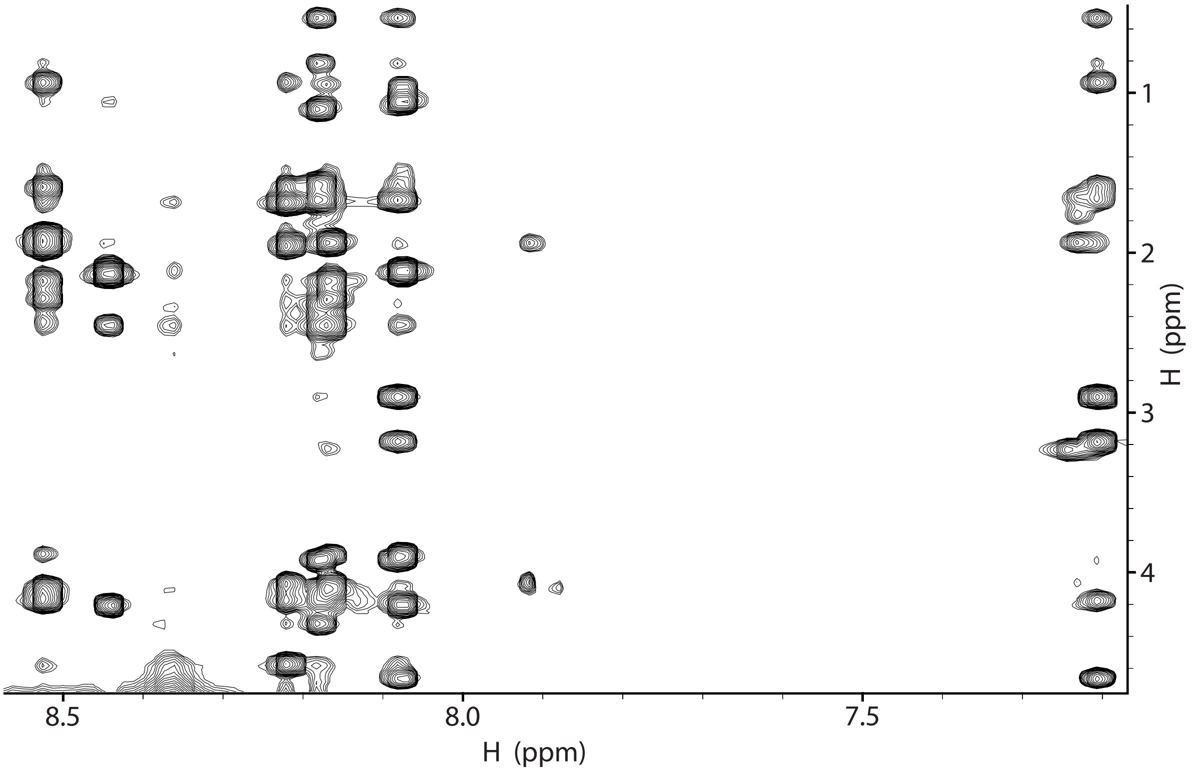


Figure S15: 700 MHz finger print region of NOESY spectrum (293K) of AcQVARQLA$^{NH}$EIY-NH$_2$, Peptide **4** in D$_2$O/TFE-d$_2$/PBS (5/30/65%v/v) at pH 4.5

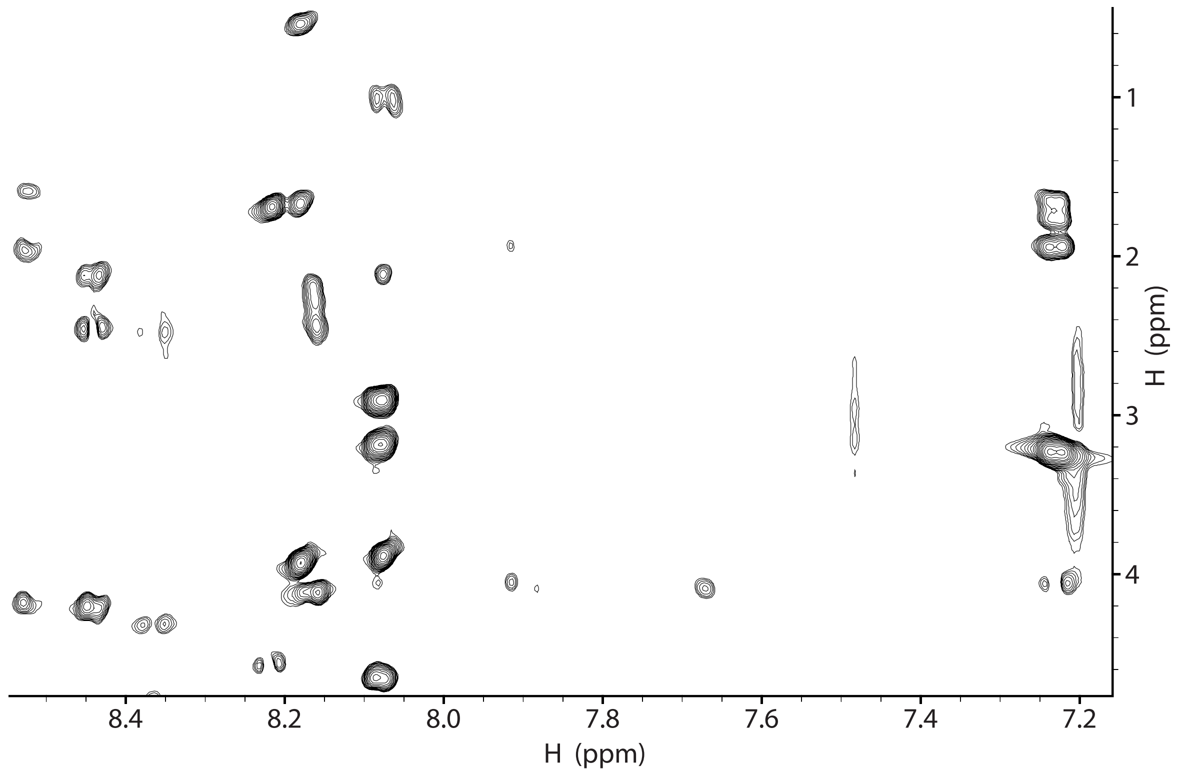


Figure S16: 700 MHz finger print region of TOCSY spectrum (293K) of AcQVARQLA$^{NH}$EIY-$NH_2$, Peptide **4** in $D_2O$/TFE-$d_2$/PBS (5/30/65%v/v) at pH 4.5

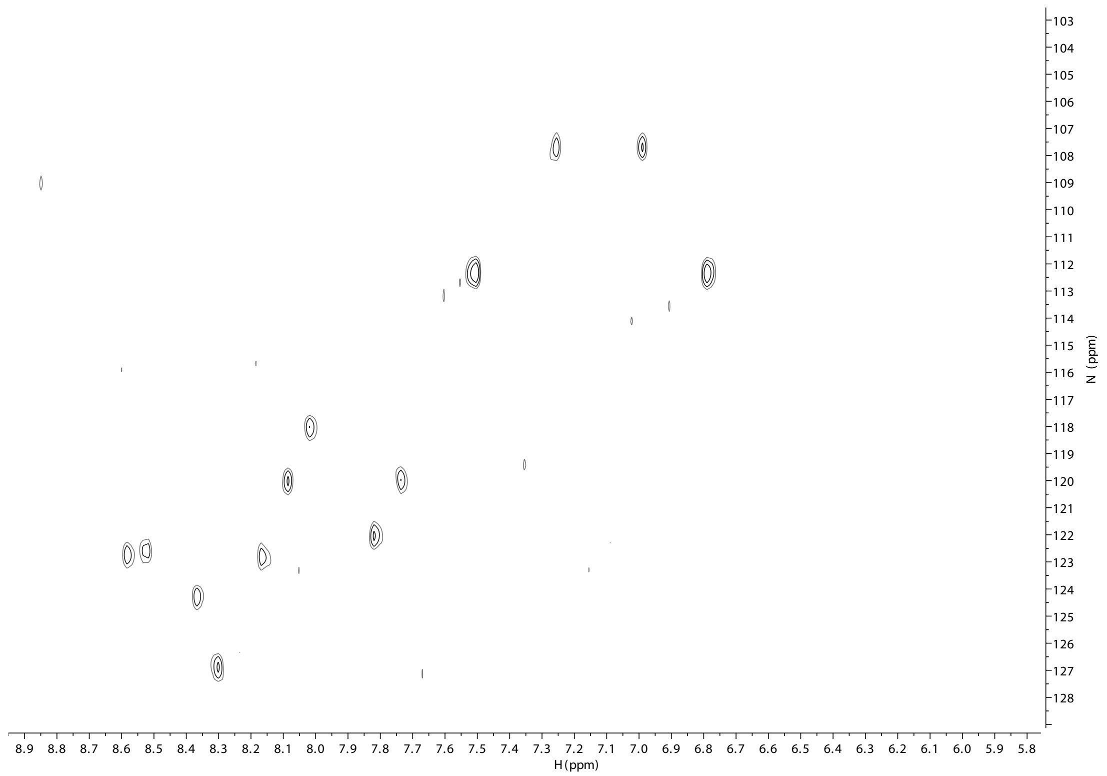


Figure S17: 700 MHz $^{15}$N-H HSQC spectrum (293K) of AcQVA$^{NH}$RQLAEIY-$NH_2$, Peptide **5** in $D_2O$/TFE-$d_2$/PBS (5/30/65%v/v) at pH 4.5

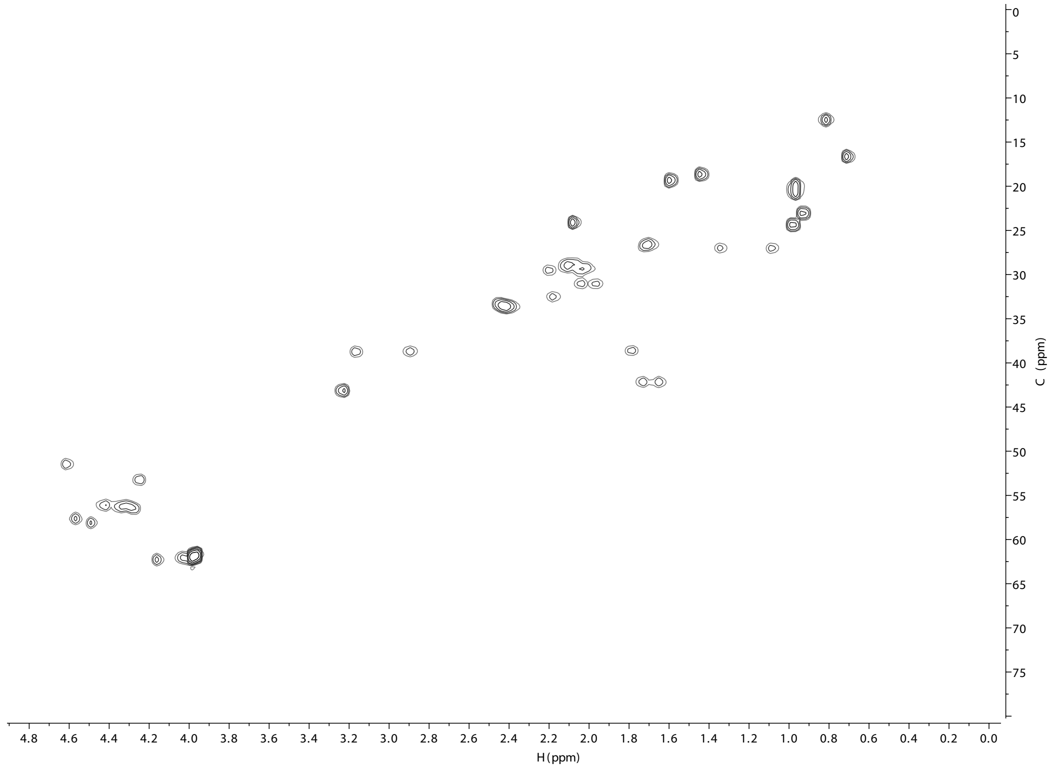


Figure S18: 700 MHz $^{13}$C-H HSQC spectrum (293K) of AcQVA$^{NH}$RQLAEIY-NH$_2$, Peptide **5** in $D_2O$/TFE-$d_2$/PBS (5/30/65%v/v) at pH 4.5

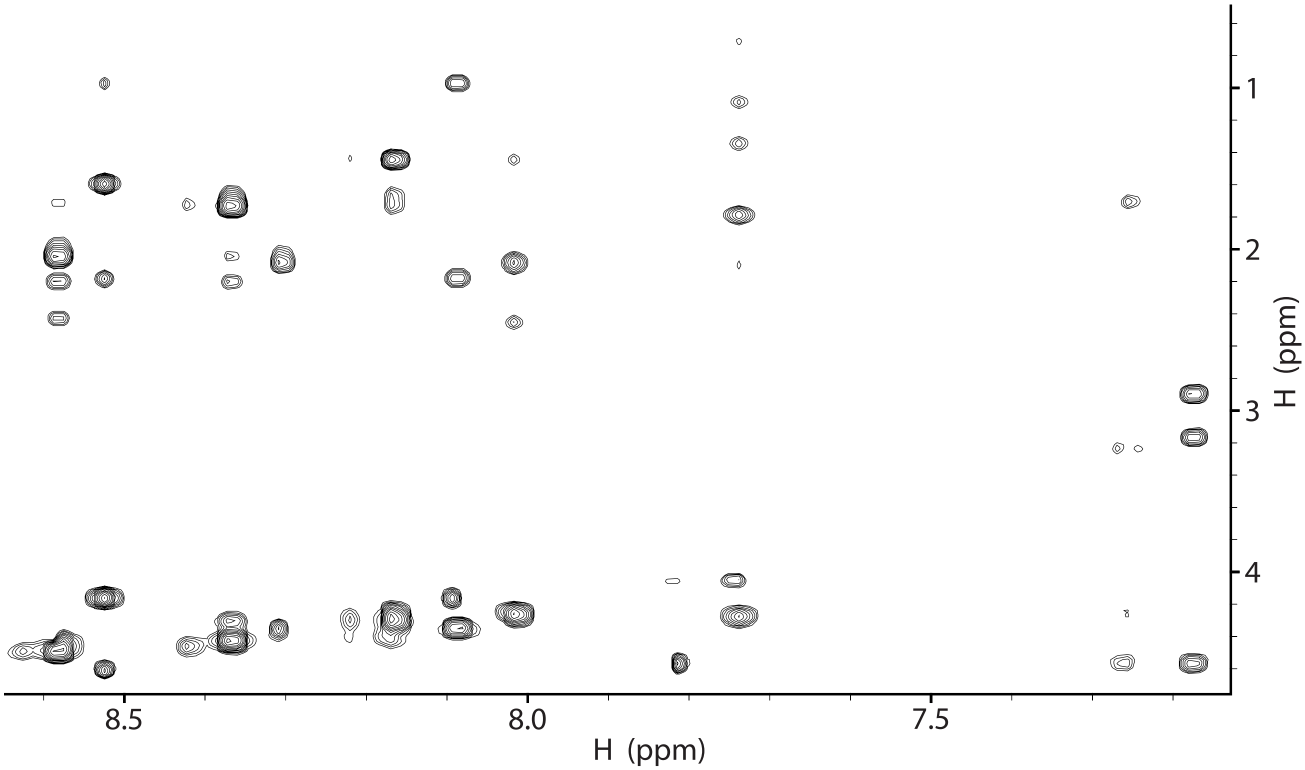


Figure S19: 700 MHz finger print region of NOESY spectrum (293K) of AcQVA$^{NH}$RQLAEIY-NH$_2$, Peptide **5** in $D_2O$/TFE-$d_2$/PBS (5/30/65%v/v) at pH 4.5

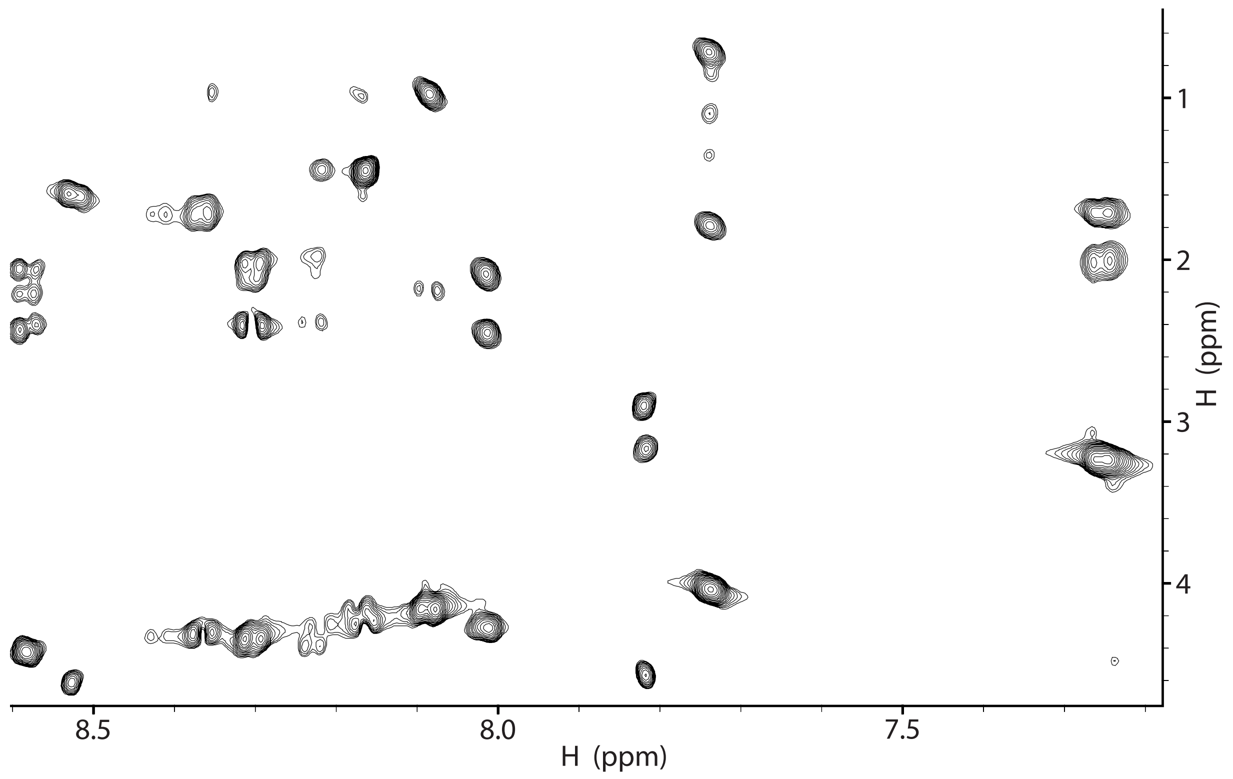


Figure S20: 700 MHz finger print region of TOCSY spectrum (293K) of AcQVA$^{NH}$RQLAEIY-$NH_2$, Peptide **5** in $D_2O$/TFE-$d_2$/PBS (5/30/65%v/v) at pH 4.5

### 4.2.3 NMR spectra of Amidinopeptides at pH 7.4

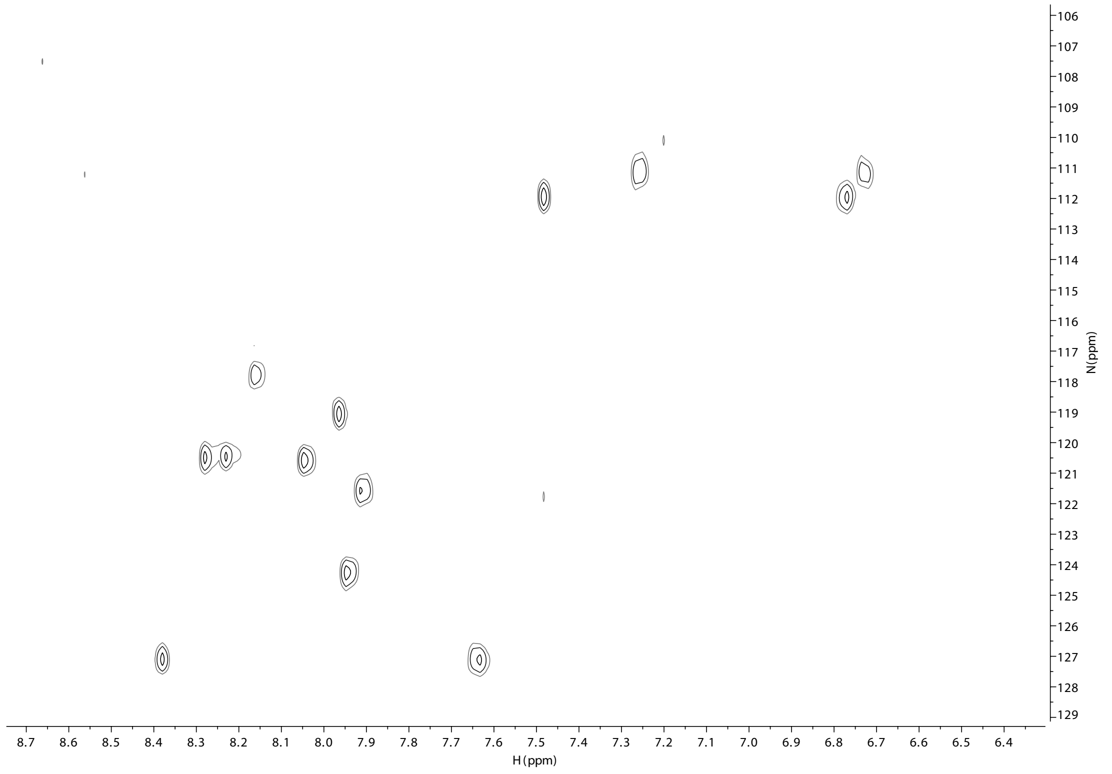


Figure S21: 700 MHz $^{15}N$-H HSQC spectrum (293K) of AcQVARQLA$^{NH}$EIY-$CO_2$ Peptide **2** in $D_2O$/TFE-$d_2$/PBS (5/30/65%v/v) at pH 7.4

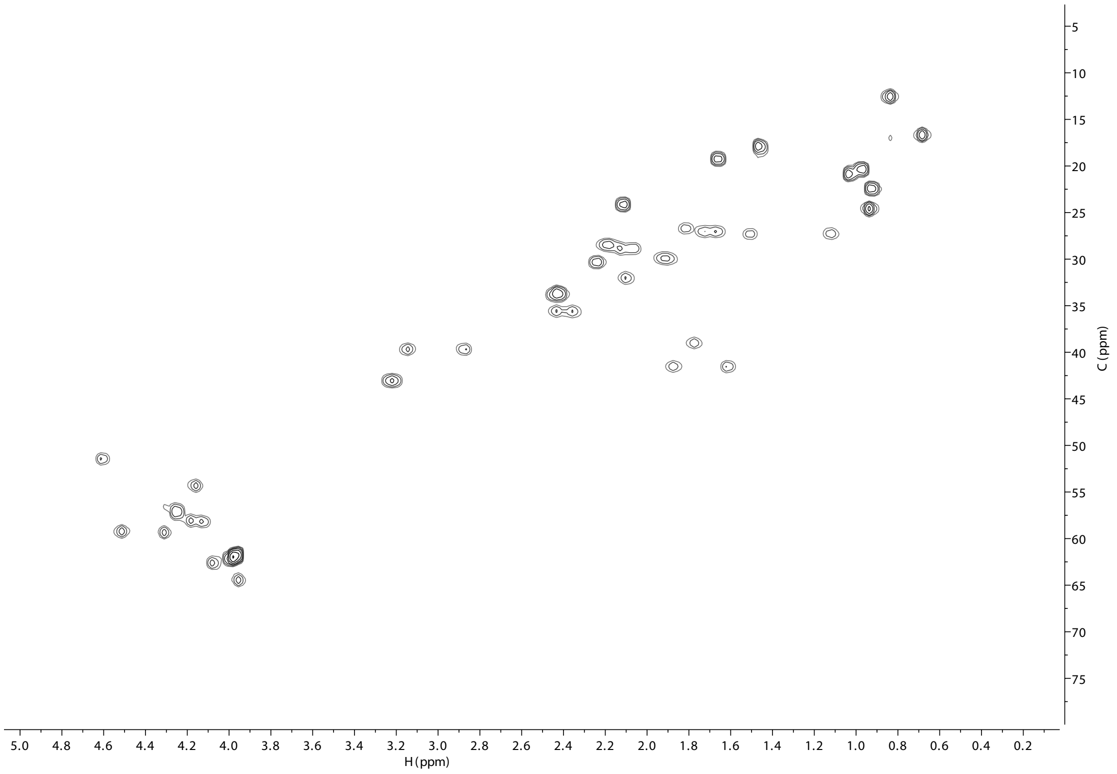


Figure S22: 700 MHz $^{13}C$-H HSQC spectrum (293K) of AcQVARQLA$^{NH}$EIY-$CO_2$, Peptide **2** in $D_2O$/TFE-$d_2$/PBS (5/30/65%v/v) at pH 7.4

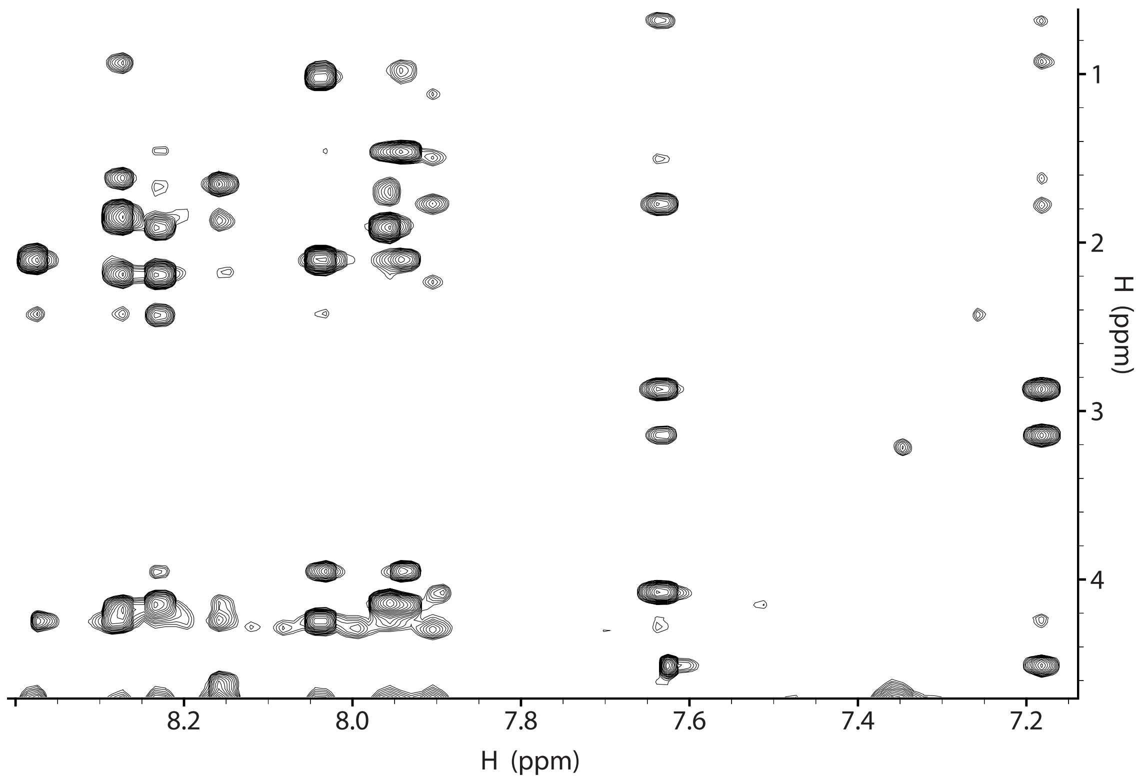


Figure S23: 700 MHz finger print region of NOESY spectrum (293K) of AcQVARQLA$^{NH}$EIY-$CO_2$, Peptide **2** in $D_2O$/TFE-$d_2$/PBS (5/30/65%v/v) at pH 7.4

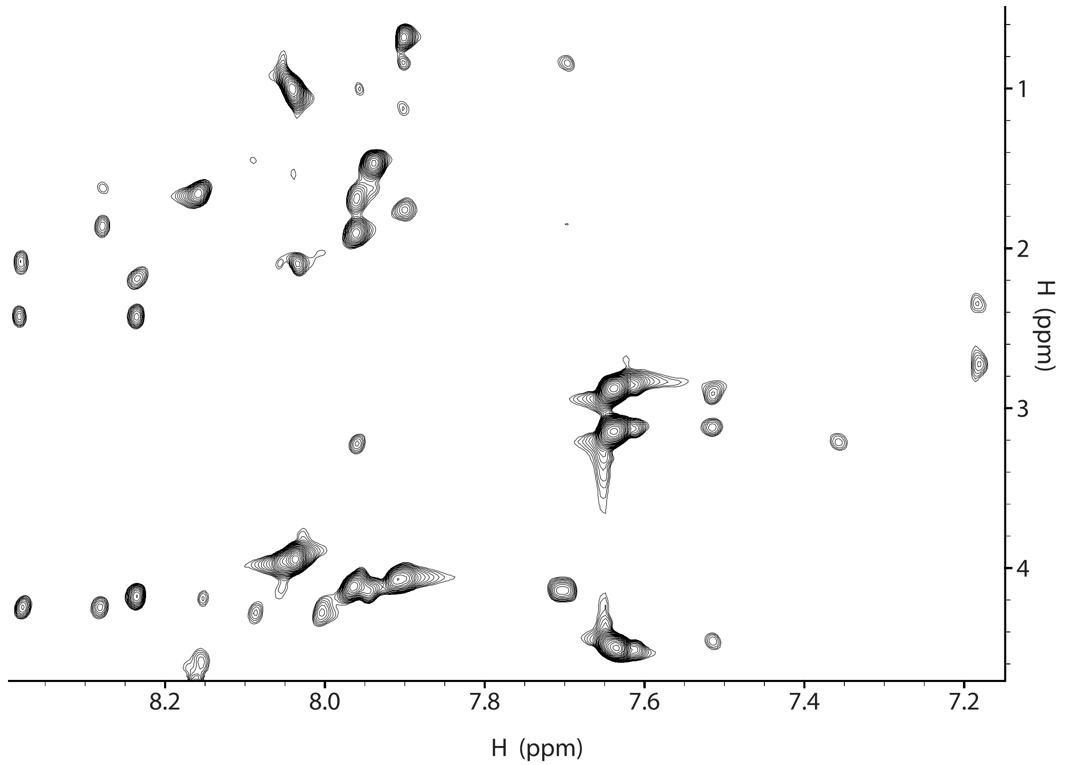


Figure S24: 700 MHz finger print region of TOCSY spectrum (293K) of AcQVARQLA$^{NH}$EIY-$CO_2$ Peptide **2** in $D_2O$/TFE-$d_2$/PBS (5/30/65%v/v) at pH 7.4

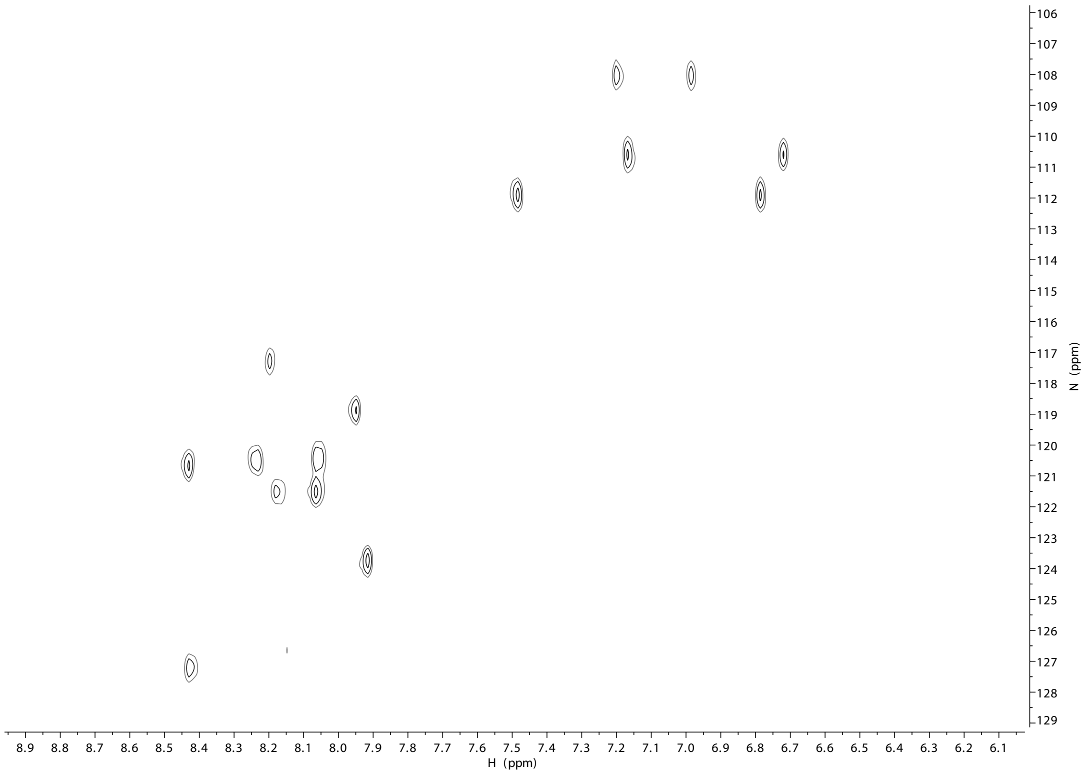


Figure S25: 700 MHz $^{15}N$-H HSQC spectrum (293K) of AcQVARQLA$^{NH}$EIY-$NH_2$, Peptide **4** in $D_2O$/TFE-$d_2$/PBS (5/30/65%v/v) at pH 7.4

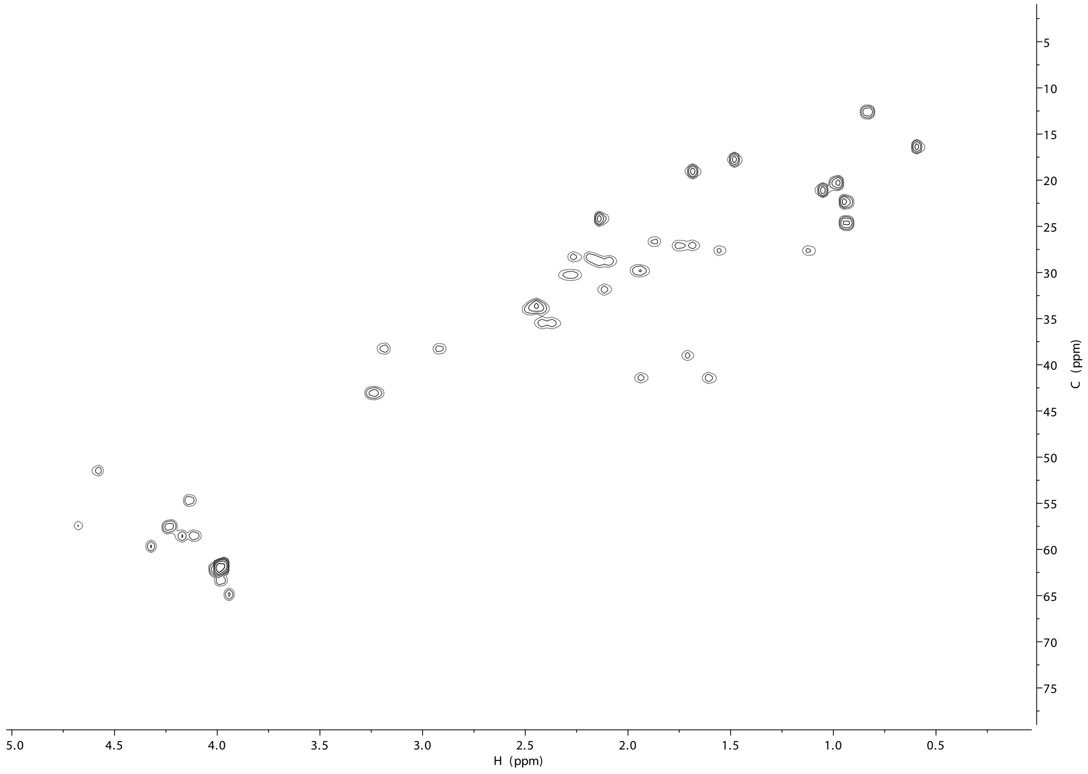


Figure S26: 700 MHz $^{13}C$-H HSQC spectrum (293K) of AcQVARQLA$^{NH}$EIY-$NH_2$, Peptide **4** in $D_2O$/TFE-$d_2$/PBS (5/30/65%v/v) at pH 7.4

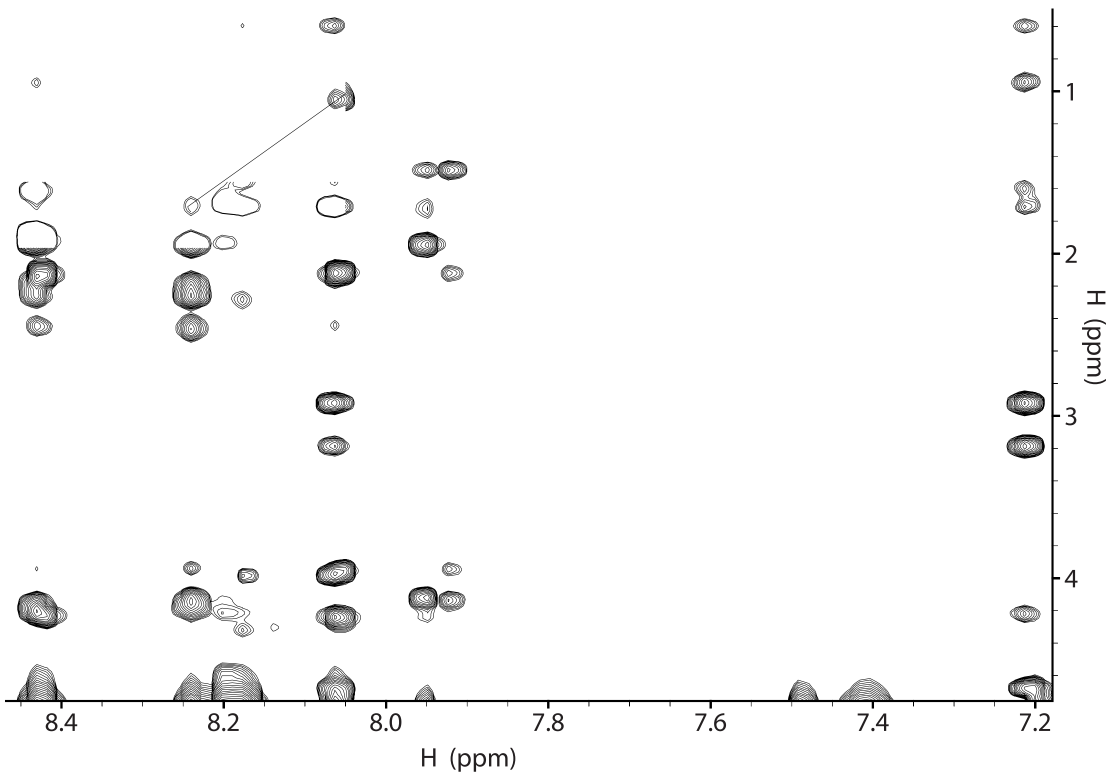


Figure S27: 700 MHz finger print region of NOESY spectrum (293K) of AcQVARQLA$^{NH}$EIY-$NH_2$, Peptide **4** in $D_2O$/TFE-$d_2$/PBS (5/30/65%v/v) at pH 7.4

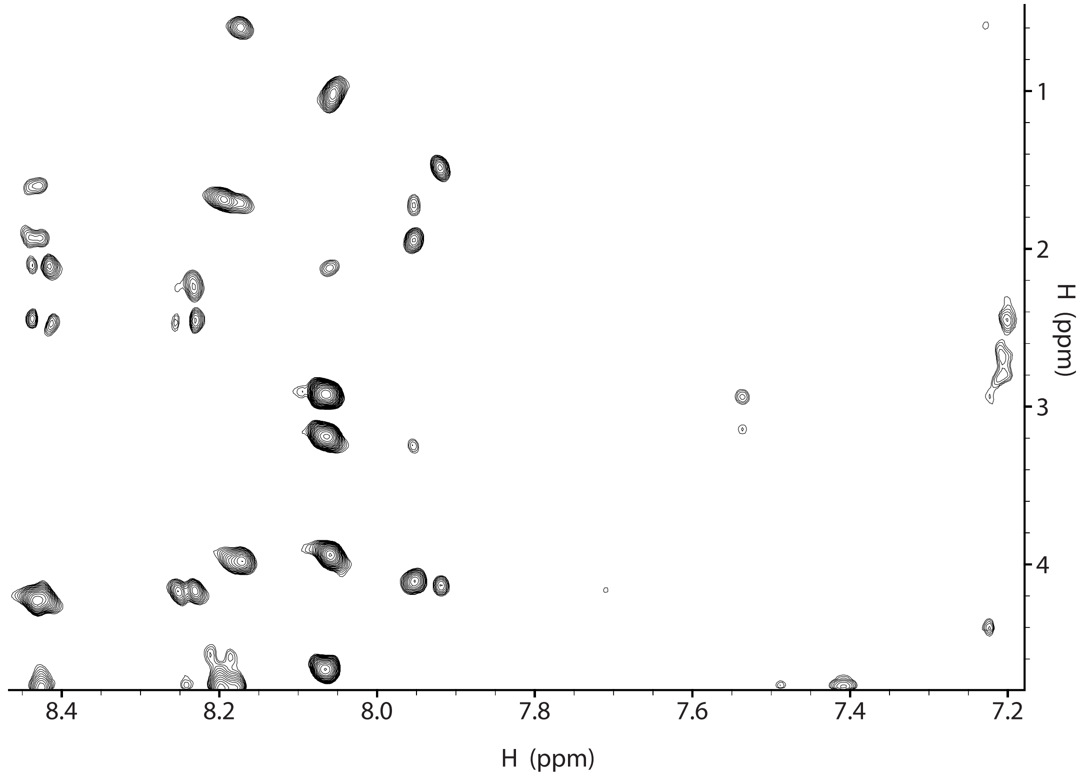


Figure S28: 700 MHz finger print region of TOCSY spectrum (293K) of AcQVARQLA$^{NH}$EIY-$NH_2$, Peptide **4** in $D_2O$/TFE-$d_2$/PBS (5/30/65%v/v) at pH 7.4

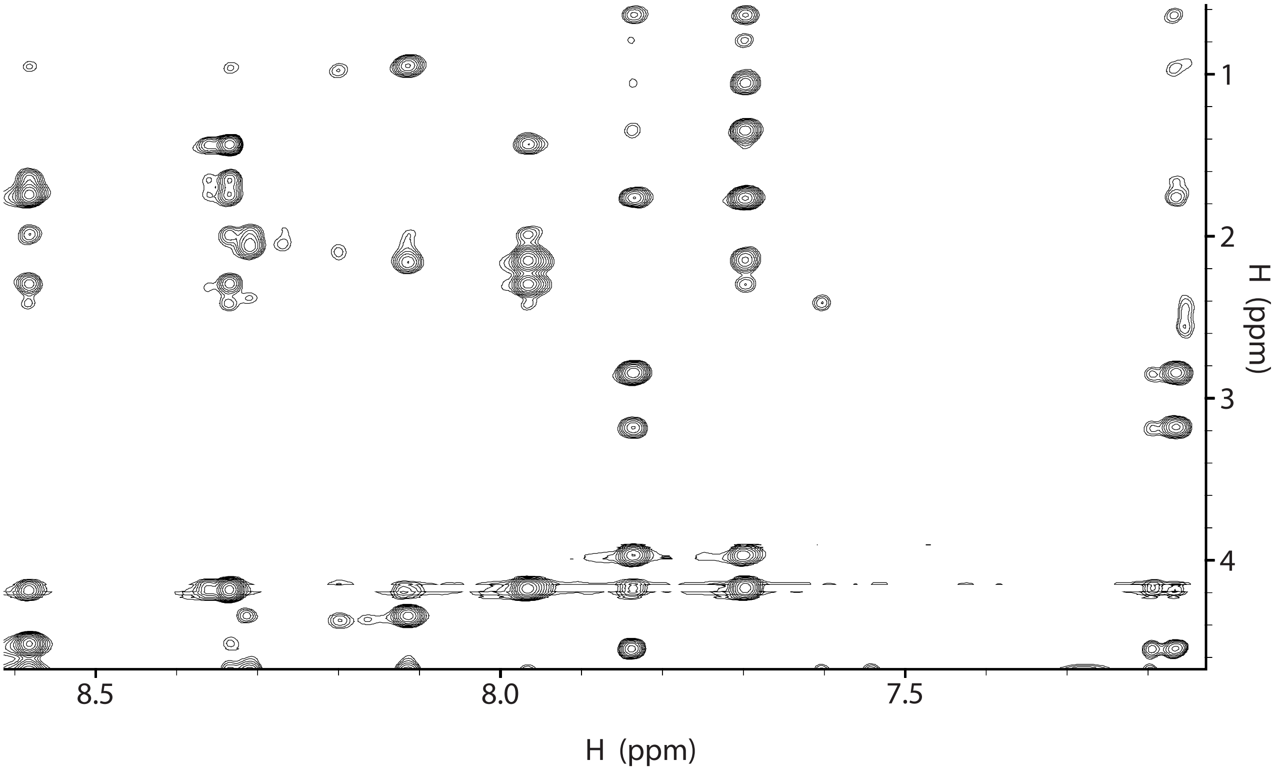


Figure S29: 700 MHz finger print region of NOESY spectrum (293K) of AcQVA$^{NH}$RQLAEIY-$NH_2$, Peptide **5** in $D_2O$/TFE-$d_2$/PBS (5/30/65%v/v) at pH 7.4

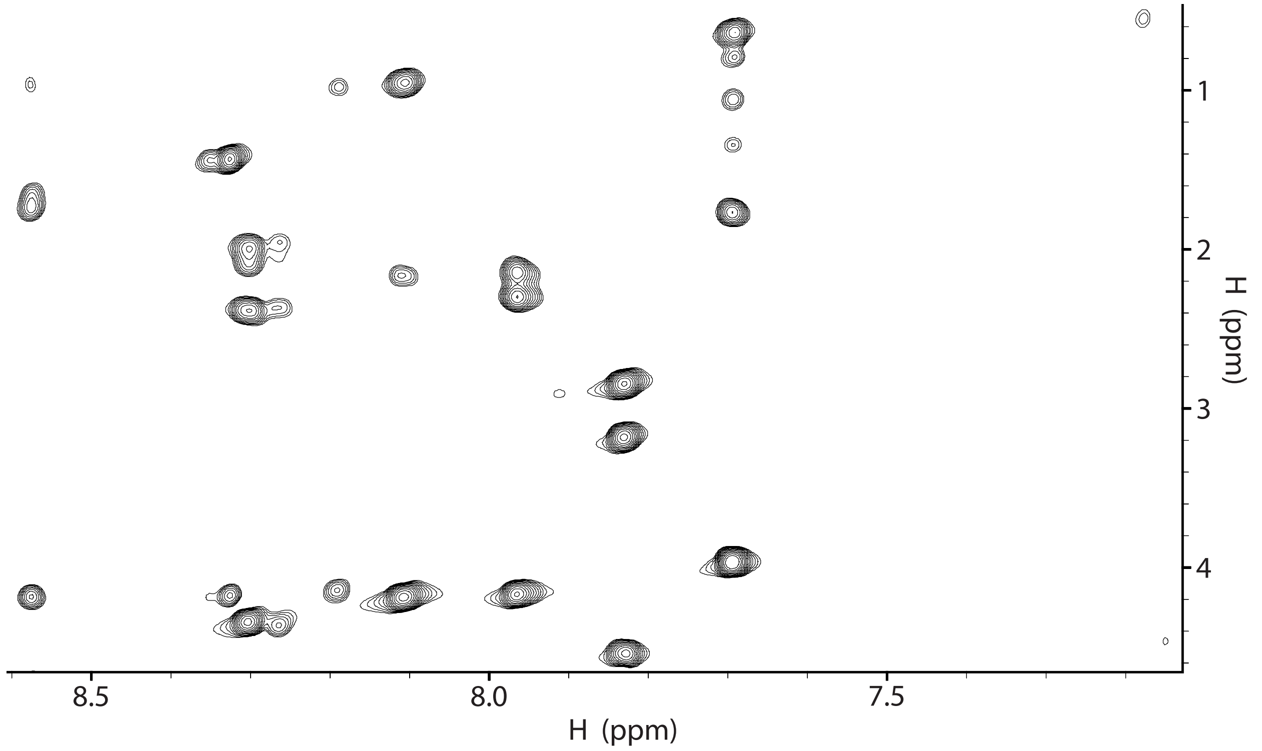


Figure S30: 700 MHz finger print region of TOCSY spectrum (293K) of AcQVA$^{NH}$RQLAEIY-$NH_2$, Peptide **5** in $D_2O$/TFE-$d_2$/PBS (5/30/65%v/v) at pH 7.4

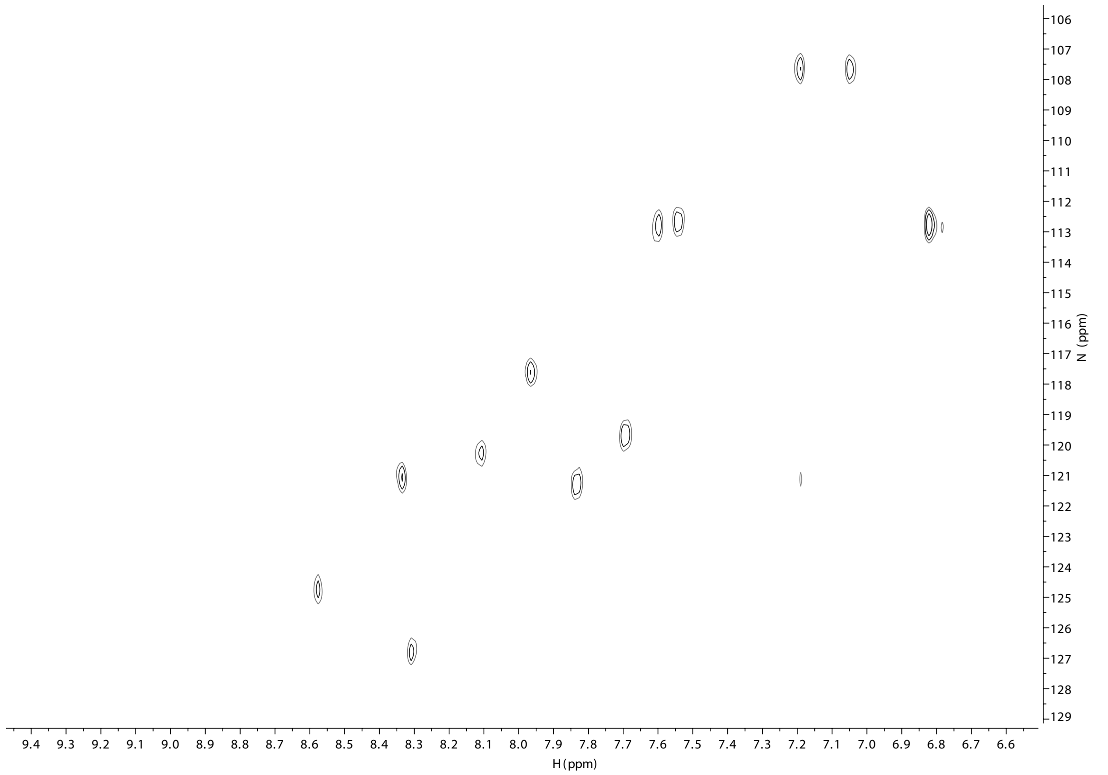


Figure S31: 700 MHz $^{15}$N-H HSQC spectrum (293K) of AcQVA$^{NH}$RQLAEIY-$NH_2$, Peptide **5** in $D_2O$/TFE-$d_2$/PBS (5/30/65%v/v) at pH 7.4

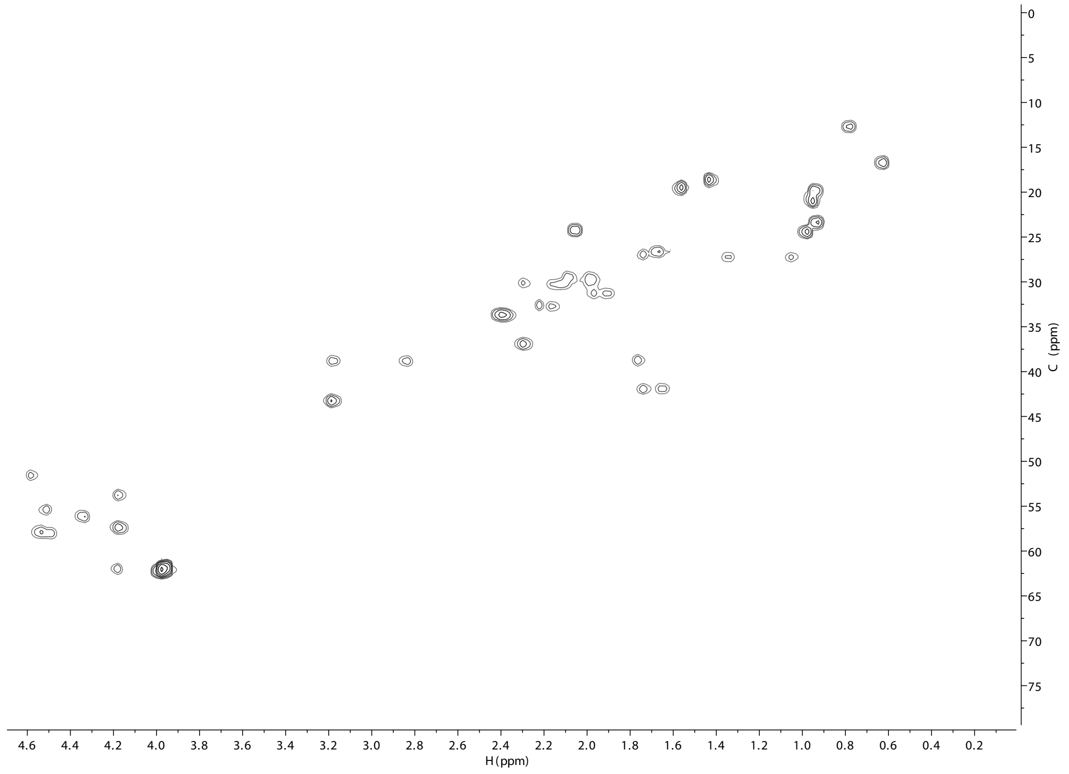


Figure S32: 700 MHz $^{13}$C-H HSQC spectrum (293K) of AcQVA$^{NH}$RQLAEIY-$NH_2$, Peptide **5** in $D_2O$/TFE-$d_2$/PBS (5/30/65%v/v) at pH 7.4

### 4.2.4 Example Spectra of additional NMR experiments

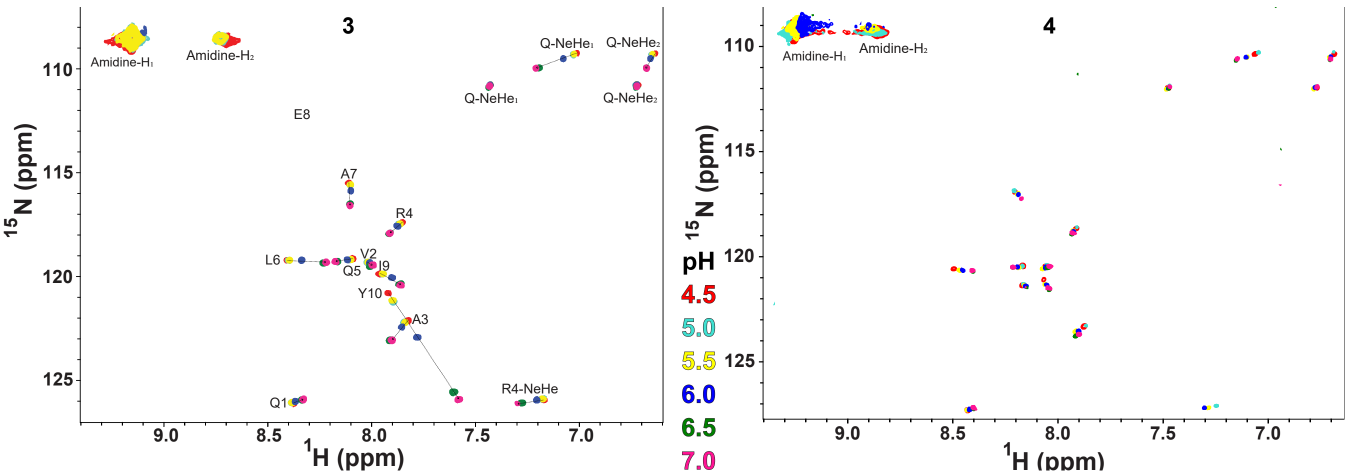


Figure S33: Overlay of pH titration NMR spectra at 20 ˚C for Peptide **2** and **4** in $D_2O$/TFE-$d_2$/PBS (5/30/65%v/v).

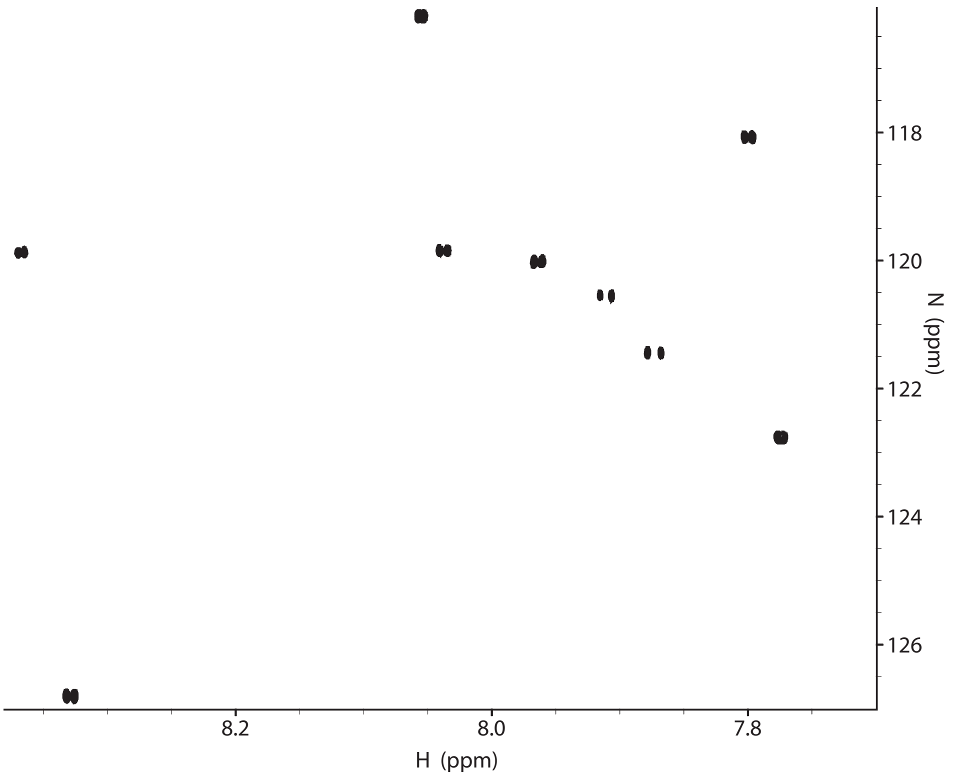


Figure S34: 800 MHz J-coupling experiment example spectra (293K) of AcQVARQLA$^{NH}$EIY-$CO_2$ Peptide **2** in $D_2O$/TFE-$d_2$/PBS (5/30/65%v/v) at pH 7.4.[6]

# 5 Structure Determination *via* NMR.

## 5.1 chemical shift in difference of $\alpha$-protons

The percent character of the observed by NMR for amidinopeptides were determined using the equations below:

$$\Delta\delta H_{\alpha} = (\delta H_{\alpha}(experimental)) - (\delta H_{\alpha}(randomcoil)) \quad (2)$$

$$\Delta ChemicalShift = (C\alpha_{\text{experimental}} - C\alpha_{\text{coil}}) - (C\beta_{\text{experimental}} - C\beta_{\text{coil}}) \quad (3)$$

Where, $\delta$ $H_{\alpha}$(experimental)), $C\alpha_{\text{experimental}}$, and $C\beta_{\text{experimental}}$ were experimentally determined from the NMR experiments (in ppm) and random coil chemical shifts were extracted using reported chemical shift by Wright and co-workers for the sequence QVARQLAEIY.[7] For $\Delta\delta$ $H_{\alpha}$ calculations show positive and negative differences represent sheet and helix, respectively (Figure S42).

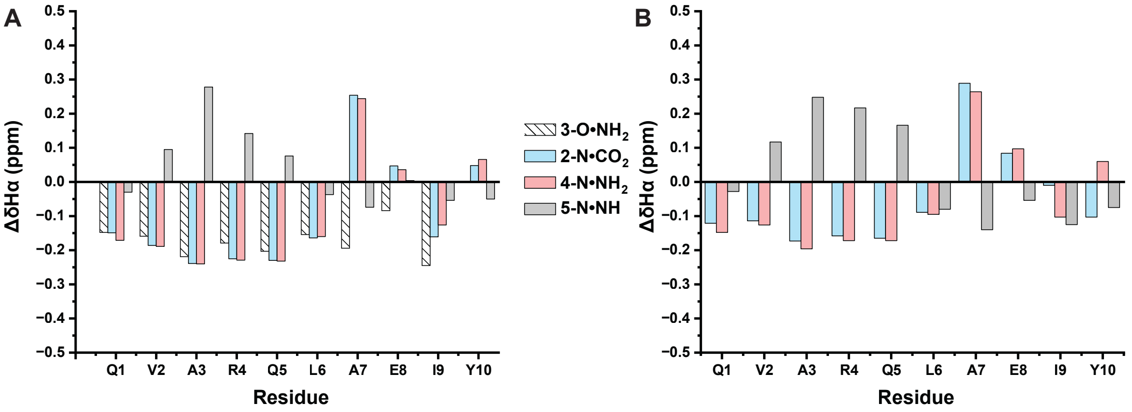


**Figure S35: A) Comparison of peptides 2, 3, 4, and 5 at pH 4.5. B) Comparison of peptides 2, 4, and 5 at pH 7.4. All using the $\Delta\delta H_{\alpha}$ chemical shifts (equation 2).**

## 5.2 J-coupling experiments of amidinopeptides

Table S3: *J*-Coupling (Hz) of backbone amide protons for peptides and calculated $\varphi$ angle for amidinopeptides in main manuscript at 293K, pH 4.5 and 7.4 in $D_2O$/TFE-$d_2$/PBS (5/30/65 %v/v). Utilizing the $^1H$-$^{15}N$ TROSY-HSQC experiment adjusting the WATERGATE element,pulse sequence reported by Roche and coworkers.[6]

| Residue | **2** $^3J$, $\varphi$ | **3** $^3J$, $\varphi$ | **4** $^3J$, $\varphi$ | **5** $^3J$, $\varphi$ | Residue | **2** $^3J$, $\varphi$ | **4** $^3J$, $\varphi$ | **5** $^3J$, $\varphi$ |
|---|---|---|---|---|---|---|---|---|
| pH 4.5 | | | | | pH 7.4 | | | |
| Y10 | 8.61, -94 | 7.55, -88 | 8.59, -94 | 7.06, -84 | Y10 | 8.27, -92 | 8.41, -92 | 7.82, -90 |
| I9 | 6.67, -80 | 6.82, -82 | 6.88, -82 | 6.26, -77 | I9 | 7.26, -85 | 6.85, -82 | 7.04, -84 |
| E8 | 6.15 , -75 | 4.73, -65 | -, - | 6.26, -77 | E8 | -, - | -, - | 6.28, -75 |
| A7 | 3.50, -55 | 4.29, -61 | 3.52, -55 | 4.33, -62 | A7 | 2.53, -45 | 3.72, -57 | 3.15, -53 |
| L6 | 3.52, -55 | 3.60, -56 | 3.32, -53 | 5.08, -67 | L6 | 5.1, -67 | 4.50, -63 | 3.50, -55 |
| Q5 | 4.70, -63 | 5.01, -66 | 4.70, -63 | 5.48, -70 | Q5 | 5.47, -70 | 5.28, -69 | -, - |
| R4 | 5.08, -67 | 3.91, -60 | 4.32, -63 | -, - | R4 | 4.50, -63 | 4.5, -63 | -, - |
| A3 | 3.53, -55 | 4.10, -62 | 3.52, -55 | 2.96, -51 | A3 | 3.72, -57 | 3.92, -59 | -, - |
| V2 | 5.08, -67 | 5.01, -66 | 5.48, -70 | 6.65, -79 | V2 | 5.48, -70 | 5.48, -70 | 6.69, -79 |
| Q1 | 4.70, -64 | 4.75, -64 | 5.07, -67 | 6.28, -77 | Q1 | 5.89, -74 | 5.46, -70 | 5.87, -74 |

### 5.3 NOE cross peak correlations for Amidinopeptides

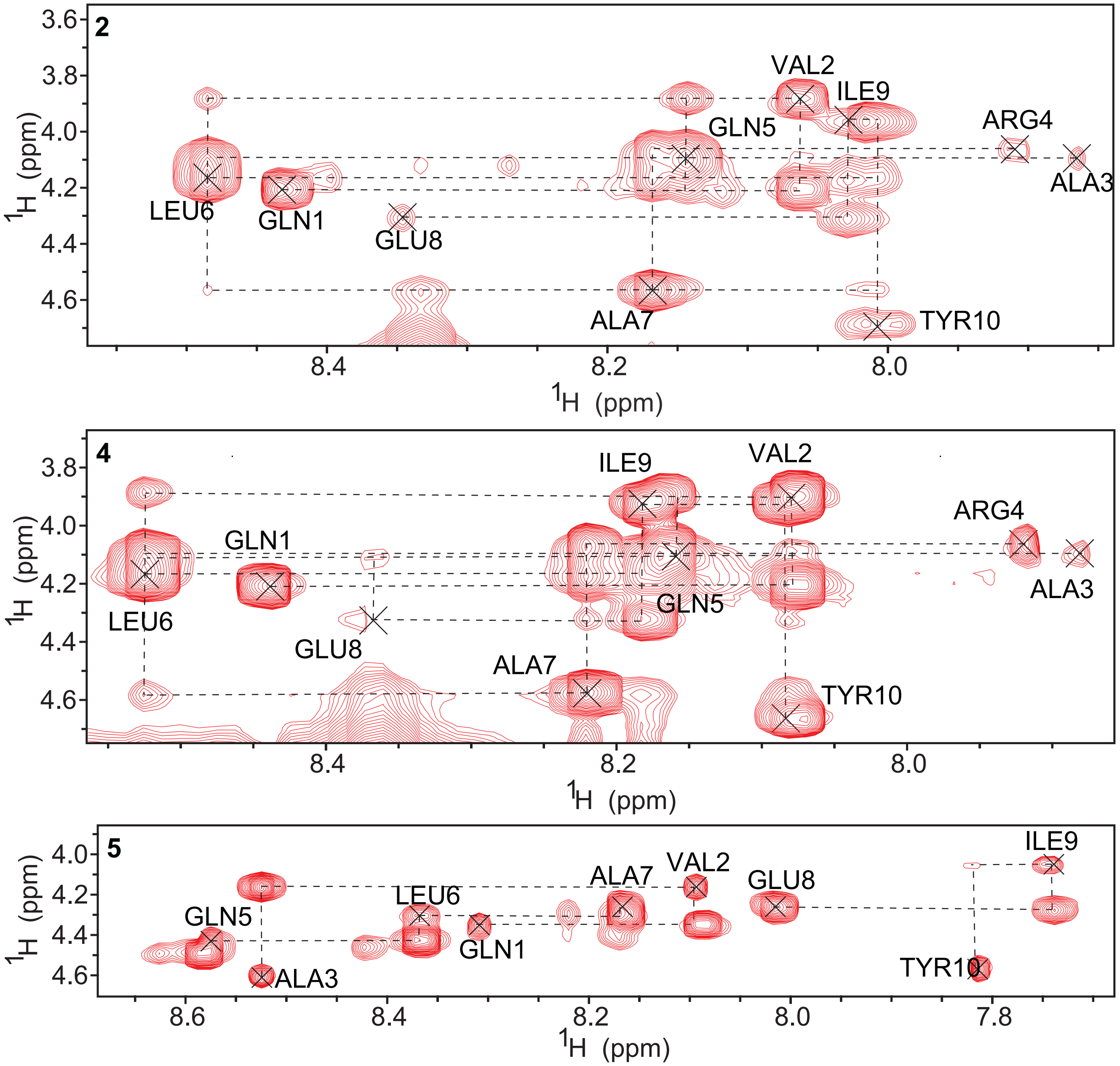


Figure S36: observable NOE correlation cross-peaks along the backbone for non-sequential medium range NOEs, $d\alpha$N ($i$,$i$+3) and $d\alpha$N ($i$,$i$+4) cross peaks to support $\alpha$-helix for amidinopeptide at pH 4.5.

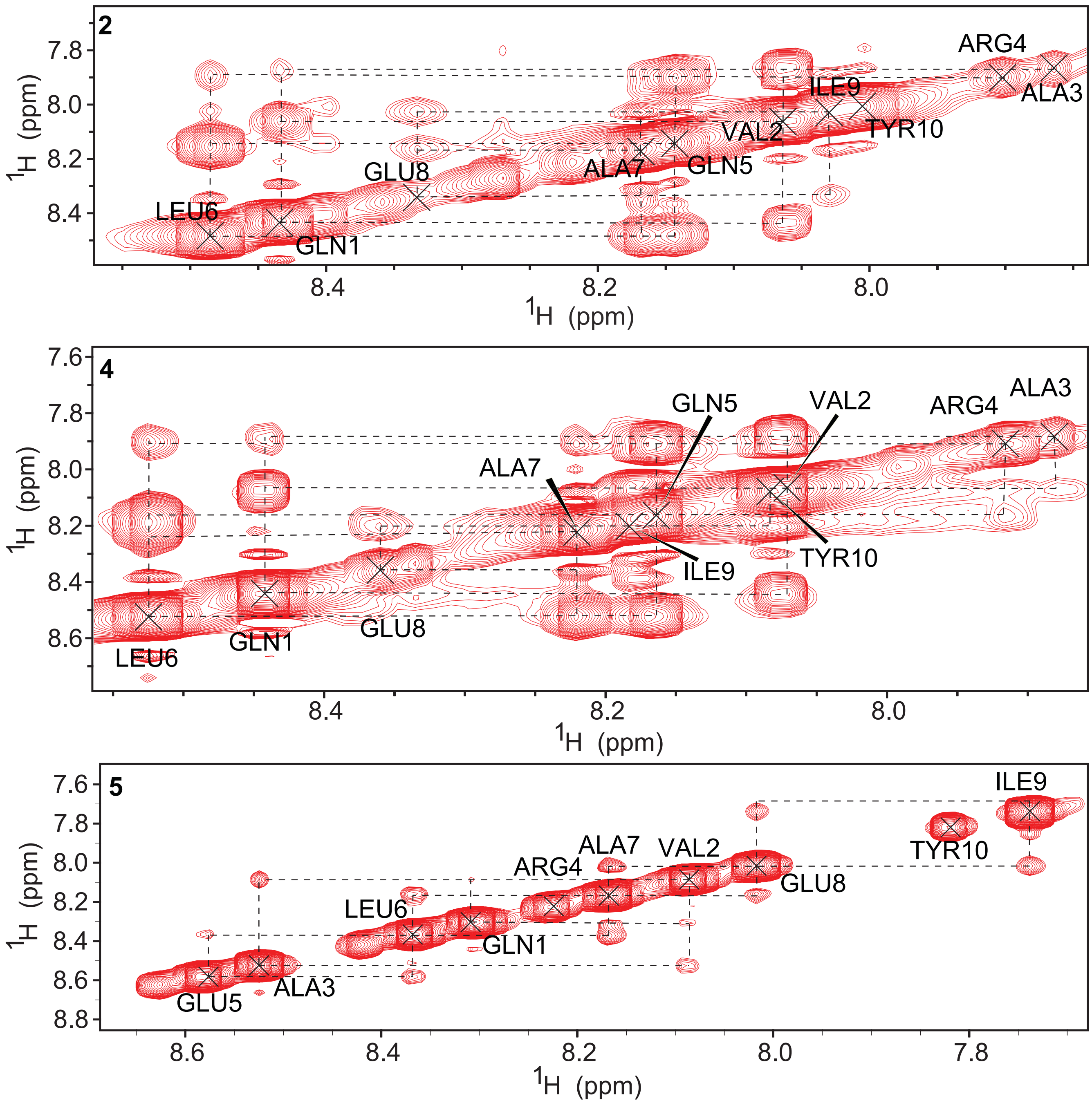


Figure S37: observable NOE correlation cross-peaks along the backbone for sequential NN ($i$,$i$+1) and $d\alpha$N ($i$,$i$+1) cross peaks to support $\alpha$-helix for amidinopeptide at pH 4.5.

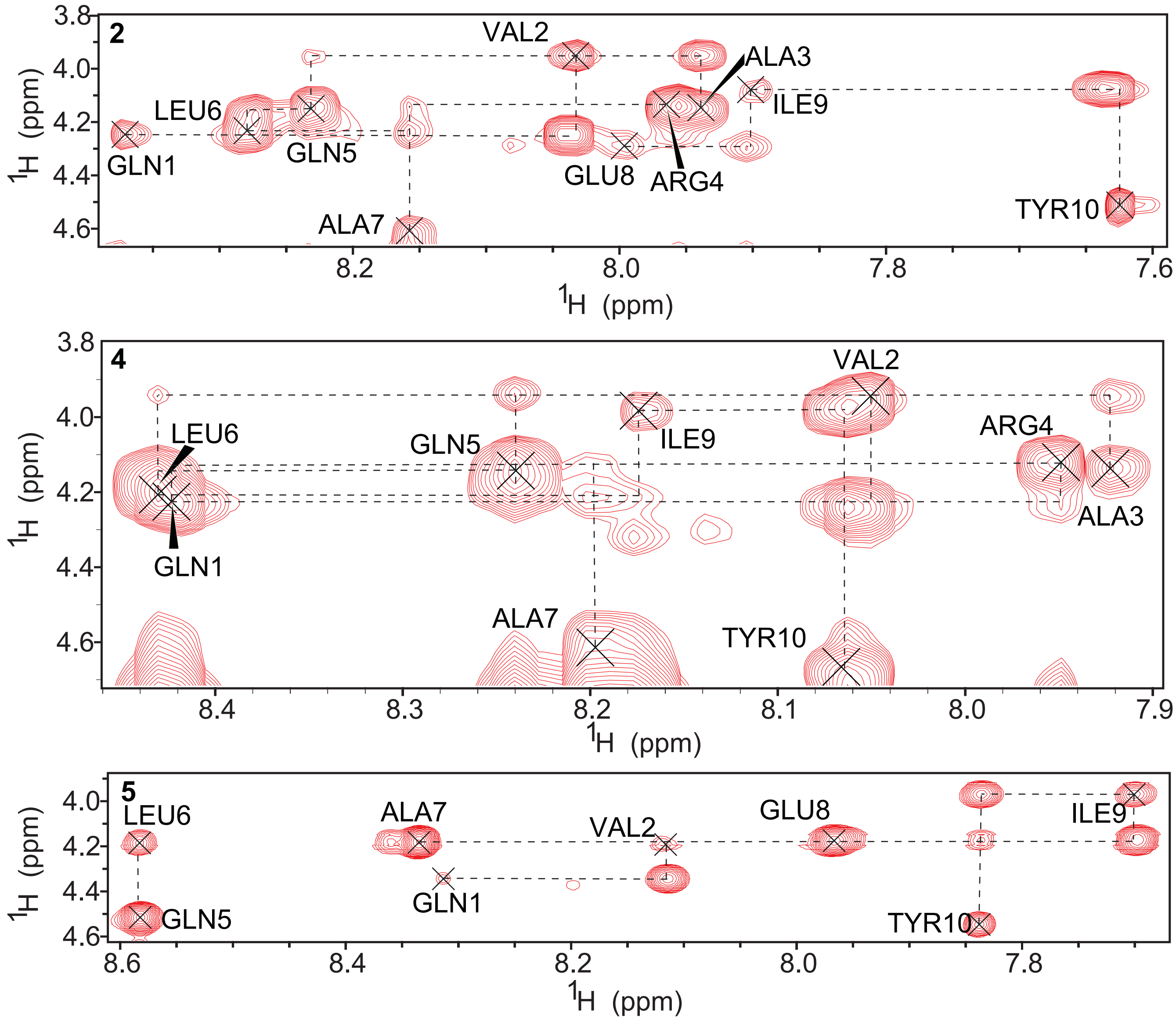


Figure S38: observable NOE correlation cross-peaks along the backbone for sequential NN (*i*,*i*+1) and $d\alpha$N (*i*,*i*+1) cross peaks to support $\alpha$-helix for amidinopeptide at pH 7.4.

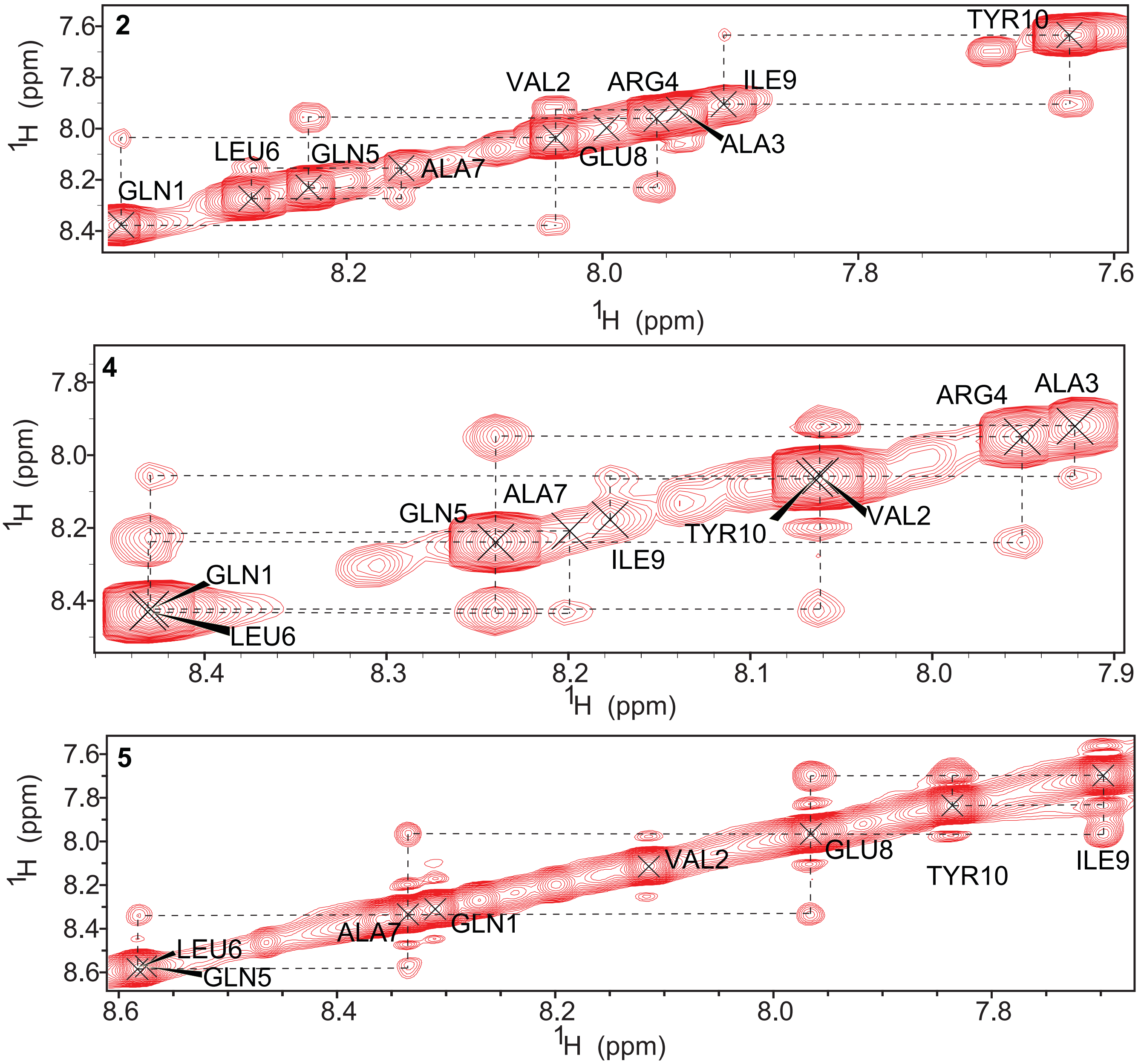


Figure S39: observable NOE correlation cross-peaks along the backbone for non-sequential medium range NOEs, $d\alpha N$ ($i$,$i$+3) and $d\alpha N$ ($i$,$i$+4) cross peaks to support $\alpha$-helix for amidinopeptide at pH 7.4.

# 6 HPLC Traces of Purified Peptides

## 6.1 purity check traces of peptides

Table S4: Eluent Gradient Conditions for Below HPLC Traces

| Time (min.) | % A | % B |
|---|---|---|
| 0 | 100 | 0 |
| 30 | 0 | 90 |
| 30.01 | 0 | 100 |
| 32.50 | 0 | 100 |
| 32.51 | 100 | 0 |
| 35.00 | 100 | 0 |

Flow rate held constant at 1 ml/min.
A is Water with 0.1% TFA, B is MeCN with 0.1% TFA

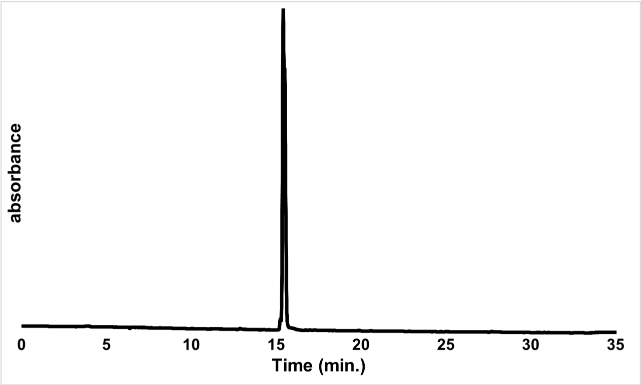


**Figure S40: HPLC Trace of Peptide 1 AcQVARQLAEIY-OH monitored at 254 nm.**
**HRMS: Found 1231.6175 m/z, calculated for $C_{55}H_{90}N_{15}O_{17}^{+H}$ 1231.6561 m/z**

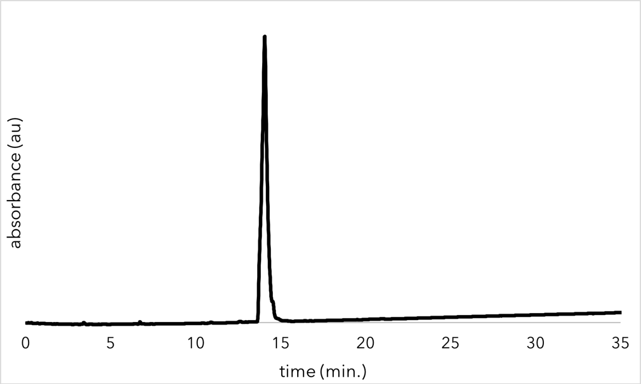


**Figure S41: HPLC Trace of Peptide 2 AcQVARQLA$^{NH}$EIY-OH monitored at 254 nm.**
**HRMS: Found 616.3305 m/z and 1231.6792 m/z, calculated for $C_{55}H_{92}N_{16}O_{16}^{+2H}$, $C_{55}H_{91}N_{16}O_{16}^{+H}$ 616.3431 m/z and 1231.6794 m/z, respectively.**

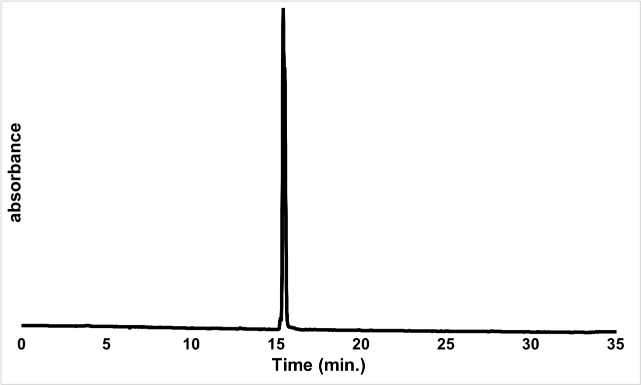


**Figure S42: HPLC Trace of Peptide 3 AcQVARQLAEIY-$NH_2$ monitored at 254 nm.**
**HRMS: Found 1231.6779 m/z, calculated for $C_{55}H_{91}N_{16}O_{16}^{+2}$ 1231.6794 m/z.**

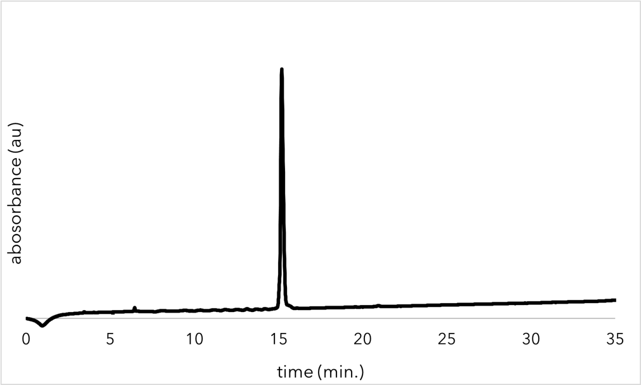


**Figure S43: HPLC Trace of Peptide 4 AcQVARQLA$^{NH}$EIY-$NH_2$ monitored at 254 nm.**
**HRMS: Found 615.8528 m/z and 1230.6992 m/z, calculated for $C_{55}H_{92}N_{16}O_{16}^{+2H}$, $C_{55}H_{91}N_{17}O_{16}^{+H}$ 615.8513 m/z and 1230.6953 m/z, respectively.**

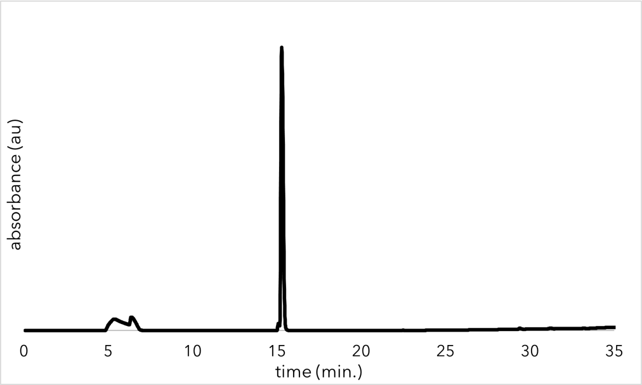


**Figure S44: HPLC Trace of Peptide 2 isotopically labeled AcQVARQLA$^{15NH}$EIY-OH monitored at 254 nm.**
**HRMS: Found 616.8443 m/z and 1232.6788 m/z, calculated for $C_{55}H_{92}N_{15^{15}N}O_{16}^{+2H}$, $C_{55}H_{91}N_{15}{}^{15}NO_{16}^{+H}$ 616.8469 m/z and 1232.6866 m/z, respectively.**

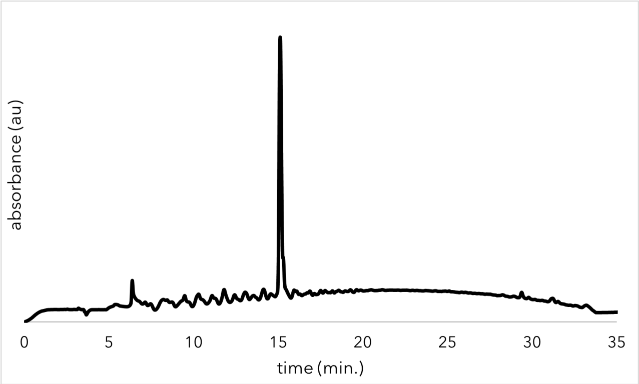


**Figure S45: HPLC Trace of Peptide 4 isotopically labeled AcQVARQLA$^{15NH}$EIY-$NH_2$ monitored at 254 nm.
HRMS: Found 616.3440 m/z and 1231.6835 m/z, calculated for $C_{55}H_{92}N_{16}{}^{15}NO_{16}{}^{+2H}$, $C_{55}H_{91}N_{16}{}^{15}NO_{16}{}^{+H}$ 616.3549 m/z and 1231.6926 m/z, respectively.**

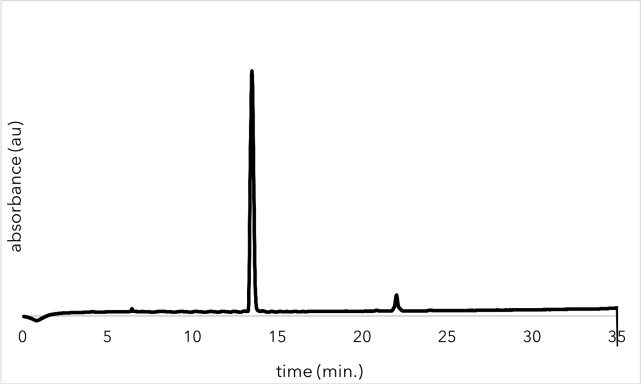


**Figure S46: HPLC Trace of Peptide 5 AcQVA$^{NH}$RQLAEIY-$NH_2$ monitored at 254 nm.
HRMS: Found 616.3431 m/z and 1231.6797 m/z, calculated for $C_{55}H_{92}N_{16}{}^{15}NO_{16}{}^{+2H}$, $C_{55}H_{91}N_{16}{}^{15}NO_{16}{}^{+H}$ 616.3549 m/z and 1231.6926 m/z, respectively.**

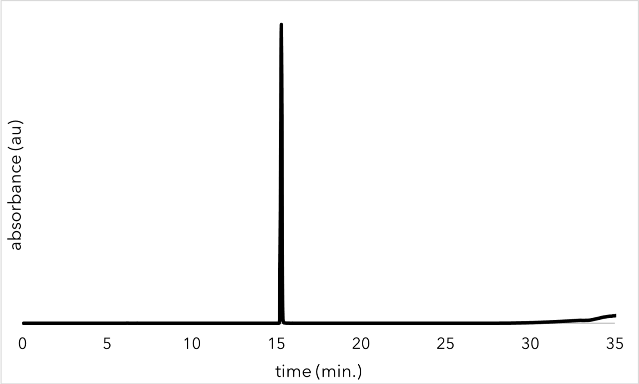


**Figure S47: HPLC Trace of Peptide 5 isotopically labeled AcQVA$^{15NH}$RQLAEIY-$NH_2$ monitored at 254 nm.
HRMS: Found 616.3494 m/z and 1231.6835 m/z, calculated for $C_{55}H_{92}N_{16}{}^{15}NO_{16}{}^{+2H}$, $C_{55}H_{91}N_{16}{}^{15}NO_{16}{}^{+H}$ 616.3549 m/z and 1231.6926 m/z, respectively.**

## 6.2 Stability of Amidinopeptide in Water.

Table S5: Eluent Gradient Conditions for UPLC Trace of **4** and **2-N·OH** to test stability in water.

| Time (min.) | %A | %B |
|---|---|---|
| 0.00 | 95 | 5 |
| 6.00 | 5 | 95 |
| 6.01 | 0 | 100 |
| 7.00 | 0 | 100 |
| 7.01 | 95 | 5 |
| 8.00 | 95 | 5 |

Flow rate held constant at .450 ml/min.

A is Water with 0.1% Formic Acid, B is Acetonitrile with 0.1% Formic Acid

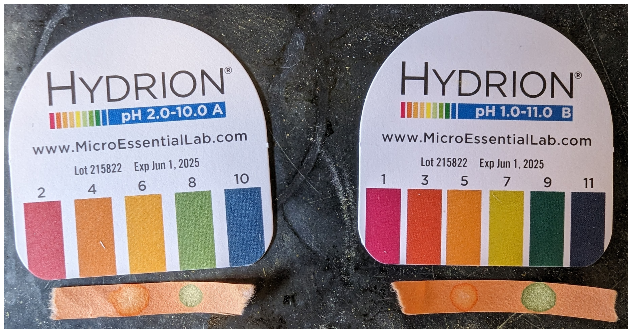


**Figure S48: pH of Peptide 4 to be between 6-7 according to the pH strips shown. Left spot is of Peptide 4 in solution, and the right spot is a solution of sodium bicarbonate.**

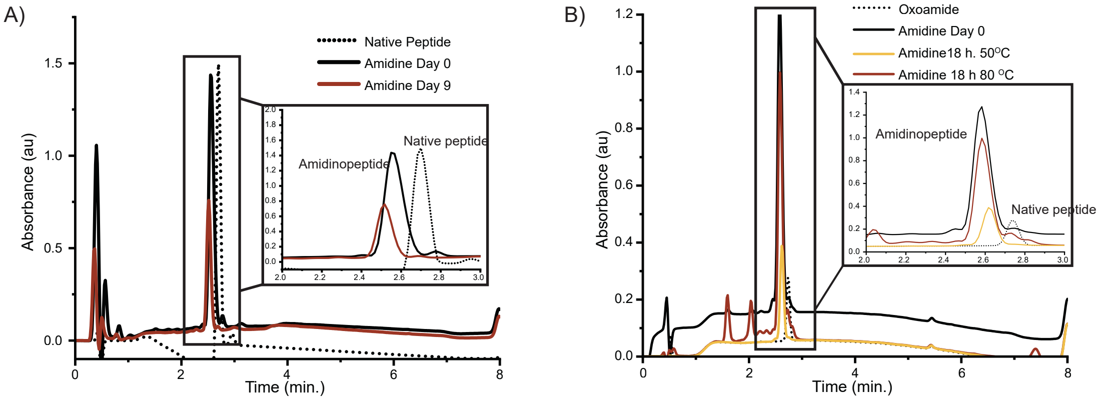


**Figure S49: UPLC trace using gradient from Table S5 of A) peptide 4 initial (black) and after sitting in a water solution with a pH from 6-7 for 9 days (red). Showing little to no hydrolysis of the amidinopeptide and formation of native oxoamide peptide (3, dotted black trace). B) peptide 2-N·OH initial (black) and after sitting in a water solution with a pH from 6-7 for at temperatures of 50°C and 80°C. Showing little hydrolysis of the amidinopeptide and formation of native oxoamide peptide (1-O·OH, dotted black trace) at these condition, specifically at the higher temperature of 80°C. These observation at slightly milder conditions than what has been reported in the literature from conversion of amidines to amide.[8–10]**

## 6.3 Absorbance Difference of Acetamide and Acetamidine

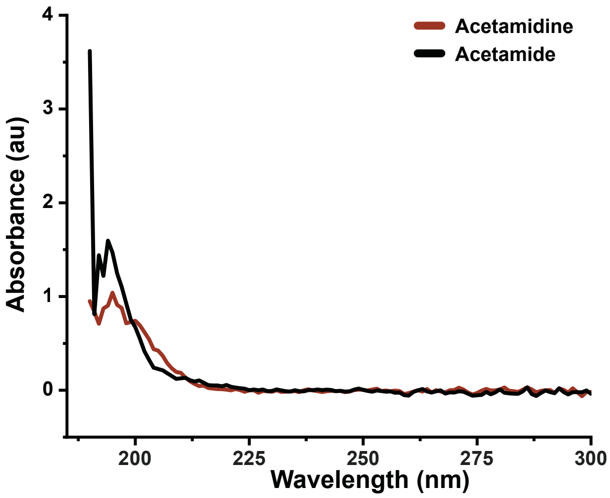


**Figure S50: UV-VIS absorption spectra of Acetamide (Black) vs Acetamidine (Red).**

Ultraviolet–visible absorption spectra were measured with an Agilent Technologies Cary 8454 UV–VIS diode array system and corrected for background signal with a cuvette containing the same solvent used for analysis. Samples were prepared through a serial dilution of a 1 *M* solution (in DI water) of both acetamide and acetamidine. The 1 m*M* sample UV-Vis spectra were compared to confirm the amidine did not have red shifted absorption. Acetamidine was purchased from Ambeed as a HCl, the pH (6.5) was recorded. Displaying similar absorption between the isosteres, confirming the CD traces are not impacted by the absorption of the amidine motif.

## 7 Procedure for p$K_a$ determination of 6

**General Procedure.** Compound (imidazole or **6**) was dissolved in DI water to produce a 40 m*M* concentrated solution. The pH meter was calibrated with standard pH solutions of 4, 7, and 10. Then the initial pH of the sample solutions were recorded. To the sample, 0.2 *M* HCl was added in 50 $\mu$L increments for imidazole and 5 $\mu$L for **6**, and the pH was recorded after each addition until it reached a pH of ca. 1.5-1.80. The pH and volume of 0.2 *M* HCl was plotted to achieve a titration curve, and the 1/2 equivalence point was distinguished to determine the p$K_a$ of the compounds. We observed that for imidazole the pH titration exhibited a p$K_a$ correlated with reported values of 6.9. For dimer (**6**) we obtained a p$K_a$ of around 5.6 (Figure 4, main text).

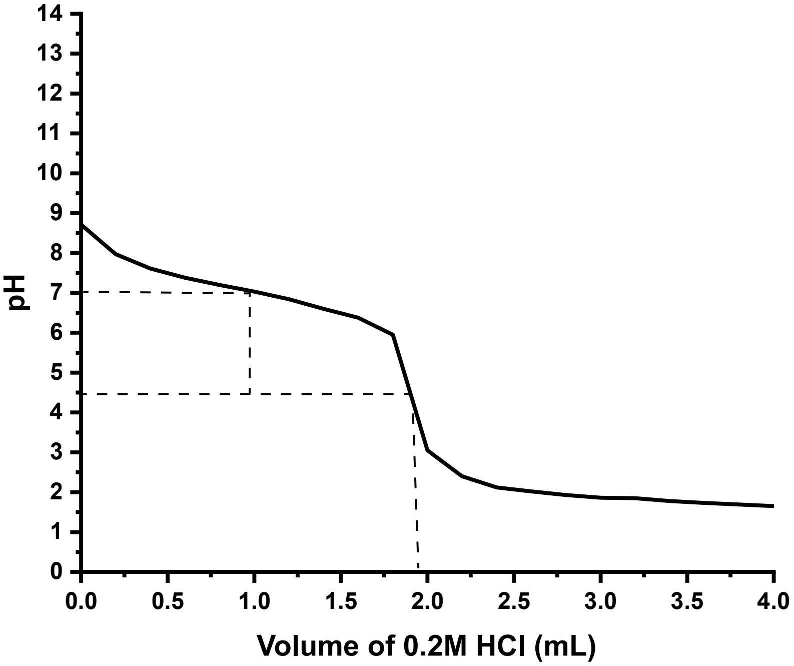


**Figure S51: pH titration curve for imidazole giving a p$K_a$H of 6.9. To standardize the method to determine the p$K_{aH}$ of 6.**

## 8 CD Experiments

CD spectra were recorded Jasco J-710 Cd spectrometer equipped with a temperature controller using 1mm length cell and scan speed of 5 mm/min. The spectra was averaged over 10 scans with the baseline subtracted from respected solvents conditions. Spectra were smoothed *via* the Lowess method with 0.1 span. The samples were prepared in 1x phosphate buffer saline at a pH of 7.4, and contained 0 to 30% of trifluoroethanol (TFE) as a helix stabilizing co-solvent. The concentration of each sample was determined by UV absorption of Tyr residue at 280 nm, extinction co-efficient of 1490 $cm^{-1}M^{-1}$[1], to give a final concentration ranging from 50-75 $\mu$M. The amount of helical character was determined from the mean residue ellipticity at 222 nm, $[\theta]_{222}$ (deg $cm^2$ $dmol^{-1}$) corrected for the number of amino acids. The percentage of helix was calculated from the ratio of $[\theta]_{222}/[\theta]_{max}$ where $[\theta]_{max}$ was calculated using the equation below:

$$\theta_{\text{max}} = \frac{-44000 + 250T}{1 - (k/n)} \tag{4}$$

Where T= 20°C, k= 4 and n= 10 (the number of amide linkages in the peptide). An additional study to probe an amidine linkage effect of the structure is determining the stability of the helix, which can be conducted by monitoring the intensity of the 222 nm band with a gradual increase in the temperature. Peptides **3** and **4** were compared due to **1** not having any structure to reference **2** too. Peptide **3** and **4** samples include 30% TFE as a co-solvent, as they were the samples with greatest observe character (Figure S56). Both samples were subjected to an increase of temperature from 5 to 80°C while recording the intensity at 222 nm. Both peptide were detected to ”unwind” their structure with increase in their molar ellipticity.

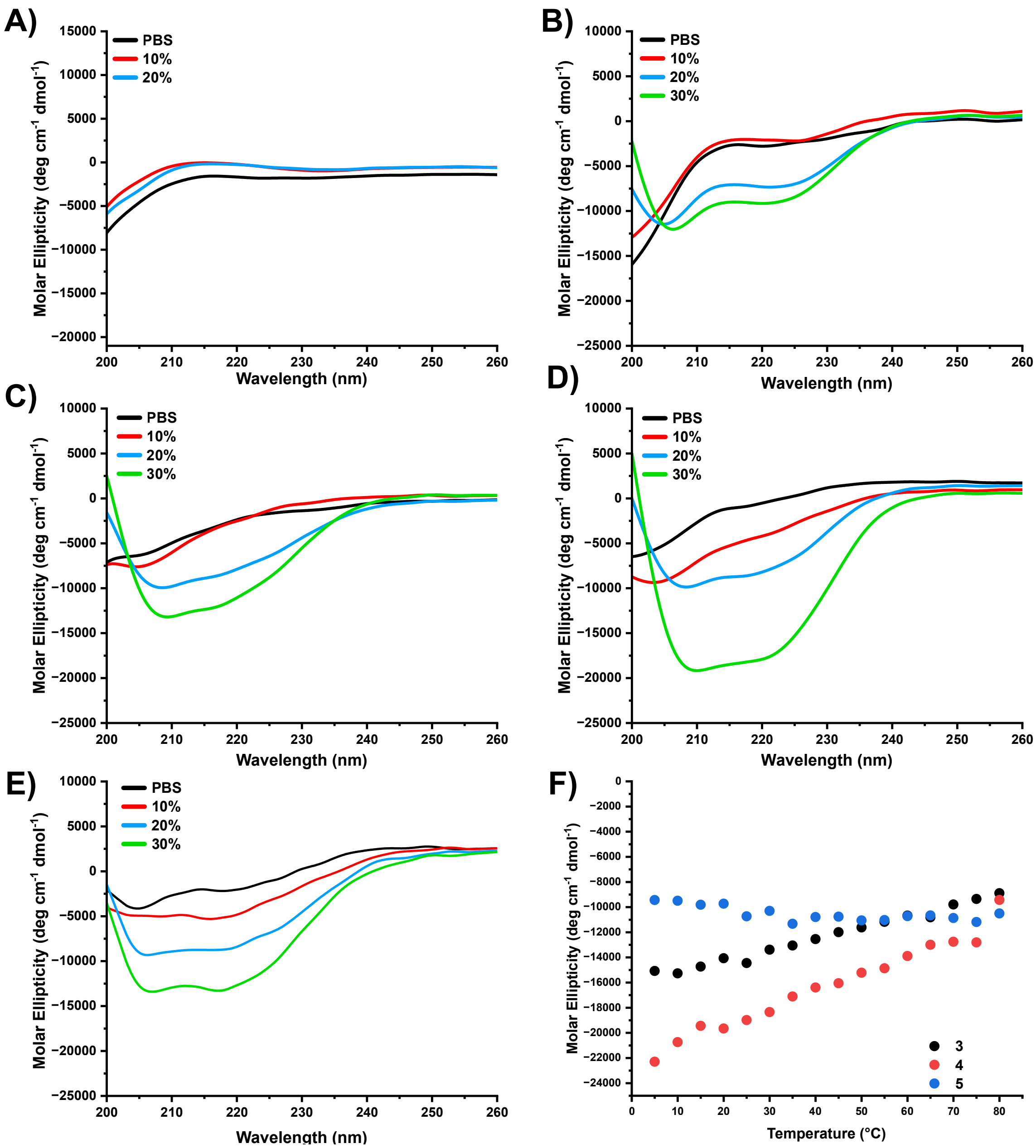


**Figure S52: CD Spectra of A) peptide 1 vs B) peptide 2, C) peptide 3-O·$NH_2$ vs D) peptide 4-N·$NH_2$. and E) peptide 5. F) thermal decomposition of amidinopeptides (4-N·$NH_2$ and 5).**

# 9 References


(1) Anthis, N. J.; Clore, G. M. Sequence-specific determination of protein and peptide concentrations by absorbance at 205 nm. *Pro. Sci.* **2013**, *22*, 851–858.

(2) Shalaby, M. A.; Grote, C. W.; Rapoport, H. Thiopeptide Synthesis. Î±-Amino Thionoacid Derivatives of Nitrobenzotriazole as Thioacylating Agents. *J. Org. Chem.* **1996**, *61*, PMID: 11667896, 9045–9048.

(3) Khatri, B.; Bhat, P.; Chatterjee, J. Convenient synthesis of thioamidated peptides and proteins. *J. Pep. Sci.* **2020**, *26*, e3248.

(4) Camacho, L. A.; Lampkin, B. J.; VanVeller, B. A Bottom-Up Approach To Preserve Thioamide Residue Stereochemistry during Fmoc Solid-Phase Peptide Synthesis. *Org. Lett.* **2019**, *21*, PMID: 31403302, 7015–7018.

(5) O'Brien, E. A.; Sharma, K. K.; Byerly-Duke, J.; Camacho 3rd, L. A.; VanVeller, B. A general strategy to install amidine functional groups along the peptide backbone. *J. Am. Chem. Soc.* **2022**, *144*, 22397–22402.

(6) Roche, J.; Ying, J.; Bax, A. Accurate measurement of 3 J HNH$\alpha$ couplings in small or disordered proteins from WATERGATE-optimized TROSY spectra. *J. Biomol. NMR* **2016**, *64*, 1–7.

(7) Merutka, G.; Jane Dyson, H.; Wright, P. E. Random coil 1 H chemical shifts obtained as a function of temperature and trifluoroethanol concentration for the peptide series GGXGG. *J. Biomol. NMR* **1995**, *5*, 14–24.

(8) D'hooghe, M.; Van Nieuwenhove, A.; Van Brabandt, W.; Rottiers, M.; De Kimpe, N. Novel synthesis of 2-aminopentanedinitriles from 2-(bromomethyl) aziridines and their transformation into 2-imino-5-methoxypyrrolidines and 5-methoxypyrrolidin-2-ones. *Tetrahedron* **2008**, *64*, 1064–1070.

(9) Tan, E. T. T.; Yong, K. W. L.; Wong, S.-H.; D'Arcy, B. R.; Al Jassim, R.; De Voss, J. J.; Fletcher, M. T. Thermo-alkaline Treatment as a Practical Degradation Strategy To Reduce Indospicine Contamination in Camel Meat. *J. Ag. Food Chem.* **2016**, *64*, PMID: 27737547, 8447–8453.

(10) Romagnoli, R.; Baraldi, P. G.; Pavani, M. G.; Fruttarolo, F.; Preti, D.; Bovero, A.; AGHAZADEH TABRIZI, M.; Bianchi, N.; Gambari, R., et al. Design, synthesis and growth inhibition activity of bis-epoxyethyl derivatives of stallimycin modified on the amidino moiety. *Med. Chem. Res.* **2004**, *13*, 282–296.
